# Association of dietary and circulating antioxidant vitamins with metabolic syndrome: an observational and Mendelian randomization study

**DOI:** 10.3389/fendo.2024.1446719

**Published:** 2024-10-14

**Authors:** Qian Sun, Zhixing Fan, Fangfang Yao, Xiaojing Zhao, Min Jiang, Mudan Yang, Menglu Mao, Chaojun Yang

**Affiliations:** ^1^ Department of Clinical Laboratory, The Affiliated LiHuiLi Hospital of Ningbo University, Ningbo, China; ^2^ Department of Cardiology, The First College of Clinical Medical Sciences, China Three Gorges University, Yichang, China; ^3^ Department of Medical Record Management, The First College of Clinical Medical Sciences, China Three Gorges University, Yichang, China; ^4^ Clinical Laboratory, Ningbo Yinzhou No.2 Hospital, Ningbo, China; ^5^ School of Foreign Studies, China Three Gorges University, Yichang, China

**Keywords:** antioxidant vitamins, metabolic syndrome, obesity, hypertension, NHANES, Mendelian randomization

## Abstract

**Aims:**

The objective of this study was to investigate the associations of dietary and circulating antioxidant vitamins with metabolic syndrome (MetS), and to assess causality using Mendelian randomization (MR).

**Methods:**

This study included 10,308 participants from the National Health and Nutrition Examination Survey. The associations of vitamins A, C, E and carotenoids with MetS were assessed using multivariable weighted logistic regression analysis. Subsequently, the MR approach was employed to test the causal associations, with inverse variance weighted (IVW) serving as the primary analysis.

**Results:**

Observationally, dietary vitamin A (OR=0.852, 95%CI: 0.727-0.999), C (OR=0.802, 95%CI: 0.675-0.952), carotene (OR=0.832, 95%CI: 0.706-0.982), and β-carotene (OR=0.838, 95%CI: 0.706-0.995) in quartile 4 had lower incidents of MetS, when compared to quartile 1. Circulating vitamin C and carotene were also present inversely associated with MetS, while the vitamin A and E both increased this risk. IVW-MR confirmed the associations of dietary vitamin A (OR=0.920, 95%CI: 0.861-0.984), vitamin C (OR=0.905, 95%CI: 0.836-0.979) and carotene (OR=0.918, 95%CI: 0.865-0.974) with MetS. However, there was only circulating β-carotene (OR=0.909, 95%CI: 0.857-0.965) was found to be causally associated with MetS.

**Conclusions:**

Observational and MR studies have shown that adequate dietary intake of vitamin A, C and carotenoids may help to reduce the risk of MetS.

## 1 Introduction

Metabolic syndrome (MetS) is a group of metabolic abnormalities characterized by high blood glucose, abnormal blood lipids, high blood pressure, and abdominal obesity (1). The global prevalence of MetS ranges from 12.5% to 31.4%, with higher prevalence in the Eastern Mediterranean region and the Americas, and it increases with increasing national income levels (2). From 1999 to 2018, cardiovascular and metabolic indicators (blood glucose, lipids, blood pressure, and adiposity) continued to deteriorate in the United States, and the prevalence of MetS increased from 28.23% to 45.9% (3–5). Due to the complex state of metabolic dysregulation, MetS is an important risk factor for cardiovascular disease, diabetes, stroke, and death (6–9). MetS impose a heavy health economic burden on global public health systems, requiring urgent intervention.

Antioxidant vitamins are a class of vitamins with antioxidant properties, mainly including vitamin A, vitamin C, vitamin E and carotenoids, which can reduce the damage caused to cells by oxidative stress by scavenging oxygen free radicals. A study have shown that a prudent dietary pattern in adolescence, comprising fruits and vegetables, cereals, and legumes, has been associated with a significantly reduced risk of MetS in middle age, in comparison to a Western dietary pattern focused on meat, refined grains, processed, and fried foods (10). Fruits and vegetables in a prudent dietary pattern are rich in antioxidant vitamins, which may play an important role in reducing the incidence of MetS (11). However, the association between antioxidant vitamins and MetS is currently controversial. Some studies have suggested that taking antioxidant vitamins may reduce the risk of MetS (12, 13), while others have found no association (14). Some studies have found that people with MetS have significantly lower blood levels of antioxidant vitamins than healthy people (15, 16), while others have found the opposite (17, 18). In addition, due to the body’s metabolism of nutrients, serum antioxidant vitamins do not directly represent dietary intake. Whether they are consistent with metabolic syndrome remains unknown. Therefore, it is necessity to study the dietary and serum antioxidant vitamins separately in relation to metabolic syndrome.

Observational studies may be subject to confounding and reverse causality due to non-randomization, which limits the extrapolation and application of research findings. Mendelian randomization (MR) is a method of causal inference based on Mendelian segregation and the principle that alleles are randomly assigned in the process of gamete formation, using genetic variation as an instrumental variable (19). Because genetic variation is associated with outcomes in a causal temporal order and is unaffected by common confounders such as postnatal environment and social factors, MR can accurately infer the causal relationship between exposure and outcome (20). Li et al. (21) analyzed the relationship between antioxidant vitamins and MetS using MR, but less association was found. Therefore, this study aims to analyse the relationship between dietary and circulating antioxidant vitamins and MetS using an observational study and MR analysis.

## 2 Materials and methods

### 2.1 Overall study design and data sources

**Figure 1:**
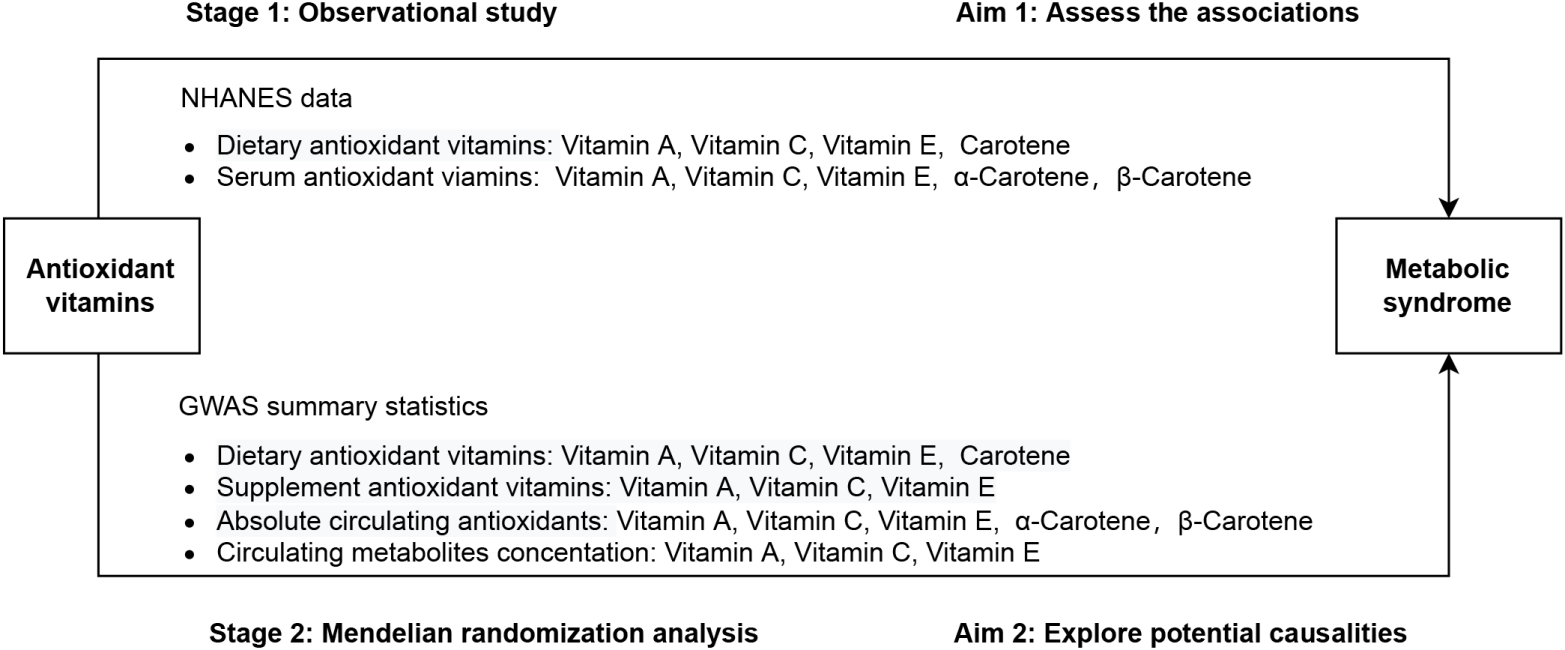
Overall study design based on observational analysis and Mendelian randomization.

The present study was conducted in two stages, as illustrated in **Figure 1**. In stage 1, using data deposited in the National Health and Nutrition Examination Survey (NHANES) database, we performed multivariable regression analysis to determine the observational association of dietary and serum antioxidant vitamins with MetS. In stage 2, we employed a two-sample MR analysis of summary statistics data from the genome-wide association study (GWAS) to assess the causal effect of genetically determined dietary and serum antioxidant vitamins on MetS.

The NHANES is a complex, multistage, nationally representative survey of the civilian noninstitutionalized population in the USA, conducted by the National Center for Health Statistics (NCHS). The survey includes household interviews, physical examinations, and laboratory tests. NHANES research protocols and data collection procedures were approved by the NCHS Research Ethics Review Board, and written informed consent was obtained from the participants1. The additional ethical review was no longer required for the present study due to the usage of publicly available data without identifiable personal information. Participants in NHANES were selected from 2001 to 2006 who were 20 years of age or older (n = 16,299). A total of 5,991 participants were excluded (**Supplementary Figure S1**): (1) Without 24-h dietary recall or missing dietary antioxidant vitamins (n=917); (2) Had no information of serum antioxidant vitamins (n=1,285); (3) Unable to be diagnosed as MetS or not (n=815); (3) Had no information of covariables (n = 2904).

The design of MR analysis needs to satisfy the following assumptions (**Supplementary Figure S2**) (22): (1) The SNPs employed as IVs are related to antioxidant vitamins; (2) IVs are not associated with the confounders; (3) IVs affect the risk of MetS only by antioxidant vitamins. The available summary GWAS data for MR analysis were obtained from the IEU open GWAS project [https://gwas.mrcieu.ac.uk/(accessed on 1 March 2024)], GWAS Catalog [https://www.ebi.ac.uk/gwas/(accessed on 1 March 2024)], or references. Further details on the GWAS datasets are provided in **Supplementary Table S1**. The summary data on dietary antioxidant vitamins were derived from the UK Biobank with 9,851,867 single-nucleotide polymorphisms (SNPs). The SNPs that were significantly associated with the absolute circulating antioxidants and circulating metabolite’s concentrations were obtained from published literature (23–28). The most comprehensive GWAS analysis was employed to obtain summary-level data for MetS, which included 291,107 individuals (59,677 cases and 231,430 controls) (29). Genetic information on components of MetS was also obtained, including fasting blood glucose (FBG), waistline, hypertension, triglycerides, high-density lipoprotein cholesterol (HDL-C) (30, 31).

### 2.2 Measurement of antioxidant vitamins

The intake of antioxidant vitamins was quantified through dietary interviews, including vitamin A, vitamin C, vitamin E, carotene, α-carotene, and β-carotene. In NHANES, dietary data were collected by 2-24 h dietary recalls (DRs), including the first day (Day 1) and second day (Day 2) which were collected in via the MEC and telephone, respectively. The in-person interview was conducted in the MEC dietary interview room, with participants reporting the amounts of foods consumed by means of a set of measuring guides (various glasses, bowls, mugs, household spoons, measuring cups and spoons, a ruler, thickness sticks, bean bags and circles). Telephone dietary interviews were self-reported by telephone, occurring 3 to 10 days after the MEC dietary interview. In UK Biobank, dietary data were from on-line dietary questionnaire, based on a 24-hour dietary recall of the previous day.

The serum levels of antioxidant vitamins were accessed using isocratic high-performance liquid chromatography (HPLC) with electrochemical detection. This consisted of vitamins A (retinol), vitamin C (ascorbate), vitamin E (α-tocopherol) and carotenoids. Sample collection, transformation, storage and analysis were conducted in accordance with the laboratory procedure manual.

### 2.3 Definition of metabolic syndrome

MetS was defined based on the National Cholesterol Education Program’s Adult Treatment Panel III (ATP III) as having 3 or more of the following (32): 1) FBG ≥5.6 mmol/L (or 100 mg/dL) or drug treatment for elevated blood glucose; 2) HDL-C <40mg/dL (or 1.0 mmol/L) in men or <50 mg/dL (or 1.3 mmol/L) in women or drug treatment for low HDL; 3) triglyceride level >150 mg/dL (or 1.7 mmol/L) or drug treatment for elevated triglyceride; 4) waist circumference >102 cm in men or >88 cm in women; 5) systolic blood pressure ≥ 130 mmHg, diastolic blood pressure ≥85 mmHg or taking hypertension medications.

### 2.4 Assessment of covariates

Information on demographic characteristics, lifestyle factors, and health conditions was obtained in NHANES. Sociodemographic characteristics consisted of age (20-40 years and 40-60 years), gender (women and men), race (non-Hispanic white, non-Hispanic black, Mexican American, other Hispanic and others), education (less than high school, high school graduate, some college and college graduate or above), marital status (married and others), poverty-income ratio (PIR: <1, 1-1.8, ≥1.8), health insurance (uninsured and any insurance). Lifestyle information was obtained from a series of questionnaires, including smoking status (never, former, now), drinking condition (yes and no), and physical activity (no, moderate activity and vigorous activity). The health conditions included in arteriosclerotic cardiovascular disease (ASCVD, consisted of coronary heart disease, angina, heart attack and stroke), chronic kidney disease (CKD), liver condition, thyroid disease, and cancer. All of these disease conditions were self-reported by the subjects, except for CKD, which was diagnosed according to KDIGO 2021 (33) (urine albumin to creatinine ratio of 30 mg/g or higher, or estimated glomerular filtration rate less than 60mL/min/1.73m).

### 2.5 Statistical analysis

NHANES data were extracted and pre-processed by the “nhanesR package” for the observational association of each antioxidant vitamins wit MetS. Sample weights were calculated according to NHANES tutorials for combining NHANES data from three cycles. A survey-weighted algorithm was employed for statistical analysis, considering complex sampling. The distribution of social demographics, lifestyle, and health conditions was expressed by numbers (unweighted) and percentage (weighted). The dietary intake and serum level of antioxidant vitamins were presented as the median and interquartile range [M, (P25, P75)] due to a skewed distribution. The difference between the groups was compared by Kruskal-Wallis’ test for continuous variables with non-normal distribution, and χ^2^ test for categorical variables. The correlation between dietary antioxidants and serum levels was analyzed by Spearman. Dietary and serum antioxidant vitamins were divided into four groups according to quartile. A survey-weighted Logistics regression model was employed to evaluate the odds ratio (OR) and 95% confidence interval (CI) for the relationship between each antioxidant vitamins and MetS. Four statistical models were fitted, with Model 1 not adjusting for any factors, Model 2 adjusting for social demographic factors, Model 3 further adjusting for lifestyle factors, and Model 4 additionally adjusting for the health conditions. We performed tests for linear trend by entering the median value of each quartile of antioxidant vitamins as a continuous variable in the models. To investigate dose-response association between each antioxidant vitamins and MetS, a restricted cubic spline (RCS) regression model was fitted by Logistics regression Logistics regression model with three knots (10^th^, 50^th^, and 90^th^ percentile). Tests for nonlinearity were performed using the likelihood ratio test. The relationship between antioxidant vitamins and components of MetS was also analyzed.

Two-sample MR analyses were performed using the “TwoSampleMR package” to investigate the causal analyses. In order to satisfy the core assumption of tool variables, three steps were carried out to filter IVs. Firstly, *P* < 5×10^-6^ was used as the primary screening criterion to ensure sufficient SNPs associated with exposure. Secondly, in order to ensure the independence of IVs, SNPs with linkage disequilibrium (LD) (*r* ^2^ < 0.001) were eliminated based on European ancestry reference data from the 1000 Genomes Project. Thirdly, IVs that showed genome-wide association with outcomes at a significance level of 5×10^-8^ were excluded. Additionally, palindromic SNPs were excluded after conducting harmonizing processes. *F* statistics were employed to assess the strength of weak instrumental variables (*F*>10 indicating the stronger instrument strength). Subsequently, the inverse variance weighted (IVW) method was utilized as the primary statistical method in MR to ascertain the causal relationship between antioxidant vitamins and MetS. Additionally, the MR Egger, weighted median, simple mode, and weighted mode were applied as sensitivity analyses. The MR-Egger method was employed to assess heterogeneity and pleiotropy. If the P-values of heterogeneity were greater than 0.05 without evidence of heterogeneity, the fixed-effect IVW approach was considered; conversely, the random effects IVW approach was utilized if there was substantial heterogeneity (P < 0.05). The causal relationship between antioxidant vitamins and components of MetS was also analyzed. Additionally, in the casual association of dietary antioxidant vitamins with MetS, multivariate MR analysis was performed by adjustment for supplement antioxidant vitamins.

All statistical analyses were performed using R version 4.3.3 software. Statistical significance was determined by a two-sided *P* value < 0.05. Because of multiple comparisons, the significance level was corrected using the Bonferroni method. P value<0.008(0.05/6) was considered a strong association, a *P* value between 0.008 and 0.05 was considered a potential association, and the *P*-values were two-sided.

## 3 Results

### 3.1 NHANES general population study

A total of 10,308 adults from the US with data on exposure (dietary and serum vitamins) and outcome (MetS) were eligible for analysis during the study period. The main characteristics of these participants are shown in **Table 1**. Of these participants, 2,613 (25.36%) met the diagnostic criteria for MetS. In comparison to the healthy controls, individuals with MetS were more likely to be older, have a lower education level and income, smoke, engage in no physical activity, and to have a history of ASCVD, CKD, liver condition, thyroid disease, and cancer. The dietary intake of antioxidant vitamins (vitamin A, vitamin C, vitamin E, carotene, α-carotene, and β-carotene) was significantly lower in the MetS group than that of the control group (**Supplementary Table S2**). With regard to serum levels of antioxidant vitamins, the distribution between the two groups was consistent with dietary intake, with the exception of vitamins A and E, which exhibited opposite results. (**Supplementary Table S2**).

**Table 1:** Baseline characteristics of the participants in NHANES.

Characteristic	Total (n=10308)	Control (n=7695)	Metabolic syndrome (n=2613)	*P*
Age (years)				< 0.001
18-39	3330(32.31)	2901(42.47)	429(20.73)	
40-59	3390(32.89)	2443(38.93)	947(47.14)	
60-85	3588(34.81)	2351(18.60)	1237(32.12)	
Sex				0.172
Female	4996(48.47)	3617(50.00)	1379(52.10)	
Male	5312(51.53)	4078(50.00)	1234(47.90)	
Race				0.051
Non-Hispanic white	5569(54.03)	4100(73.31)	1469(76.91)	
Non-Hispanic black	1986(19.27)	1565(10.53)	421(8.07)	
Mexican American	2057(19.96)	1488(7.32)	569(6.84)	
Other Hispanic	340(3.30)	261(4.09)	79(4.18)	
Other race	356(3.45)	281(4.75)	75(4.01)	
Education				< 0.001
Less than high school	2815(27.31)	1993(15.67)	822(20.09)	
High school graduate	2521(24.46)	1802(24.45)	719(30.05)	
Some college	2868(27.82)	2168(31.11)	700(31.49)	
College graduate or above	2104(20.41)	1732(28.77)	372(18.37)	
Marital status				0.174
No	4483(43.49)	3382(40.95)	1101(38.72)	
Yes	5825(56.51)	4313(59.05)	1512(61.28)	
PIR				0.004
Low income	1660(16.1)	1194(11.06)	466(12.38)	
Middle income	2182(21.17)	1587(15.27)	595(17.75)	
High income	6466(62.73)	4914(73.66)	1552(69.87)	
Health insurance				0.003
No	2016(19.56)	1601(18.28)	415(14.66)	
Yes	8292(80.44)	6094(81.72)	2198(85.34)	
Smoking				< 0.001
Never	5148(49.94)	3928(50.69)	1220(46.71)	
Former	2794(27.11)	1946(23.99)	848(30.04)	
Now	2366(22.95)	1821(25.32)	545(23.25)	
Alcohol				< 0.001
No	3059(29.68)	2093(23.59)	966(34.51)	
Yes	7249(70.32)	5602(76.41)	1647(65.49)	
Physical activity				< 0.001
No	4032(39.12)	2794(29.42)	1238(42.06)	
Moderate	3133(30.39)	2273(30.75)	860(34.67)	
Vigorous	3143(30.49)	2628(39.83)	515(23.27)	
ASCVD				< 0.001
No	9211(89.36)	7065(94.32)	2146(85.81)	
Yes	1097(10.64)	630(5.68)	467(14.19)	
CKD				< 0.001
No	8371(81.21)	6546(89.73)	1825(76.75)	
Yes	1937(18.79)	1149(10.27)	788(23.25)	
Liver condition				0.020
No	9969(96.71)	7464(96.91)	2505(95.76)	
Yes	339(3.29)	231(3.09)	108(4.24)	
Thyroid disease				< 0.001
No	9324(90.45)	7059(91.10)	2265(86.63)	
Yes	984(9.55)	636(8.90)	348(13.37)	
Cancer				< 0.001
No	9358(90.78)	7042(92.13)	2316(89.26)	
Yes	950(9.22)	653(7.87)	297(10.74)	

PIR, poverty-income ratio; ASCVD, arteriosclerotic cardiovascular disease; CKD, chronic kidney disease.

**Figure 2:**
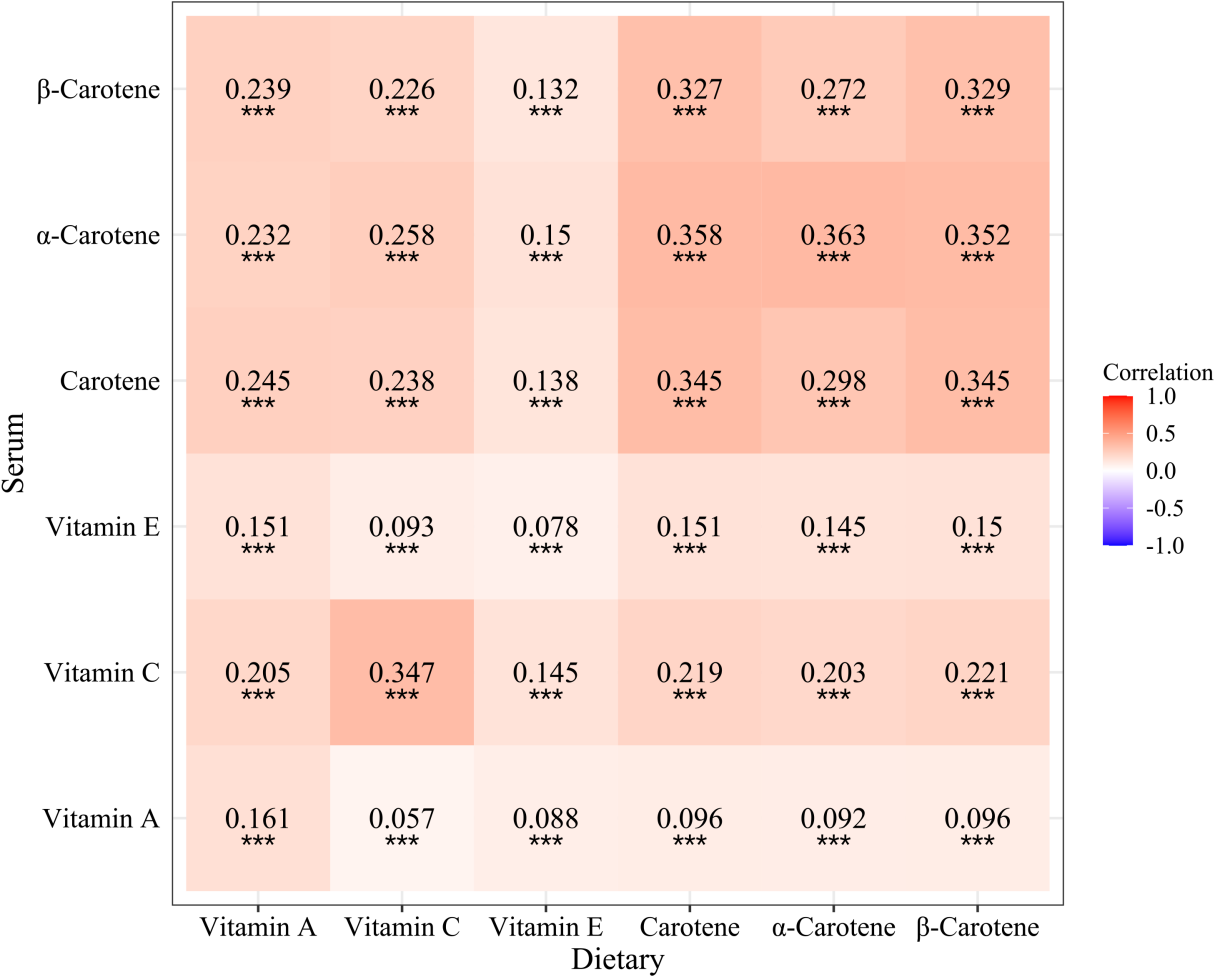
Correlation analysis of dietary and serum antioxidant vitamins. ^***^ *P*<0.001.

Correlation analysis revealed that dietary vitamin C (*r*=0.347, *P*<0.001), carotene (*r*=0.345, *P*<0.001), α-carotene (*r*=0.363, *P*<0.001), and β-carotene (*r*=0.329, *P*<0.001) were moderately correlated with serum levels, while vitamin A (*r*=0.161, *P*<0.001) and vitamin E (*r*=0.078, *P*<0.001) exhibited relatively low correlation with serum levels (**Figure 2**).

### 3.2 Associations between antioxidant vitamins and metabolic syndrome in NHANES

**Figure 3:**
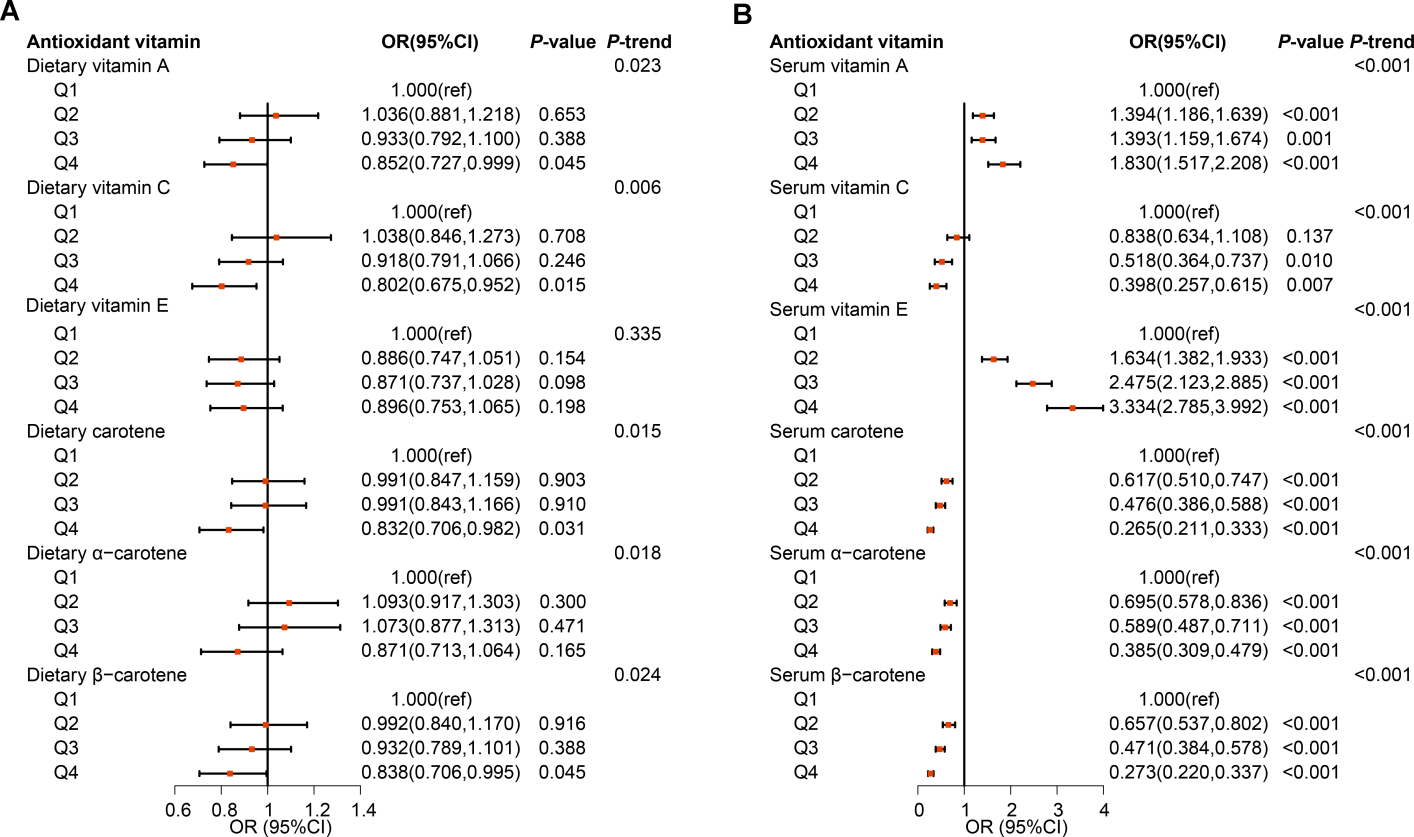
Observational associations of dietary **(A)** and serum **(B)** antioxidant vitamins on metabolic syndrome in NHANES population. The models were adjusted for demographic characteristics (age, gender, race, education, marital status, poverty-income ratio, and health insurance), lifestyle factors (smoking status, drinking condition, and physical activity), and health conditions (arteriosclerotic cardiovascular disease, chronic kidney disease, liver condition, thyroid disease, and cancer).

**Figure 4:**
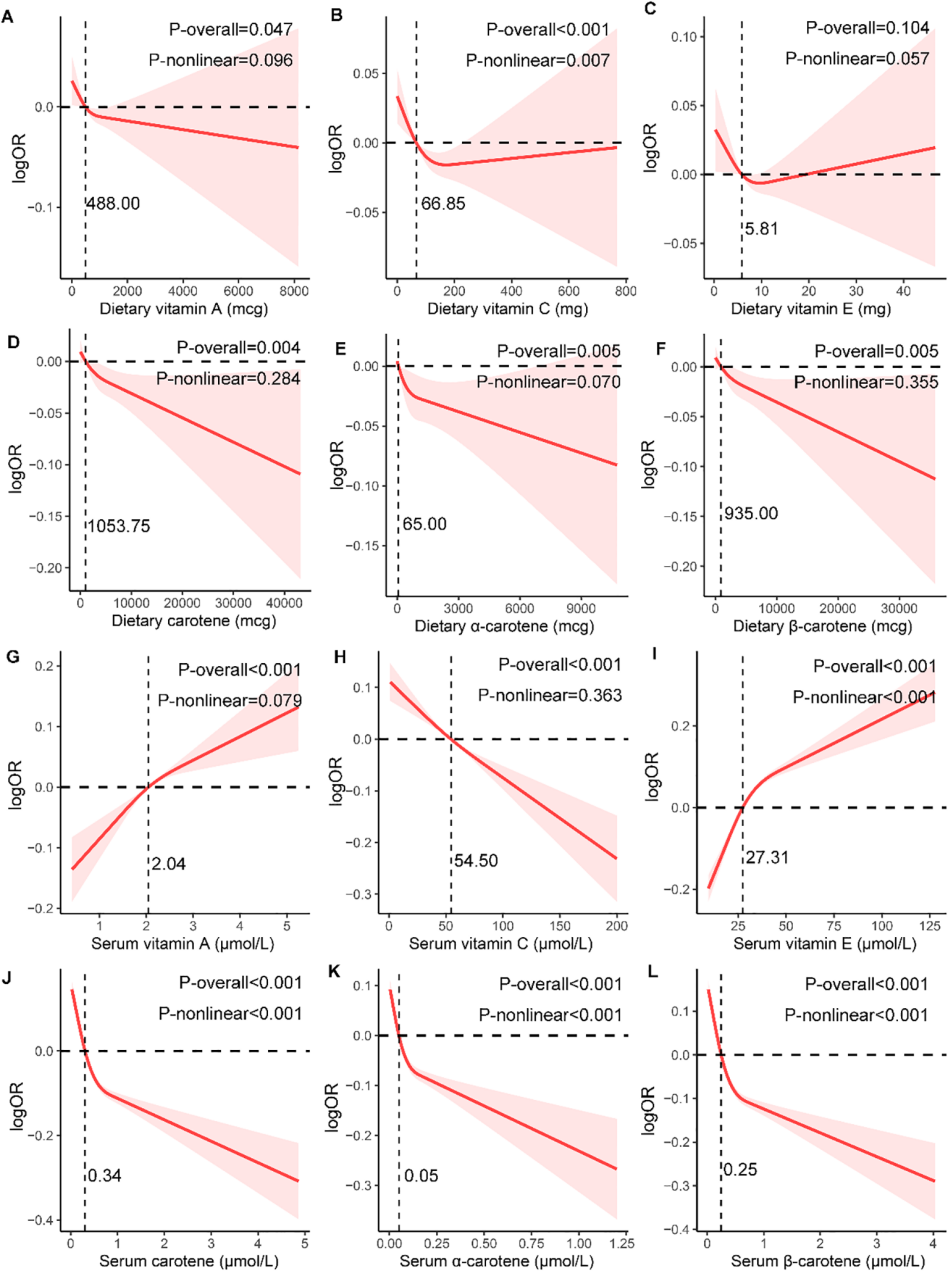
Dose-response association of dietary **(A–F)** and serum **(G–L)** antioxidant vitamins on metabolic syndrome in NHANES population. The models were adjusted for demographic characteristics (age, gender, race, education, marital status, poverty-income ratio, and health insurance), lifestyle factors (smoking status, drinking condition, and physical activity), and health conditions (arteriosclerotic cardiovascular disease, chronic kidney disease, liver condition, thyroid disease, and cancer).

The results of the weighted logistic regression indicated that dietary intake of vitamin A (OR=0.852, 95%CI: 0.727-0.999), vitamin C(OR=0.802, 95%CI: 0.675-0.952), carotene(OR=0.832, 95%CI: 0.706-0.982), and β-carotene(OR=0.838, 95%CI: 0.706-0.995) in quartile 4 had lower incidence of MetS compared to quartile 1, after adjustment for demographic characteristics, lifestyle factors, and health conditions (**Figure 3A**; **Supplementary Table S3**). The intake of four antioxidant vitamins was found to be significantly and inversely associated with lower risk of MetS (*P* for trend=0.023, 0.006, 0.015, 0.024, respectively). Moreover, the results of the restricted cubic spline (RCS) model indicated a dose-response relationship between dietary vitamin A (*P*-nonlinear=0.096), carotene (*P*-nonlinear=0.284), and β-carotene (*P*-nonlinear=0.355) and MetS (**Figure 4**). Furthermore, the relationship between dietary vitamin C and MetS was nonlinear (*P*-nonlinear=0.007), exhibiting a rapid decrease and then a gradual, stable in the risk of MetS as dietary vitamin A increase. Nevertheless, there were no significant associations between vitamin E and α-carotene intake with the risk of MetS across all the quartile categories.

For each component of MetS, the association of dietary antioxidant vitamins with HDL and obesity was mainly found (**Supplementary Figures S3-S7**; **Supplementary Tables S4-S8**). The intake of vitamin A (OR=0.808, 95%CI: 0.699-0.934), vitamin E (OR=0.787, 95%CI: 0.668-0.926), carotene (OR=0.806, 95%CI: 0.677-0.959), and β-carotene (OR=0.793, 95%CI: 0.659-0.954) in quartile 4, and vitamin C (OR=0.850, 95%CI: 0.724-0.998) in quartile 3 were inversely associated with the risk of HDL decreasing compared to quartile 1 (**Supplementary Figure S4**; **Supplementary Table S5**). Meanwhile, the higher the dietary intake of vitamin A (OR=0.847, 95%CI: 0.735-0.976), vitamin C (OR=0.777, 95%CI: 0.653-0.925), carotene (OR=0.719, 95%CI: 0.606-0.853), α-carotene (OR=0.858, 95%CI: 0.743-0.991), and β-carotene (OR=0.741, 95%CI: 0.622-0.882), the lower the risk of obesity (**Supplementary Figure S7**; **Supplementary Table S9**). In addition, dietary vitamin C in quartile 4 decreased the risk of blood glucose elevating (OR=0.837, 95%CI: 0.703-0.996), and dietary vitamin A in quartile 4 decreased the risk of blood pressure elevating (OR=0.820, 95%CI: 0.686-0.981).

The serum vitamin C, carotene, α-carotene and β-carotene in quartile 2 to 4 also present inversely associated with the risk of MetS when compared to quartile 1 with all *P* for trend <0.001 (**Figure 3B**; **Supplementary Table S3**). Furthermore, the relationship between vitamin C and MetS was linear (P-nonlinear=0.363), while the relationship between carotenes and MetS was non-linear (all P-nonlinear<0.001) with a rapid decrease and then slow decrease in the risk of MetS as serum carotenes increase (**Figure 4**). However, the higher level of serum vitamin A and vitamin E both increased the risk of MetS. For each component analysis, serum vitamin C and carotenes were also demonstrated to significantly reduce the risk of components of MetS (**Supplementary Figures S3-S7**; **Supplementary Tables S4-S9**).

### 3.3 Causality between antioxidant vitamins and metabolic syndrome in MR

**Figure 5:**
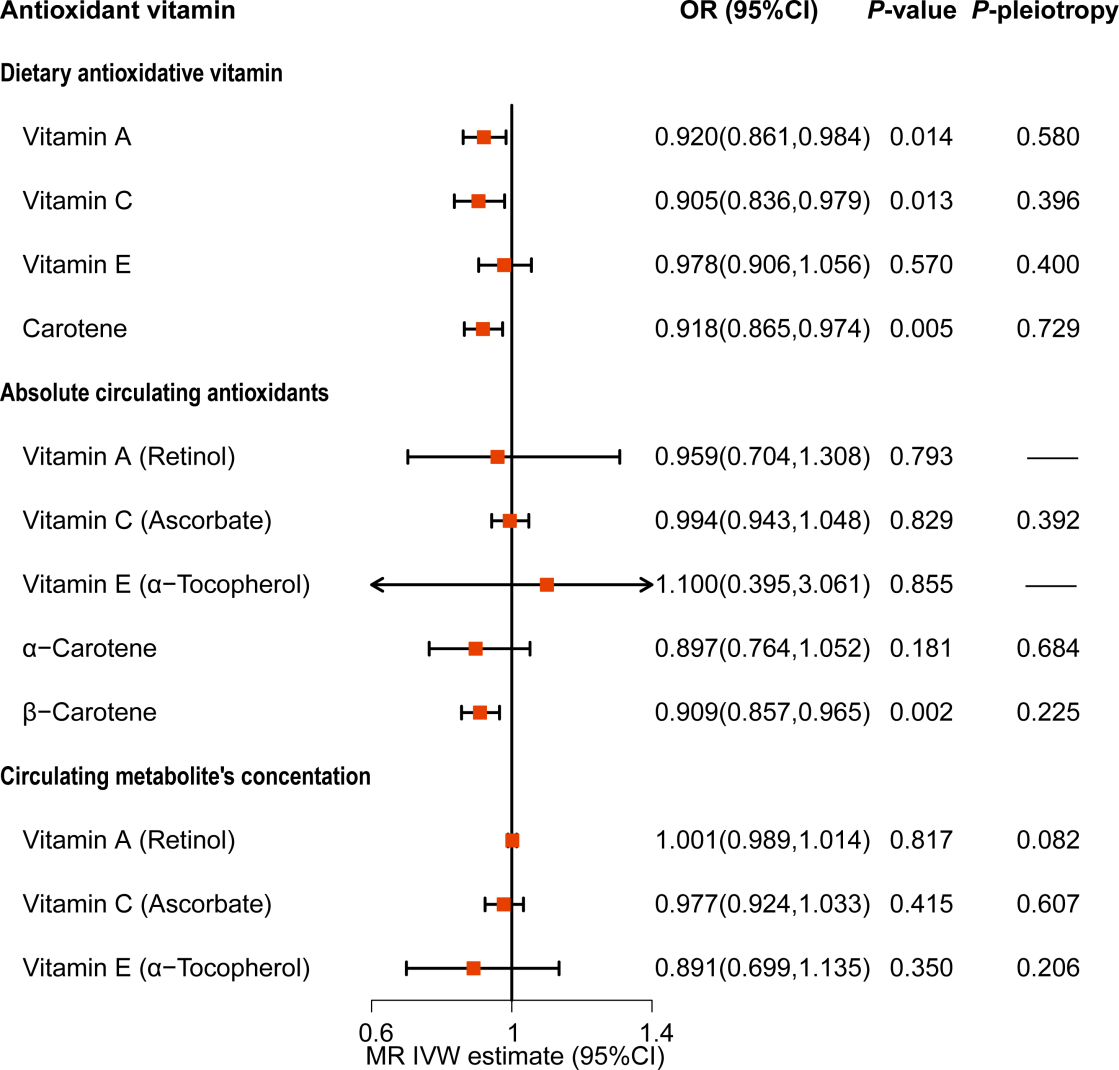
Causality association of dietary and serum antioxidant vitamins on metabolic syndrome in MR analysis.

**Figure 6:**
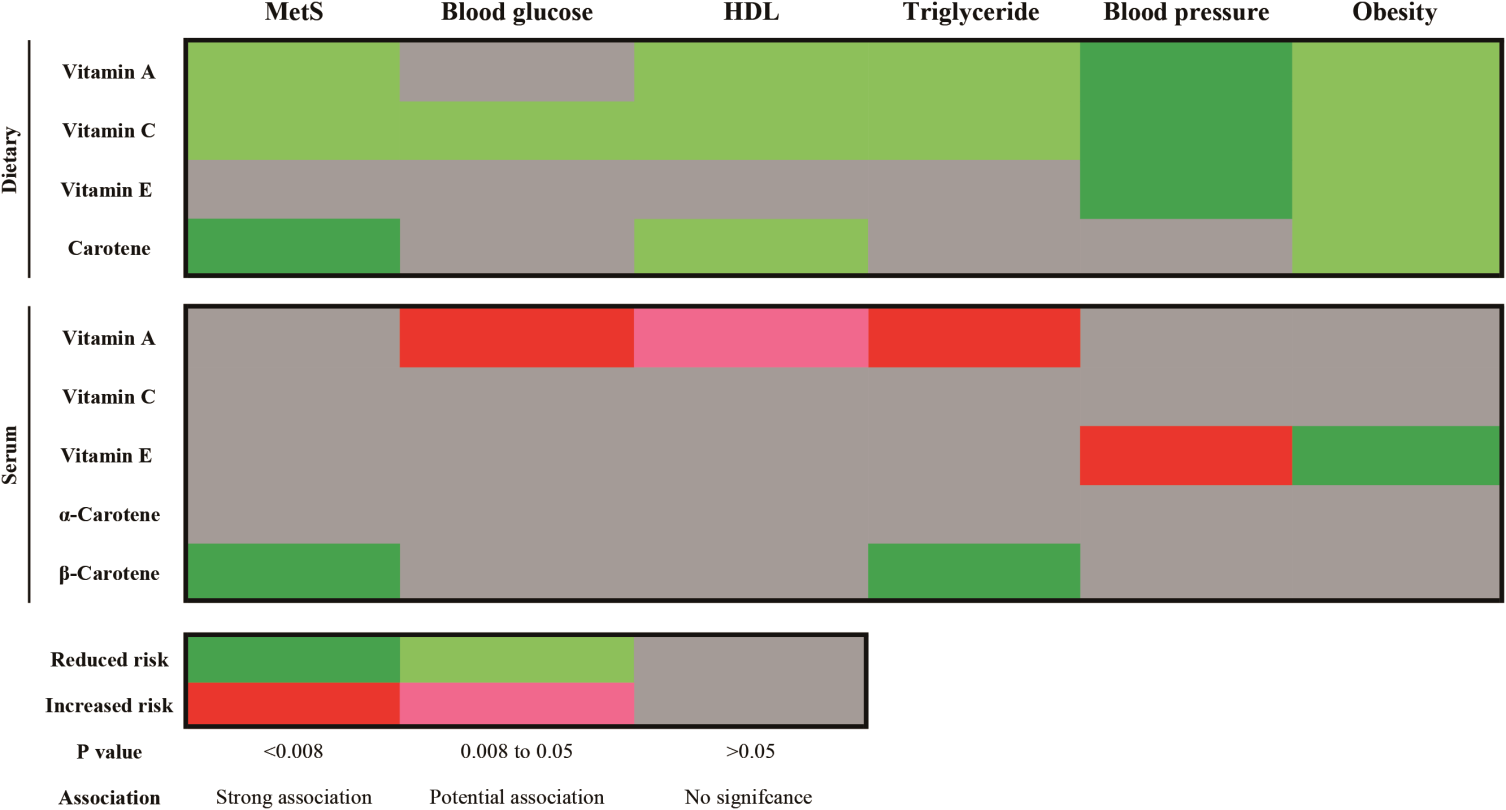
Summary results of Causality association of dietary and serum antioxidant vitamins on metabolic syndrome and its components in MR analysis. MetS, metabolic syndrome; HDL, high density lipoprotein.

The results showed that the MR effect estimates of dietary vitamin A (OR=0.920, 95%CI: 0.861-0.984), vitamin C (OR=0.905, 95%CI: 0.836-0.979) and carotene (OR=0.918, 95%CI: 0.865-0.974) were significantly associated with the risk of MetS (**Figure 5**; **Supplementary Table S9**). While, MR analyses of the dietary vitamin E (OR=0.978, 95%CI: 0.906-1.056) instrument did not show evidence for associations with MetS. Each antioxidant vitamins have different degree of causal association with each components of MetS, vitamin C, vitamin A, carotene and vitamin E reduced the 5, 4, 2, and 2 components risks, respectively (**Figure 6**; **Supplementary Figures S8-S12**; **Supplementary Tables S10-S14**). The MR-Egger intercept tests showed that for all *P*-pleiotropy >0.05, suggesting that there was no horizontal pleiotropy.

To avoid the effect of antioxidant vitamin supplements on MetS, we performed a multivariate MR. After adjusting for antioxidant vitamin supplements, dietary vitamin A (OR=0.912, 95%CI: 0.845-0.984) and vitamin C (OR=0.906, 95%CI: 0.829-0.989) were still found to reduce the risk of MetS (**Supplementary Table S15**).

For the serum antioxidant vitamins, there was only significance of the MR effect estimates between absolute circulating antioxidants β-carotene (OR=0.909, 95%CI: 0.857-0.965) and MetS (**Figure 5**; **Supplementary Table S9**). What’s more, β-carotene mainly decreased the level of triglyceride (OR=0.977, 95%CI: 0.961-0.922) (**Supplementary Figure S10**; **Supplementary Table S12**). We did not find causal relationships between other serum antioxidant vitamins and MetS. Nonetheless, genetically predicted circulating vitamin A decreased level of HDL (OR=0.911, 95%CI: 0.984-0.998) and increased the levels of glucose (OR=1.004, 95%CI: 1.001-1.006) and triglyceride (OR=1.012, 95%CI: 1.006-1.019) (**Figure 6**; **Supplementary Figures S9, S10**; **Supplementary Tables S11, S12**). Besides, absolute circulating vitamin E was associated with the increased risk of hypertension (OR=1.059, 95%CI: 1.028-1.090) and decreased risk of obesity. (OR=0.936, 95%CI: 0.894-0.979) (**Figure 6**; **Supplementary Figures S11, S12**; **Supplementary Tables S13, S14**).

## 4 Discussion

Our results showed that dietary vitamin A, vitamin C and carotenoids were causally associated with a reduced risk of MetS, whereas no association was found between dietary vitamin E intake and MetS. In the circulation, only an elevated β-carotenoid concentration was associated with a reduced risk of MetS. In addition, although circulating vitamin A and vitamin E levels did not show a causal relationship with MetS, their elevation may increase the risk of its components. This is evidenced by the fact that circulating vitamin A decreases HDL levels and increases glucose and triglyceride levels, while circulating vitamin E increases the risk of hypertension. Our study is an extension of Li et al. (21), and is also consistent with them. In their study, 18 circulating antioxidants nutrients, including vitamin A, C E, β-carotenoids and so on, were analyzed in relation to the MetS, and the protective effect of β-carotenoids was also found. But in this study, only serum antioxidant vitamins were studied, no dietary antioxidant vitamins. It is our understanding that our study is the inaugural investigation to provide a comprehensive analysis of the correlation between dietary and serum antioxidant vitamins and MetS. This analysis is based on a combination of large-scale observational study data and MR analysis of large-scale genetic data.

This study shows that dietary vitamin C is beneficial in reducing the risk of all components of the MetS, which is consistent with previous studies (34). Vitamin C, a widely used free radical scavenger, is a water-soluble vitamin that is completely dependent on dietary intake (35). Previous epidemiological studies have shown that vitamin C deficiency increases the risk of MetS in adults and leads to an increased incidence of diabetes (36). Vitamin C deficiency not only reduces cholesterol excretion and impairs hepatic lipid homeostasis, but also reduces the expression and activity of various antioxidant enzymes and increases markers of oxidative stress, thereby impairing the protective effects of hepatic protein and lipid oxidation (37). As a result of impaired gut barrier function due to overnutrition, MetS can repeatedly develop metabolic endotoxaemia, a vicious cycle that reduces vitamin C absorption while increasing inflammation and oxidative damage (38). A recent RCT showed that a micronutrient supplement high in vitamin C did not significantly reduce inflammation in patients with MetS, but did improve metabolic health indices (39). Currently, there is evidence that dietary vitamin C can improve obesity and diabetes by improving insulin sensitivity and myxophage abundance, regulating glucose and lipid metabolism (40–42); It may also help maintain vascular elasticity and promote healthy blood pressure levels by increasing the ability to synthesize NO and improving endothelial function (43).

The results of our study indicate that dietary vitamin A and carotenoids reduce the risk of MetS, and a beneficial effect of β-carotenoids on MetS was also found in serum. Beydoun et al. (44) also highlighted a negative association between β-carotenoids and MetS. Vitamin A is a fat-soluble vitamin that encompasses all compounds with the biological activity of retinol, including formed vitamin A (retinol) and pro-vitamin A (carotenoids) (45). β-carotene is a vital precursor of vitamin A, which can produce retinol after metabolism in the body and has significant biological activity of vitamin A. The antioxidant activity of vitamins A and β-carotene is conferred by the hydrophobic chain of the polyene unit, which can quench singlet oxygen, neutralize sulphur radicals and bind to and stabilize peroxy radicals (46). Vitamin A and its metabolite retinoic acid are thought to be important in maintaining normal white adipose tissue (WAT) and brown adipose tissue (BAT) physiology, and studies have shown that retinoic acid may contribute to the transition from WAT to BAT, thereby having a beneficial effect in preventing the accumulation of excess triglycerides (47). Carotenoids may also play an important role in adipose tissue biology, regulating adipocyte physiology by inhibiting peroxisome proliferation-activated receptors (PPAR), influencing the distribution of central obesity and the development of insulin resistance (48, 49). In addition, both vitamin A and β-carotenoids have been shown to play a protective role in blood pressure by modulating inflammatory responses and regulating NO pathways (50–52).

However, circulating vitamin A increased the risk of hyperglycemia and dyslipidemia in MR analysis, which was the opposite of the effect of dietary vitamin A. Our study also found a weak correlation between dietary vitamin A and serum levels, suggesting that circulating vitamin A does not represent dietary intake. The reason for the inconsistent effects of dietary and circulation vitamin A on metabolic indexes may be related to its absorption and transportation. Vitamin A is a fat-soluble vitamin that must be absorbed into the human body with lipids, so its biological function is closely related to lipid synthesis and metabolism, in addition to its antioxidant effect (47). Dietary vitamin A is decomposed into free retinol by the pancreatic fluid in the intestine or retinoesterase in the brush margin of villi, which is absorbed, combined with chylomicrons and transported to the liver by the lymphatic system, and stored in the form of fat droplets in the fat cells (53). Study shows increased vitamin A absorption in people with MetS compared to healthy adults (54). This increase may be due to increased activity of phospholipase B, pancreatic lipase-related protein 2, and phospholipase A2 group IB (54). Therefore, it is more likely that people with abnormal lipid metabolism in MetS will absorb more vitamin A than healthy people, resulting in increased blood concentrations, than that circulation vitamin A increased metabolic markers. Regarding the transportation of vitamin A, it enters the circulation when retinol binds to retinol-binding protein 4, which plays an important role in insulin resistance, dyslipidaemia, obesity and diabetes (55–57). Consequently, dietary intake of the antioxidant vitamin A is a means of reducing the risk of MetS, rather than circulating vitamin A.

Dietary vitamin E did not show an association or causality with reduced risk of MetS, possibly because the above three antioxidant vitamins reduced the risk of more than three components, whereas vitamin E reduced the risk of only two components, blood pressure and obesity. A meta-analysis of observational studies showed an inverse association between dietary vitamin E levels and MetS (58). Vitamin E is a potent peroxy radical scavenger that can block the propagation of free radicals in cell membranes and plasma lipoproteins, thereby reducing oxidative stress and lowering the risk of hypertension (59). It also increases the production of the vasodilators prostaglandins I2 and E2 in a dose-dependent manner by increasing the expression of intracellular phospholipase A2 and the release of the substrate AA, as well as inhibiting cyclooxygenase activity, thereby maintaining endothelial function (60). In addition, vitamin E and its metabolites regulate blood pressure by inhibiting diuretic potassium and calcium channels (61). At the same time, vitamin E may reduce adipose tissue fibrosis and collagen deposition through anti-inflammation and oxidative stress, and improve obesity and metabolic status (including hepatic steatosis, hypertriglyceridemia and insulin sensitivity) (62).

**Figure 7:**
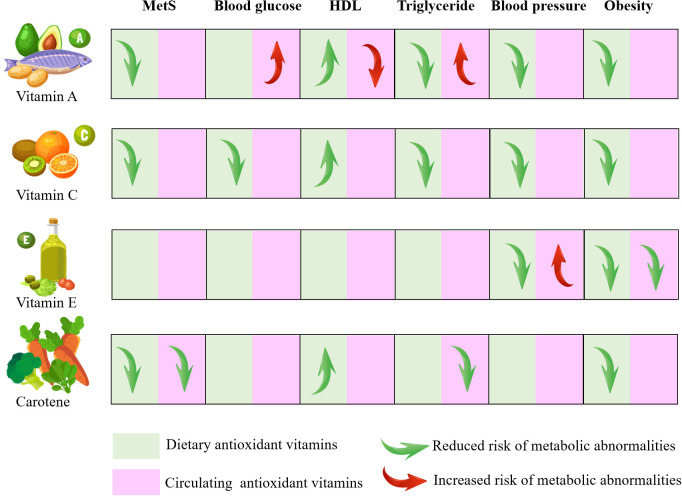
Visual model of the dietary recommendations and the risk of metabolic syndrome MetS, metabolic syndrome; HDL, high density lipoprotein.

The findings of our research have significant implications for public health and practical applications. Firstly, we elucidated the relationship between antioxidant vitamins and MetS. The risk of MetS can be reduced by dietary intake of antioxidant vitamins, especially vitamins A, C and carotenoids, which are beneficial for many components (**Figure 7**). Furthermore, we conducted a separate analysis of the associations between dietary and serum antioxidant vitamins with MetS, which revealed partial inconsistent associations, mainly for vitamin A. This is because the absorption and transport of fat-soluble vitamin are affected by lipid metabolism, so the serum concentrations of vitamin A do not absolutely reflect dietary intake. Individuals with abnormally elevated serum vitamins A should be mindful of the potential for metabolic abnormalities. Therefore, we recommend reducing MetS risk by increasing dietary intake rather than circulating concentration. Additional, Individuals with abnormal lipid metabolism should exercise caution when consuming vitamin A. It is recommended to monitor vitamin A levels through blood tests to prevent it accumulation that may lead to adverse effects. Before starting vitamin A supplementation, they should first manage their dyslipidemia with medication or other appropriate methods to ensure metabolic balance.

The major strength of our study was the use of MR analyses combined with the observational study design in NHANES. In a nationally representative sample of NHANES, the strategy of weighting algorithms and full adjustment for confounders was used to analyze the association between antioxidant vitamins and MetS. The consistency of findings between the MR analyses and the observational study made the results more robust. There are also several limitations to this study. First, antioxidant vitamin intake was measured by 24-hour DR, which may be subject to recall bias due to self-report. Second, due to the lack of data on vitamin supplementation in NHANES, this study did not analyze the effect of antioxidant vitamin supplementation on MetS. Third, in the MR study, we cannot completely rule out horizontal pleiotropy, an association between the outcome of interest and the MR instrument through pathways other than the proposed exposure, although the MR Egger intercepts in the statistical analysis showed no evidence of pleiotropy. Fourth, in order to obtain sufficient SNPs as instrumental variables, we relaxed the P-value setting of the association between exposure and SNPs. Although F-statistics were used to assess the instrumental variables, the problem of weak instrumental variable bias may still arise. Fifth, our observational data suggest a possible non-linear association between some antioxidant vitamins and MetS; however, only linear causality was examined in the MR study, so non-linear causality cannot be ruled out. Sixth, our observational and genetic data were not from the same sample, as we used a multiracial US population in the cross-sectional study and individuals of European descent in the MR study. Last, although we adjusted confounders sufficiently to reduce the effect of confounders and used MR analysis to simulate the environment of randomized controlled trial, control group was not truly set up to explore the effect of antioxidant vitamins on MetS. In the future, we will accurately quantify dietary antioxidant vitamin intake to carry out interventional studies, evaluate the relationship between dietary vitamin intake at different levels and MetS and the improvement of metabolic indicators, and further explore the mechanism of the difference in biological effects of dietary and circulating antioxidant vitamins.

## 5 Conclusions

Based on observational and MR studies, we find that adequate dietary intake of vitamins A, C and carotenoids may reduce the risk of MetS, whereas vitamin E only reduces two metabolic components. In the circulation, only increased β-carotenoid concentration is associated with a reduced risk of MetS. In addition, circulating vitamin A was associated with the increased risk of hyperglycaemia and dyslipidaemia. This suggests that eating more fruits and vegetables rich in antioxidant vitamins may help reduce the risk of MetS, but be wary of abnormally high concentrations of fat-soluble vitamins, such as vitamin A. If a high concentration of vitamin A is detected, it is not necessarily the result of increased intake, but most likely an increase in concentration due to abnormal absorption and transport caused by metabolic abnormalities.

## Data Availability

The datasets presented in this study can be found in online repositories. The names of the repository/repositories and accession number(s) can be found below: The data were publicly available from NHANSE (https://www.cdc.gov/nchs/nhanes/). All data generated or analyzed during this study are included in this published article. The available summarized GWAS data for MR analysis were obtained from the IEU open GWAS project (https://gwas.mrcieu.ac.uk/), GWAS Catalog (https://www.ebi.ac.uk/gwas/), or references introduced in manuscript.
