# Supplementary material for: Association of dietary and circulating antioxidant vitamins with metabolic syndrome: an observational and Mendelian randomization study
Source: Front Endocrinol (Lausanne). 2024 Oct 14;15:1446719. doi: 10.3389/fendo.2024.1446719 (PMC11513263; doi:10.3389/fendo.2024.1446719)
Supplement: Supplementary file 1 [file DataSheet1.docx]

**Content**

[Table S1 Detailed information regarding studies and datasets used in MR study 3](#_Toc9094)

[Table S2 Dietary intake and serum level of antioxidant vitamins in NHANES population 4](#_Toc26865)

[Table S3 Odds ratio (95% CI) for metabolic syndrome according to dietary and serum antioxidant vitamins in NHANES population 7](#_Toc24348)

[Table S4 Odds ratio (95% CI) for blood glucose elevating according to dietary and serum antioxidant vitamins in NHANES population 11](#_Toc32204)

[Table S5 Odds ratio (95% CI) for HDL decreasing according to dietary and serum antioxidant vitamins in NHANES population 15](#_Toc24792)

[Table S6 Odds ratio (95% CI) for triglyceride elevating according to dietary and serum antioxidant vitamins in NHANES population 20](#_Toc29260)

[Table S7 Odds ratio (95% CI) for blood pressure elevating according to dietary and serum antioxidant vitamins in NHANES population 24](#_Toc26201)

[Table S8 Odds ratio (95% CI) for obesity according to dietary and serum antioxidant vitamins in NHANES population 28](#_Toc18540)

[Table S9 Causal effect, heterogeneity, pleiotropy of dietary and serum antioxidant vitamins on metabolic syndrome in MR analysis 32](#_Toc306)

[Table S10 Causal effect, heterogeneity, pleiotropy of dietary and serum antioxidant vitamins on blood glucose in MR analysis 34](#_Toc11473)

[Table S11 Causal effect, heterogeneity, pleiotropy of dietary and serum antioxidant vitamins on HDL in MR analysis 37](#_Toc3924)

[Table S12 Causal effect, heterogeneity, pleiotropy of dietary and serum antioxidant vitamins on triglyceride in MR analysis 39](#_Toc16922)

[Table S13 Causal effect, heterogeneity, pleiotropy of dietary and serum antioxidant vitamins on hypertension in MR analysis 43](#_Toc15240)

[Table S14 Causal effect, heterogeneity, pleiotropy of dietary and serum antioxidant vitamins on obesity in MR analysis 45](#_Toc30705)

[Table S15 Multivariate MR for causality association of dietary antioxidant vitamins on metabolic syndrome and its components 48](#_Toc28362)

[Figure S1 Flow chart for the selection of participants in NHANES. 50](#_Toc21363)

[Figure S2 MR assumptions of this study 51](#_Toc8593)

[Figure S3 Observational associations of dietary (A) and serum (B) antioxidant vitamins on blood glucose elevating in NHANES population 52](#_Toc31704)

[Figure S4 Observational associations of dietary (A) and serum (B) antioxidant vitamins on HDL decreasing in NHANES population 54](#_Toc13385)

[Figure S5 Observational associations of dietary (A) and serum (B) antioxidant vitamins on triglyceride elevating in NHANES population 55](#_Toc13719)

[Figure S6 Observational associations of dietary (A) and serum (B) antioxidant vitamins on blood pressure elevating in NHANES population 56](#_Toc28796)

[Figure S7 Observational associations of dietary (A) and serum (B) antioxidant vitamins on obesity in NHANES population 58](#_Toc1870)

[Figure S8 Causality association of dietary and serum antioxidant vitamins on blood glucose in MR analysis 58](#_Toc8330)

[Figure S9 Causality association of dietary and serum antioxidant vitamins on HDL in MR analysis 59](#_Toc24368)

[Figure S10 Causality association of dietary and serum antioxidant vitamins on triglyceride in MR analysis 60](#_Toc23007)

[Figure S11 Causality association of dietary and serum antioxidant vitamins on hypertension in MR analysis 61](#_Toc1243)

[Figure S12 Causality association of dietary and serum antioxidant vitamins on obesity in MR analysis 62](#_Toc13824)

**Table S1 Detailed information regarding studies and datasets used in MR study**

| **[Trait](https://gwas.mrcieu.ac.uk/datasets/?gwas_id__icontains=&year__iexact=&trait__icontains=vitamin+a&consortium__icontains=&page=1&sort=-trait)** | | **[Year](https://gwas.mrcieu.ac.uk/datasets/?gwas_id__icontains=&year__iexact=&trait__icontains=vitamin+a&consortium__icontains=&page=1&sort=year)** | **Population** | **[Consortium](https://gwas.mrcieu.ac.uk/datasets/?gwas_id__icontains=&year__iexact=&trait__icontains=vitamin+a&consortium__icontains=&page=1&sort=consortium)** | **[Sample size](https://gwas.mrcieu.ac.uk/datasets/?gwas_id__icontains=&year__iexact=&trait__icontains=vitamin+a&consortium__icontains=&page=1&sort=sample_size)** | **[Number of SNPs](https://gwas.mrcieu.ac.uk/datasets/?gwas_id__icontains=&year__iexact=&trait__icontains=vitamin+a&consortium__icontains=&page=1&sort=nsnp)** | **Unit** | **[GWAS ID](https://gwas.mrcieu.ac.uk/datasets/?gwas_id__icontains=&year__iexact=&trait__icontains=vitamin+a&consortium__icontains=&page=1&sort=-gwas_id)** | **PMID** |
| --- | --- | --- | --- | --- | --- | --- | --- | --- | --- |
| **Dietary antioxidative vitamin** | |  |  |  |  |  |  |  |  |
|  | **Vitamin A** | 2018 | European | UK Biobank | 62,991 | 9,851,867 | SD | ukb-b-17406 | NA |
|  | **Vitamin C** | 2018 | European | UK Biobank | 64,979 | 9,851,867 | SD | ukb-b-19390 | NA |
|  | **Vitamin E** | 2018 | European | UK Biobank | 64,979 | 9,851,867 | SD | ukb-b-6888 | NA |
|  | **Carotene** | 2018 | European | UK Biobank | 64,979 | 9,851,867 | SD | ukb-b-16202 | NA |
| **Supplement antioxidative vitamin** | |  |  |  |  |  |  |  |  |
|  | **Vitamin A** | 2018 | European | UK Biobank | 460,351 | 9,851,867 | SD | ukb-b-9596 | NA |
|  | **Vitamin C** | 2018 | European | UK Biobank | 460,351 | 9,851,867 | SD | ukb-b-15175 | NA |
|  | **Vitamin E** | 2018 | European | UK Biobank | 460,351 | 9,851,867 | SD | ukb-b-12506 | NA |
| **Absolute circulating antioxidants** | |  |  |  |  |  |  |  |  |
|  | **Vitamin A (Retinol)** | 2011 | European | GWAS analysis | 5,006 | —— | µg/L in natural log-transformed scale | —— | 21878437 |
|  | **Vitamin C (Ascorbate)** | 2020 | European | Meta-analysis | 52,018 | —— | μmol/l | —— | 33203707 |
|  | **Vitamin E (α-Tocopherol)** | 2011 | European | GWAS analysis | 4,014 | —— | mg/L in log-transformed scale | —— | 21729881 |
|  | **α-Carotene** | 2012 | European | GWAS analysis | 2,344 | —— | µg/L in natural log-transformed scale | —— | 23134893 |
|  | **β-Carotene** | 2012 | European | GWAS analysis | 2,344 | —— | µg/L in natural log-transformed scale | —— | 23134893 |
| **Circulating metabolite's concentation** | |  |  |  |  |  |  |  |  |
|  | **Vitamin A (Retinol)** | 2011 | European | GWAS analysis | 1,960 | —— | log10-transfomed metabolites concentration | —— | 28263315 |
|  | **Vitamin C (Ascorbate）** | 2014 | European | GWAS analysis | 2,085 | 2,545,101 | log10-transfomed metabolites concentration | met-a-348 | 24816252 |
|  | **Vitamin E (α-Tocopherol)** | 2014 | European | GWAS analysis | 7,725 | 2,545,636 | log10-transfomed metabolites concentration | met-a-340 | 24816252 |
| **Metabolic syndrome and its components** | | |  |  |  |  |  |  |  |
|  | **Metabolic syndrome** | 2019 | European | GWAS analysis | 291,107 | 9,463,307 | —— | —— | 31589552 |
|  | **Fasting blood glucose** | 2021 | European | Meta-analysis | 281,416 | 34,064,006 | —— | —— | 34059833 |
|  | **Waistline** | 2018 | European | UK Biobank | 462,166 | 9,851,867 | SD | ukb-b-9405 | NA |
|  | **Hypertension** | 2018 | European | UK Biobank | 463,010 | 9,851,867 | SD | ukb-b-12493 | NA |
|  | **Triglycerides** | 2020 | European | MRC-IEU | 441,016 | 12,321,875 | NA | ieu-b-111 | 32203549 |
|  | **High-density lipoprotein cholesterol** | 2020 | European | MRC-IEU | 403,943 | 12,321,875 | NA | ieu-b-109 | 32203549 |

**Table S2 Dietary intake and serum level of antioxidant vitamins in NHANES population**

| **Antioxidant vitamin** | **Total (n=10308)** | **Control (n=7695)** | **Metabolic syndrome (n=2613)** | ***P*** |
| --- | --- | --- | --- | --- |
| **Dietary vitamin A (mcg)** | 512.00[303.50, 794.50] | 519.00[306.00, 804.50] | 490.00[298.00, 749.50] | 0.009 |
| **Q1** | 2578(25.01) | 1906(22.41) | 672(23.05) | 0.043 |
| **Q2** | 2585(25.08) | 1874(24.04) | 711(26.58) |  |
| **Q3** | 2568(24.91) | 1923(25.77) | 645(26.24) |  |
| **Q4** | 2577(25.00) | 1992(27.79) | 585(24.13) |  |
| **Dietary vitamin C (mg)** | 63.30[29.60, 120.05] | 65.55[30.10, 124.10] | 56.40[28.40, 106.75] | <0.001 |
| **Q1** | 2583(25.06) | 1896(25.54) | 687(27.80) | <0.001 |
| **Q2** | 2571(24.94) | 1893(25.52) | 678(28.30) |  |
| **Q3** | 2578(25.01) | 1909(24.24) | 669(24.37) |  |
| **Q4** | 2576(24.99) | 1997(24.70) | 579(19.53) |  |
| **Dietary vitamin E (mg)** | 6.16[4.19, 8.90] | 6.25[4.29, 9.02] | 5.82[3.92, 8.55] | <0.001 |
| **Q1** | 2584(25.07) | 1821(20.67) | 763(25.34) | <0.001 |
| **Q2** | 2574(24.97) | 1907(24.13) | 667(24.57) |  |
| **Q3** | 2577(25) | 1973(26.41) | 604(24.30) |  |
| **Q4** | 2573(24.96) | 1994(28.79) | 579(25.79) |  |
| **Dietary carotene (mcg)** | 1077.00[425.50, 2991.00] | 1087.50[431.50, 3113.00] | 1050.00[408.00, 2745.00] | 0.054 |
| **Q1** | 2579(25.02) | 1909(23.67) | 670(24.87) | 0.072 |
| **Q2** | 2575(24.98) | 1924(25.38) | 651(25.26) |  |
| **Q3** | 2577(25.00) | 1893(25.32) | 684(26.94) |  |
| **Q4** | 2577(25.00) | 1969(25.63) | 608(22.93) |  |
| **Dietary α-carotene (mcg)** | 64.50[21.00, 374.50] | 65.00[21.00, 399.50] | 64.00[21.00, 316.00] | 0.307 |
| **Q1** | 2610(25.32) | 1964(25.60) | 646(25.21) | 0.082 |
| **Q2** | 2567(24.90) | 1895(24.69) | 672(25.50) |  |
| **Q3** | 2554(24.78) | 1866(23.68) | 688(26.06) |  |
| **Q4** | 2577(25.00) | 1970(26.03) | 607(23.24) |  |
| **Dietary β-carotene (mcg)** | 957.00[391.00, 2520.00] | 967.50[394.50, 2586.00] | 908.00[369.00, 2327.50] | 0.043 |
| **Q1** | 2581(25.04) | 1904(23.54) | 677(25.07) | 0.159 |
| **Q2** | 2574(24.97) | 1920(25.21) | 654(25.57) |  |
| **Q3** | 2577(25.00) | 1910(25.45) | 667(25.96) |  |
| **Q4** | 2576(24.99) | 1961(25.80) | 615(23.40) |  |
| **Serum vitamin A (μmol/L)** | 2.08[1.73, 2.48] | 2.05[1.70, 2.43] | 2.18[1.84, 2.62] | <0.001 |
| **Q1** | 2577(25.00) | 2081(24.02) | 496(16.38) | <0.001 |
| **Q2** | 2577(25.00) | 1961(25.65) | 616(24.42) |  |
| **Q3** | 2589(25.12) | 1954(26.27) | 635(25.56) |  |
| **Q4** | 2565(24.88) | 1699(24.06) | 866(33.64) |  |
| **Serum vitamin C (μmol/L) ^a^** | 54.50[33.50, 70.40] | 56.80[36.30, 72.10] | 47.10[26.10, 64.20] | <0.001 |
| **Q1** | 1673(25.23) | 1177(23.68) | 496(35.28) | <0.001 |
| **Q2** | 1670(25.19) | 1251(22.84) | 419(26.69) |  |
| **Q3** | 1661(25.05) | 1323(26.51) | 338(20.07) |  |
| **Q4** | 1626(24.52) | 1329(26.97) | 297(17.96) |  |
| **Serum vitamin E (μmol/L)** | 27.35[21.73, 35.53] | 26.15[20.99, 33.44] | 31.17[24.85, 41.80] | <0.001 |
| **Q1** | 2578(25.01) | 2228(28.22) | 350(12.86) | <0.001 |
| **Q2** | 2576(24.99) | 2028(26.67) | 548(20.81) |  |
| **Q3** | 2581(25.04) | 1835(24.89) | 746(29.48) |  |
| **Q4** | 2573(24.96) | 1604(20.22) | 969(36.85) |  |
| **Serum carotene (μmol/L)** | 0.29[0.17, 0.53] | 0.31[0.18, 0.57] | 0.24[0.14, 0.40] | <0.001 |
| **Q1** | 2578(25.01) | 1773(23.62) | 805(35.37) | <0.001 |
| **Q2** | 2584(25.07) | 1882(25.32) | 702(26.96) |  |
| **Q3** | 2570(24.93) | 1935(24.06) | 635(22.53) |  |
| **Q4** | 2576(24.99) | 2105(26.99) | 471(15.13) |  |
| **Serum α-carotene (μmol/L)** | 0.05[0.03, 0.10] | 0.05[0.03, 0.10] | 0.04[0.02, 0.08] | <0.001 |
| **Q1** | 2623(25.45) | 1851(23.84) | 772(33.03) | <0.001 |
| **Q2** | 2530(24.54) | 1829(24.13) | 701(26.02) |  |
| **Q3** | 2582(25.05) | 1917(24.60) | 665(23.95) |  |
| **Q4** | 2573(24.96) | 2098(27.43) | 475(16.99) |  |
| **Serum β-carotene (μmol/L)** | 0.23[0.14, 0.43] | 0.25[0.15, 0.46] | 0.19[0.11, 0.33] | <0.001 |
| **Q1** | 2578(25.01) | 1777(23.90) | 801(35.24) | <0.001 |
| **Q2** | 2607(25.29) | 1910(25.51) | 697(27.71) |  |
| **Q3** | 2549(24.73) | 1913(23.88) | 636(21.68) |  |
| **Q4** | 2574(24.97) | 2095(26.71) | 479(15.37) |  |

^a^ There was no information of serum vitamin C in 2001-2002 (n=6630)

**Table S3 Odds ratio (95% CI) for metabolic syndrome according to dietary and serum antioxidant vitamins in NHANES population**

| **Antioxidant vitamin** | **Crude Model** | |  | **Model 1** | |  | **Model 2** | |  | **Model 3** | | |
| --- | --- | --- | --- | --- | --- | --- | --- | --- | --- | --- | --- | --- |
|  | **OR (95%CI)** | ***P*-value** | **P- trend** | **OR (95%CI)** | ***P*-value** | **P- trend** | **OR (95%CI)** | ***P*-value** | ***P*-trend** | **OR (95%CI)** | ***P*-value** | **P- trend** |
| **Dietary vitamin A (mcg)** |  |  | 0.003 |  |  | 0.004 |  |  | 0.009 |  |  | 0.023 |
| **Q1** | 1.000(ref) |  |  | 1.000(ref) |  |  | 1.000(ref) |  |  | 1.000(ref) |  |  |
| **Q2** | 1.075(0.909,1.271) | 0.390 |  | 1.033(0.874,1.222) | 0.692 |  | 1.048(0.890,1.234) | 0.557 |  | 1.036(0.881,1.218) | 0.653 |  |
| **Q3** | 0.990(0.836,1.172) | 0.905 |  | 0.917(0.776,1.083) | 0.294 |  | 0.929(0.787,1.095) | 0.363 |  | 0.933(0.792,1.100) | 0.388 |  |
| **Q4** | 0.844(0.735,0.970) | 0.018 |  | 0.824(0.709,0.956) | 0.013 |  | 0.843(0.724,0.982) | 0.029 |  | 0.852(0.727,0.999) | 0.045 |  |
| **Dietary vitamin C (mg)** |  |  | <0.001 |  |  | <0.001 |  |  | 0.003 |  |  | 0.006 |
| **Q1** | 1.000(ref) |  |  | 1.000(ref) |  |  | 1.000(ref) |  |  | 1.000(ref) |  |  |
| **Q2** | 1.019(0.854,1.216) | 0.830 |  | 1.013(0.835,1.229) | 0.890 |  | 1.038(0.849,1.271) | 0.703 |  | 1.038(0.846,1.273) | 0.708 |  |
| **Q3** | 0.923(0.810,1.053) | 0.227 |  | 0.895(0.777,1.030) | 0.118 |  | 0.919(0.793,1.065) | 0.249 |  | 0.918(0.791,1.066) | 0.246 |  |
| **Q4** | 0.726(0.625,0.844) | <0.001 |  | 0.763(0.651,0.895) | 0.002 |  | 0.800(0.678,0.943) | 0.010 |  | 0.802(0.675,0.952) | 0.015 |  |
| **Dietary vitamin E (mg)** |  |  | <0.001 |  |  | 0.091 |  |  | 0.166 |  |  | 0.335 |
| **Q1** | 1.000(ref) |  |  | 1.000(ref) |  |  | 1.000(ref) |  |  | 1.000(ref) |  |  |
| **Q2** | 0.831(0.716,0.964) | 0.016 |  | 0.853(0.726,1.002) | 0.053 |  | 0.867(0.733,1.026) | 0.092 |  | 0.886(0.747,1.051) | 0.154 |  |
| **Q3** | 0.751(0.642,0.877) | <0.001 |  | 0.817(0.699,0.955) | 0.013 |  | 0.838(0.715,0.983) | 0.031 |  | 0.871(0.737,1.028) | 0.098 |  |
| **Q4** | 0.731(0.623,0.856) | <0.001 |  | 0.840(0.713,0.989) | 0.037 |  | 0.861(0.726,1.022) | 0.084 |  | 0.896(0.753,1.065) | 0.198 |  |
| **Dietary carotene (mcg)** |  |  | 0.026 |  |  | 0.005 |  |  | 0.008 |  |  | 0.015 |
| **Q1** | 1.000(ref) |  |  | 1.000(ref) |  |  | 1.000(ref) |  |  | 1.000(ref) |  |  |
| **Q2** | 0.947(0.827,1.085) | 0.426 |  | 0.970(0.832,1.131) | 0.688 |  | 0.981(0.840,1.145) | 0.799 |  | 0.991(0.847,1.159) | 0.903 |  |
| **Q3** | 1.013(0.878,1.169) | 0.858 |  | 0.979(0.841,1.139) | 0.773 |  | 0.995(0.847,1.168) | 0.946 |  | 0.991(0.843,1.166) | 0.91 |  |
| **Q4** | 0.852(0.743,0.976) | 0.022 |  | 0.805(0.686,0.944) | 0.010 |  | 0.821(0.699,0.965) | 0.019 |  | 0.832(0.706,0.982) | 0.031 |  |
| **Dietary α-carotene (mcg)** |  |  | 0.047 |  |  | 0.006 |  |  | 0.010 |  |  | 0.018 |
| **Q1** | 1.000(ref) |  |  | 1.000(ref) |  |  | 1.000(ref) |  |  | 1.000(ref) |  |  |
| **Q2** | 1.049(0.898,1.224) | 0.539 |  | 1.057(0.897,1.246) | 0.494 |  | 1.081(0.914,1.278) | 0.346 |  | 1.093(0.917,1.303) | 0.3 |  |
| **Q3** | 1.117(0.927,1.346) | 0.236 |  | 1.040(0.858,1.262) | 0.677 |  | 1.064(0.872,1.299) | 0.525 |  | 1.073(0.877,1.313) | 0.471 |  |
| **Q4** | 0.907(0.763,1.077) | 0.259 |  | 0.837(0.695,1.007) | 0.058 |  | 0.855(0.705,1.037) | 0.107 |  | 0.871(0.713,1.064) | 0.165 |  |
| **Dietary β-carotene (mcg)** |  |  | 0.029 |  |  | 0.010 |  |  | 0.016 |  |  | 0.024 |
| **Q1** | 1.000(ref) |  |  | 1.000(ref) |  |  | 1.000(ref) |  |  | 1.000(ref) |  |  |
| **Q2** | 0.952(0.820,1.105) | 0.510 |  | 0.973(0.826,1.146) | 0.732 |  | 0.982(0.834,1.156) | 0.822 |  | 0.992(0.840,1.170) | 0.916 |  |
| **Q3** | 0.957(0.826,1.109) | 0.553 |  | 0.920(0.788,1.073) | 0.274 |  | 0.934(0.794,1.097) | 0.389 |  | 0.932(0.789,1.101) | 0.388 |  |
| **Q4** | 0.852(0.738,0.982) | 0.028 |  | 0.809(0.683,0.959) | 0.016 |  | 0.828(0.699,0.982) | 0.031 |  | 0.838(0.706,0.995) | 0.045 |  |
| **Serum vitamin A (μmol/L)** |  |  | <0.001 |  |  | <0.001 |  |  | <0.001 |  |  | <0.001 |
| **Q1** | 1.000(ref) |  |  | 1.000(ref) |  |  | 1.000(ref) |  |  | 1.000(ref) |  |  |
| **Q2** | 1.396(1.205,1.617) | <0.001 |  | 1.350(1.156,1.576) | <0.001 |  | 1.386(1.182,1.626) | <0.001 |  | 1.394(1.186,1.639) | <0.001 |  |
| **Q3** | 1.426(1.210,1.682) | <0.001 |  | 1.347(1.128,1.609) | 0.002 |  | 1.397(1.172,1.666) | <0.001 |  | 1.393(1.159,1.674) | 0.001 |  |
| **Q4** | 2.049(1.761,2.384) | <0.001 |  | 1.841(1.540,2.199) | <0.001 |  | 1.937(1.613,2.327) | <0.001 |  | 1.830(1.517,2.208) | <0.001 |  |
| **Serum vitamin C (μmol/L)** |  |  | <0.001 |  |  | <0.001 |  |  | <0.001 |  |  | <0.001 |
| **Q1** | 1.000(ref) |  |  | 1.000(ref) |  |  | 1.000(ref) |  |  | 1.000(ref) |  |  |
| **Q2** | 0.785(0.674,0.913) | 0.003 |  | 0.855(0.715,1.022) | 0.08 |  | 0.838(0.685,1.025) | 0.078 |  | 0.838(0.634,1.108) | 0.137 |  |
| **Q3** | 0.508(0.410,0.630) | <0.001 |  | 0.505(0.401,0.636) | <0.001 |  | 0.499(0.389,0.639) | <0.001 |  | 0.518(0.364,0.737) | 0.01 |  |
| **Q4** | 0.447(0.341,0.586) | <0.001 |  | 0.385(0.292,0.509) | <0.001 |  | 0.386(0.283,0.526) | <0.001 |  | 0.398(0.257,0.615) | 0.007 |  |
| **Serum vitamin E (μmol/L)** |  |  | <0.001 |  |  | <0.001 |  |  | <0.001 |  |  | <0.001 |
| **Q1** | 1.000(ref) |  |  | 1.000(ref) |  |  | 1.000(ref) |  |  | 1.000(ref) |  |  |
| **Q2** | 1.712(1.451,2.019) | <0.001 |  | 1.628(1.376,1.925) | <0.001 |  | 1.633(1.386,1.923) | <0.001 |  | 1.634(1.382,1.933) | <0.001 |  |
| **Q3** | 2.598(2.246,3.005) | <0.001 |  | 2.321(1.973,2.730) | <0.001 |  | 2.418(2.057,2.843) | <0.001 |  | 2.475(2.123,2.885) | <0.001 |  |
| **Q4** | 3.999(3.409,4.691) | <0.001 |  | 3.147(2.633,3.761) | <0.001 |  | 3.314(2.768,3.966) | <0.001 |  | 3.334(2.785,3.992) | <0.001 |  |
| **Serum carotene (μmol/L)** |  |  | <0.001 |  |  | <0.001 |  |  | <0.001 |  |  | <0.001 |
| **Q1** | 1.000(ref) |  |  | 1.000(ref) |  |  | 1.000(ref) |  |  | 1.000(ref) |  |  |
| **Q2** | 0.711(0.606,0.834) | <0.001 |  | 0.635(0.531,0.760) | <0.001 |  | 0.620(0.516,0.745) | <0.001 |  | 0.617(0.510,0.747) | <0.001 |  |
| **Q3** | 0.625(0.526,0.743) | <0.001 |  | 0.496(0.407,0.605) | <0.001 |  | 0.482(0.393,0.590) | <0.001 |  | 0.476(0.386,0.588) | <0.001 |  |
| **Q4** | 0.374(0.313,0.448) | <0.001 |  | 0.270(0.220,0.332) | <0.001 |  | 0.265(0.214,0.329) | <0.001 |  | 0.265(0.211,0.333) | <0.001 |  |
| **Serum α-carotene (μmol/L)** |  |  | <0.001 |  |  | <0.001 |  |  | <0.001 |  |  | <0.001 |
| **Q1** | 1.000(ref) |  |  | 1.000(ref) |  |  | 1.000(ref) |  |  | 1.000(ref) |  |  |
| **Q2** | 0.778(0.661,0.917) | 0.004 |  | 0.701(0.589,0.835) | <0.001 |  | 0.697(0.579,0.839) | <0.001 |  | 0.695(0.578,0.836) | <0.001 |  |
| **Q3** | 0.703(0.603,0.820) | <0.001 |  | 0.582(0.490,0.690) | <0.001 |  | 0.585(0.486,0.703) | <0.001 |  | 0.589(0.487,0.711) | <0.001 |  |
| **Q4** | 0.447(0.373,0.536) | <0.001 |  | 0.374(0.307,0.455) | <0.001 |  | 0.380(0.307,0.469) | <0.001 |  | 0.385(0.309,0.479) | <0.001 |  |
| **Serum β-carotene (μmol/L)** |  |  | <0.001 |  |  | <0.001 |  |  | <0.001 |  |  | <0.001 |
| **Q1** | 1.000(ref) |  |  | 1.000(ref) |  |  | 1.000(ref) |  |  | 1.000(ref) |  |  |
| **Q2** | 0.737(0.627,0.866) | <0.001 |  | 0.672(0.557,0.810) | <0.001 |  | 0.661(0.543,0.803) | <0.001 |  | 0.657(0.537,0.802) | <0.001 |  |
| **Q3** | 0.616(0.522,0.727) | <0.001 |  | 0.491(0.406,0.594) | <0.001 |  | 0.475(0.389,0.581) | <0.001 |  | 0.471(0.384,0.578) | <0.001 |  |
| **Q4** | 0.391(0.331,0.461) | <0.001 |  | 0.278(0.228,0.339) | <0.001 |  | 0.274(0.224,0.336) | <0.001 |  | 0.273(0.220,0.337) | <0.001 |  |

Crude Model: unadjusted for any factor

Model 1: adjusted for demographic characteristics (age, gender, race, education, marital status, poverty-income ratio, and health insurance)

Model 2: adjusted for Model 1+lifestyle factors (smoking status, drinking condition, and physical activity)

Model 3: adjusted for Model 2+health conditions (arteriosclerotic cardiovascular disease, chronic kidney disease, liver condition, thyroid disease, and cancer)

**Table S4 Odds ratio (95% CI) for blood glucose elevating according to dietary and serum antioxidant vitamins in NHANES population**

| **Antioxidant vitamin** | **Crude Model** | |  | **Model 1** | |  | **Model 2** | |  | **Model 3** | | |
| --- | --- | --- | --- | --- | --- | --- | --- | --- | --- | --- | --- | --- |
|  | **OR (95%CI)** | ***P*-value** | **P- trend** | **OR (95%CI)** | ***P*-value** | **P- trend** | **OR (95%CI)** | ***P*-value** | ***P*-trend** | **OR (95%CI)** | ***P*-value** | **P- trend** |
| **Dietary vitamin A (mcg)** |  |  | 0.665 |  |  | 0.257 |  |  | 0.234 |  |  | 0.321 |
| **Q1** | 1.000(ref) |  |  | 1.000(ref) |  |  | 1.000(ref) |  |  | 1.000(ref) |  |  |
| **Q2** | 1.178(1.005,1.381) | 0.044 |  | 1.134(0.975,1.319) | 0.100 |  | 1.131(0.974,1.312) | 0.102 |  | 1.119(0.965,1.297) | 0.129 |  |
| **Q3** | 1.155(0.973,1.371) | 0.098 |  | 1.052(0.881,1.255) | 0.566 |  | 1.043(0.878,1.238) | 0.617 |  | 1.047(0.879,1.246) | 0.589 |  |
| **Q4** | 1.018(0.851,1.218) | 0.840 |  | 0.956(0.797,1.146) | 0.612 |  | 0.950(0.792,1.139) | 0.563 |  | 0.960(0.798,1.154) | 0.647 |  |
| **Dietary vitamin C (mg)** |  |  | 0.147 |  |  | 0.023 |  |  | 0.024 |  |  |  |
| **Q1** | 1.000(ref) |  |  | 1.000(ref) |  |  | 1.000(ref) |  |  | 1.000(ref) |  |  |
| **Q2** | 1.073(0.905,1.272) | 0.408 |  | 1.028(0.857,1.233) | 0.757 |  | 1.021(0.846,1.232) | 0.818 |  | 1.022(0.842,1.242) | 0.814 |  |
| **Q3** | 1.151(0.963,1.376) | 0.12 |  | 1.037(0.855,1.258) | 0.702 |  | 1.023(0.839,1.247) | 0.815 |  | 1.024(0.841,1.248) | 0.801 |  |
| **Q4** | 0.905(0.775,1.056) | 0.197 |  | 0.834(0.706,0.984) | 0.033 |  | 0.831(0.700,0.987) | 0.036 |  | 0.837(0.703,0.996) | 0.045 |  |
| **Dietary vitamin E (mg)** |  |  | 0.182 |  |  | 0.67 |  |  | 0.629 |  |  | 0.995 |
| **Q1** | 1.000(ref) |  |  | 1.000(ref) |  |  | 1.000(ref) |  |  | 1.000(ref) |  |  |
| **Q2** | 0.983(0.842,1.147) | 0.821 |  | 0.995(0.850,1.165) | 0.952 |  | 1.000(0.850,1.175) | 0.996 |  | 1.025(0.871,1.207) | 0.752 |  |
| **Q3** | 0.845(0.717,0.996) | 0.044 |  | 0.876(0.754,1.018) | 0.083 |  | 0.882(0.761,1.022) | 0.091 |  | 0.920(0.792,1.068) | 0.255 |  |
| **Q4** | 0.917(0.788,1.067) | 0.254 |  | 0.973(0.831,1.140) | 0.729 |  | 0.971(0.829,1.137) | 0.702 |  | 1.012(0.861,1.189) | 0.88 |  |
| **Dietary carotene (mcg)** |  |  | 0.639 |  |  | 0.48 |  |  | 0.473 |  |  | 0.562 |
| **Q1** | ref | ref |  | ref | ref |  | ref | ref |  | ref | ref | ref |
| **Q2** | 1.045(0.880,1.240) | 0.61 |  | 1.036(0.868,1.237) | 0.683 |  | 1.028(0.862,1.225) | 0.751 |  | 1.042(0.868,1.250) | 0.645 |  |
| **Q3** | 1.188(1.022,1.381) | 0.026 |  | 1.114(0.958,1.295) | 0.154 |  | 1.098(0.942,1.280) | 0.217 |  | 1.096(0.937,1.281) | 0.235 |  |
| **Q4** | 1.070(0.917,1.250) | 0.381 |  | 0.970(0.822,1.146) | 0.715 |  | 0.962(0.803,1.153) | 0.663 |  | 0.978(0.811,1.179) | 0.802 |  |
| **Dietary α-carotene (mcg)** |  |  | 0.752 |  |  | 0.428 |  |  | 0.425 |  |  | 0.542 |
| **Q1** | 1.000(ref) |  |  | 1.000(ref) |  |  | 1.000(ref) |  |  | 1.000(ref) |  |  |
| **Q2** | 1.140(0.977,1.330) | 0.094 |  | 1.112(0.946,1.306) | 0.189 |  | 1.104(0.937,1.301) | 0.225 |  | 1.113(0.935,1.325) | 0.212 |  |
| **Q3** | 1.405(1.161,1.701) | <0.001 |  | 1.265(1.023,1.564) | 0.031 |  | 1.254(1.016,1.548) | 0.036 |  | 1.264(1.024,1.560) | 0.031 |  |
| **Q4** | 1.155(0.988,1.351) | 0.069 |  | 1.044(0.893,1.220) | 0.58 |  | 1.038(0.883,1.220) | 0.634 |  | 1.057(0.893,1.252) | 0.497 |  |
| **Dietary β-carotene (mcg)** |  |  | 0.521 |  |  | 0.697 |  |  | 0.673 |  |  | 0.764 |
| **Q1** | 1.000(ref) |  |  | 1.000(ref) |  |  | 1.000(ref) |  |  | 1.000(ref) |  |  |
| **Q2** | 0.983(0.840,1.150) | 0.825 |  | 0.969(0.829,1.133) | 0.682 |  | 0.960(0.822,1.121) | 0.588 |  | 0.972(0.827,1.142) | 0.713 |  |
| **Q3** | 1.149(0.986,1.340) | 0.075 |  | 1.070(0.918,1.247) | 0.375 |  | 1.057(0.907,1.232) | 0.462 |  | 1.059(0.907,1.236) | 0.446 |  |
| **Q4** | 1.053(0.901,1.230) | 0.509 |  | 0.963(0.814,1.140) | 0.655 |  | 0.954(0.794,1.147) | 0.603 |  | 0.969(0.800,1.173) | 0.733 |  |
| **Serum vitamin A (μmol/L)** |  |  | <0.001 |  |  | <0.001 |  |  | <0.001 |  |  | 0.01 |
| **Q1** | 1.000(ref) |  |  | 1.000(ref) |  |  | 1.000(ref) |  |  | 1.000(ref) |  |  |
| **Q2** | 1.315(1.088,1.588) | 0.006 |  | 1.157(0.952,1.407) | 0.137 |  | 1.163(0.956,1.415) | 0.125 |  | 1.173(0.963,1.429) | 0.106 |  |
| **Q3** | 1.348(1.125,1.616) | 0.002 |  | 1.081(0.902,1.296) | 0.385 |  | 1.089(0.905,1.309) | 0.351 |  | 1.083(0.901,1.303) | 0.376 |  |
| **Q4** | 1.953(1.654,2.307) | <0.001 |  | 1.393(1.158,1.675) | <0.001 |  | 1.398(1.157,1.688) | 0.001 |  | 1.311(1.075,1.599) | 0.01 |  |
| **Serum vitamin C (μmol/L)** |  |  | <0.001 |  |  | <0.001 |  |  | <0.001 |  |  | <0.001 |
| **Q1** | 1.000(ref) |  |  | 1.000(ref) |  |  | 1.000(ref) |  |  | 1.000(ref) |  |  |
| **Q2** | 0.822(0.666,1.016) | 0.068 |  | 0.848(0.651,1.104) | 0.199 |  | 0.801(0.592,1.084) | 0.129 |  | 0.798(0.526,1.212) | 0.185 |  |
| **Q3** | 0.783(0.661,0.929) | 0.007 |  | 0.756(0.632,0.905) | 0.005 |  | 0.713(0.579,0.878) | 0.006 |  | 0.739(0.555,0.984) | 0.044 |  |
| **Q4** | 0.622(0.515,0.752) | <0.001 |  | 0.523(0.432,0.632) | <0.001 |  | 0.491(0.396,0.609) | <0.001 |  | 0.505(0.375,0.681) | 0.005 |  |
| **Serum vitamin E (μmol/L)** |  |  | <0.001 |  |  | 0.783 |  |  | 0.711 |  |  | 0.567 |
| **Q1** | 1.000(ref) |  |  | 1.000(ref) |  |  | 1.000(ref) |  |  | 1.000(ref) |  |  |
| **Q2** | 1.212(1.056,1.392) | 0.007 |  | 1.009(0.870,1.169) | 0.904 |  | 1.001(0.861,1.164) | 0.988 |  | 0.998(0.857,1.163) | 0.979 |  |
| **Q3** | 1.422(1.215,1.664) | <0.001 |  | 0.992(0.822,1.195) | 0.926 |  | 0.991(0.821,1.197) | 0.923 |  | 1.001(0.827,1.213) | 0.989 |  |
| **Q4** | 1.749(1.448,2.112) | <0.001 |  | 0.977(0.778,1.227) | 0.837 |  | 0.966(0.767,1.216) | 0.757 |  | 0.948(0.753,1.193) | 0.631 |  |
| **Serum carotene (μmol/L)** |  |  | <0.001 |  |  | <0.001 |  |  | <0.001 |  |  | <0.001 |
| **Q1** | 1.000(ref) |  |  | 1.000(ref) |  |  | 1.000(ref) |  |  | 1.000(ref) |  |  |
| **Q2** | 0.744(0.624,0.888) | 0.002 |  | 0.682(0.560,0.830) | <0.001 |  | 0.658(0.543,0.798) | <0.001 |  | 0.654(0.537,0.796) | <0.001 |  |
| **Q3** | 0.792(0.676,0.929) | 0.005 |  | 0.638(0.530,0.769) | <0.001 |  | 0.603(0.494,0.736) | <0.001 |  | 0.598(0.487,0.734) | <0.001 |  |
| **Q4** | 0.608(0.517,0.714) | <0.001 |  | 0.469(0.379,0.582) | <0.001 |  | 0.442(0.354,0.551) | <0.001 |  | 0.443(0.355,0.554) | <0.001 |  |
| **Serum α-carotene (μmol/L)** |  |  | <0.001 |  |  | <0.001 |  |  | <0.001 |  |  | <0.001 |
| **Q1** | 1.000(ref) |  |  | 1.000(ref) |  |  | 1.000(ref) |  |  | 1.000(ref) |  |  |
| **Q2** | 0.966(0.833,1.121) | 0.643 |  | 0.898(0.765,1.055) | 0.183 |  | 0.868(0.738,1.022) | 0.086 |  | 0.870(0.738,1.025) | 0.092 |  |
| **Q3** | 0.805(0.684,0.947) | 0.01 |  | 0.673(0.564,0.804) | <0.001 |  | 0.647(0.535,0.782) | <0.001 |  | 0.651(0.538,0.787) | <0.001 |  |
| **Q4** | 0.667(0.573,0.776) | <0.001 |  | 0.588(0.488,0.709) | <0.001 |  | 0.561(0.459,0.686) | <0.001 |  | 0.572(0.468,0.701) | <0.001 |  |
| **Serum β-carotene (μmol/L)** |  |  | <0.001 |  |  | <0.001 |  |  | <0.001 |  |  | <0.001 |
| **Q1** | 1.000(ref) |  |  | 1.000(ref) |  |  | 1.000(ref) |  |  | 1.000(ref) |  |  |
| **Q2** | 0.761(0.642,0.901) | 0.002 |  | 0.706(0.587,0.848) | <0.001 |  | 0.686(0.572,0.823) | <0.001 |  | 0.681(0.566,0.819) | <0.001 |  |
| **Q3** | 0.776(0.664,0.906) | 0.002 |  | 0.626(0.521,0.753) | <0.001 |  | 0.595(0.490,0.722) | <0.001 |  | 0.591(0.485,0.720) | <0.001 |  |
| **Q4** | 0.650(0.562,0.753) | <0.001 |  | 0.497(0.410,0.602) | <0.001 |  | 0.471(0.387,0.573) | <0.001 |  | 0.470(0.386,0.573) | <0.001 |  |

Crude Model: unadjusted for any factor

Model 1: adjusted for demographic characteristics (age, gender, race, education, marital status, poverty-income ratio, and health insurance)

Model 2: adjusted for Model 1+lifestyle factors (smoking status, drinking condition, and physical activity)

Model 3: adjusted for Model 2+health conditions (arteriosclerotic cardiovascular disease, chronic kidney disease, liver condition, thyroid disease, and cancer)

**Table S5 Odds ratio (95% CI) for HDL decreasing according to dietary and serum antioxidant vitamins in NHANES population**

| **Antioxidant vitamin** | **Crude Model** | |  | **Model 1** | |  | **Model 2** | |  | **Model 3** | | |
| --- | --- | --- | --- | --- | --- | --- | --- | --- | --- | --- | --- | --- |
|  | **OR (95%CI)** | ***P*-value** | **P- trend** | **OR (95%CI)** | ***P*-value** | **P- trend** | **OR (95%CI)** | ***P*-value** | ***P*-trend** | **OR (95%CI)** | ***P*-value** | **P- trend** |
| **Dietary vitamin A (mcg)** |  |  | <0.001 |  |  | 0.001 |  |  | 0.006 |  |  | 0.008 |
| **Q1** | ref | ref |  | ref | ref |  | ref | ref |  | ref | ref |  |
| **Q2** | 0.825(0.702,0.969) | 0.020 |  | 0.888(0.755,1.044) | 0.143 |  | 0.912(0.773,1.075) | 0.259 |  | 0.910(0.769,1.076) | 0.251 |  |
| **Q3** | 0.776(0.668,0.901) | 0.001 |  | 0.892(0.764,1.042) | 0.143 |  | 0.922(0.792,1.073) | 0.281 |  | 0.925(0.794,1.077) | 0.295 |  |
| **Q4** | 0.635(0.546,0.737) | <0.001 |  | 0.768(0.668,0.882) | <0.001 |  | 0.803(0.695,0.926) | 0.004 |  | 0.808(0.699,0.934) | 0.006 |  |
| **Dietary vitamin C (mg)** |  |  | <0.001 |  |  | 0.003 |  |  | 0.037 |  |  | 0.046 |
| **Q1** | ref | ref |  | ref | ref |  | ref | ref |  | ref | ref |  |
| **Q2** | 0.882(0.745,1.045) | 0.143 |  | 0.950(0.808,1.117) | 0.522 |  | 0.995(0.842,1.176) | 0.952 |  | 0.997(0.842,1.179) | 0.967 |  |
| **Q3** | 0.720(0.613,0.846) | <0.001 |  | 0.802(0.684,0.941) | 0.009 |  | 0.850(0.726,0.995) | 0.044 |  | 0.850(0.724,0.998) | 0.048 |  |
| **Q4** | 0.694(0.597,0.808) | <0.001 |  | 0.820(0.704,0.954) | 0.012 |  | 0.881(0.750,1.034) | 0.115 |  | 0.885(0.751,1.042) | 0.134 |  |
| **Dietary vitamin E (mg)** |  |  | <0.001 |  |  | <0.001 |  |  | 0.003 |  |  | 0.005 |
| **Q1** | ref | ref |  | ref | ref |  | ref | ref |  | ref | ref |  |
| **Q2** | 0.836(0.735,0.950) | 0.007 |  | 0.905(0.787,1.042) | 0.159 |  | 0.925(0.799,1.070) | 0.278 |  | 0.931(0.803,1.080) | 0.326 |  |
| **Q3** | 0.687(0.587,0.804) | <0.001 |  | 0.805(0.679,0.955) | 0.015 |  | 0.831(0.694,0.995) | 0.045 |  | 0.840(0.697,1.013) | 0.066 |  |
| **Q4** | 0.596(0.513,0.694) | <0.001 |  | 0.747(0.639,0.874) | <0.001 |  | 0.777(0.662,0.911) | 0.003 |  | 0.787(0.668,0.926) | 0.006 |  |
| **Dietary carotene (mcg)** |  |  | <0.001 |  |  | 0.006 |  |  | 0.016 |  |  | 0.022 |
| **Q1** | ref | ref |  | ref | ref |  | ref | ref |  | ref | ref |  |
| **Q2** | 0.805(0.699,0.928) | 0.004 |  | 0.897(0.771,1.044) | 0.153 |  | 0.921(0.792,1.070) | 0.268 |  | 0.926(0.793,1.080) | 0.308 |  |
| **Q3** | 0.762(0.654,0.888) | <0.001 |  | 0.869(0.736,1.027) | 0.097 |  | 0.910(0.766,1.080) | 0.266 |  | 0.911(0.765,1.085) | 0.277 |  |
| **Q4** | 0.660(0.566,0.770) | <0.001 |  | 0.765(0.647,0.905) | 0.003 |  | 0.798(0.672,0.947) | 0.012 |  | 0.806(0.677,0.959) | 0.018 |  |
| **Dietary α-carotene (mcg)** |  |  | 0.009 |  |  | 0.052 |  |  | 0.075 |  |  | 0.095 |
| **Q1** | ref | ref |  | ref | ref |  | ref | ref |  | ref | ref |  |
| **Q2** | 0.849(0.721,0.999) | 0.049 |  | 0.914(0.771,1.083) | 0.288 |  | 0.955(0.813,1.122) | 0.56 |  | 0.960(0.814,1.132) | 0.608 |  |
| **Q3** | 0.812(0.681,0.969) | 0.022 |  | 0.901(0.756,1.073) | 0.233 |  | 0.946(0.796,1.125) | 0.517 |  | 0.952(0.800,1.133) | 0.56 |  |
| **Q4** | 0.741(0.616,0.892) | 0.002 |  | 0.809(0.662,0.989) | 0.039 |  | 0.844(0.692,1.029) | 0.09 |  | 0.853(0.696,1.045) | 0.116 |  |
| **Dietary β-carotene (mcg)** |  |  | <0.001 |  |  | 0.005 |  |  | 0.016 |  |  | 0.021 |
| **Q1** | ref | ref |  | ref | ref |  | ref | ref |  | ref | ref |  |
| **Q2** | 0.816(0.702,0.948) | 0.009 |  | 0.905(0.770,1.064) | 0.218 |  | 0.928(0.788,1.093) | 0.354 |  | 0.932(0.789,1.102) | 0.391 |  |
| **Q3** | 0.746(0.638,0.872) | <0.001 |  | 0.850(0.720,1.003) | 0.054 |  | 0.884(0.744,1.051) | 0.154 |  | 0.886(0.744,1.056) | 0.165 |  |
| **Q4** | 0.645(0.547,0.762) | <0.001 |  | 0.749(0.625,0.898) | 0.003 |  | 0.786(0.654,0.945) | 0.013 |  | 0.793(0.659,0.954) | 0.017 |  |
| **Serum vitamin A (μmol/L)** |  |  | <0.001 |  |  | <0.001 |  |  | <0.001 |  |  | <0.001 |
| **Q1** | ref | ref |  | ref | ref |  | ref | ref |  | ref | ref |  |
| **Q2** | 0.821(0.719,0.938) | 0.005 |  | 0.897(0.782,1.030) | 0.118 |  | 0.917(0.793,1.060) | 0.228 |  | 0.917(0.792,1.062) | 0.233 |  |
| **Q3** | 0.590(0.500,0.696) | <0.001 |  | 0.677(0.564,0.813) | <0.001 |  | 0.695(0.576,0.838) | <0.001 |  | 0.695(0.573,0.843) | <0.001 |  |
| **Q4** | 0.571(0.499,0.653) | <0.001 |  | 0.699(0.592,0.826) | <0.001 |  | 0.736(0.621,0.873) | 0.001 |  | 0.720(0.604,0.857) | <0.001 |  |
| **Serum vitamin C (μmol/L)** |  |  | <0.001 |  |  | <0.001 |  |  | <0.001 |  |  | <0.001 |
| **Q1** | ref | ref |  | ref | ref |  | ref | ref |  | ref | ref |  |
| **Q2** | 0.815(0.649,1.024) | 0.077 |  | 0.868(0.686,1.097) | 0.215 |  | 0.911(0.702,1.183) | 0.436 |  | 0.911(0.635,1.306) | 0.47 |  |
| **Q3** | 0.556(0.455,0.679) | <0.001 |  | 0.570(0.463,0.703) | <0.001 |  | 0.602(0.477,0.759) | 0.001 |  | 0.608(0.437,0.844) | 0.017 |  |
| **Q4** | 0.418(0.325,0.537) | <0.001 |  | 0.401(0.318,0.505) | <0.001 |  | 0.435(0.336,0.562) | <0.001 |  | 0.438(0.305,0.628) | 0.005 |  |
| **Serum vitamin E (μmol/L)** |  |  | 0.967 |  |  | 0.028 |  |  | 0.005 |  |  | 0.006 |
| **Q1** | ref | ref |  | ref | ref |  | ref | ref |  | ref | ref |  |
| **Q2** | 0.791(0.671,0.932) | 0.006 |  | 0.849(0.726,0.993) | 0.041 |  | 0.856(0.732,1.001) | 0.051 |  | 0.856(0.732,1.001) | 0.051 |  |
| **Q3** | 0.876(0.740,1.036) | 0.119 |  | 0.996(0.849,1.169) | 0.964 |  | 1.037(0.880,1.221) | 0.654 |  | 1.044(0.887,1.229) | 0.588 |  |
| **Q4** | 0.934(0.802,1.087) | 0.367 |  | 1.108(0.945,1.298) | 0.199 |  | 1.174(0.996,1.383) | 0.055 |  | 1.176(0.996,1.388) | 0.055 |  |
| **Serum carotene (μmol/L)** |  |  | <0.001 |  |  | <0.001 |  |  | <0.001 |  |  | <0.001 |
| **Q1** | ref | ref |  | ref | ref |  | ref | ref |  | ref | ref |  |
| **Q2** | 0.752(0.635,0.891) | 0.002 |  | 0.717(0.606,0.849) | <0.001 |  | 0.727(0.613,0.861) | <0.001 |  | 0.728(0.611,0.868) | 0.001 |  |
| **Q3** | 0.590(0.485,0.716) | <0.001 |  | 0.557(0.461,0.673) | <0.001 |  | 0.571(0.471,0.692) | <0.001 |  | 0.572(0.470,0.695) | <0.001 |  |
| **Q4** | 0.345(0.279,0.426) | <0.001 |  | 0.317(0.259,0.387) | <0.001 |  | 0.334(0.272,0.411) | <0.001 |  | 0.338(0.273,0.419) | <0.001 |  |
| **Serum α-carotene (μmol/L)** |  |  | <0.001 |  |  | <0.001 |  |  | <0.001 |  |  | <0.001 |
| **Q1** | ref | ref |  | ref | ref |  | ref | ref |  | ref | ref |  |
| **Q2** | 0.714(0.591,0.862) | <0.001 |  | 0.693(0.570,0.841) | <0.001 |  | 0.719(0.589,0.878) | 0.002 |  | 0.721(0.588,0.884) | 0.003 |  |
| **Q3** | 0.634(0.524,0.767) | <0.001 |  | 0.616(0.512,0.742) | <0.001 |  | 0.650(0.539,0.786) | <0.001 |  | 0.655(0.540,0.794) | <0.001 |  |
| **Q4** | 0.428(0.356,0.515) | <0.001 |  | 0.414(0.347,0.493) | <0.001 |  | 0.447(0.370,0.540) | <0.001 |  | 0.452(0.372,0.550) | <0.001 |  |
| **Serum β-carotene (μmol/L)** |  |  | <0.001 |  |  | <0.001 |  |  | <0.001 |  |  | <0.001 |
| **Q1** | ref | ref |  | ref | ref |  | ref | ref |  | ref | ref |  |
| **Q2** | 0.813(0.686,0.964) | 0.018 |  | 0.785(0.662,0.930) | 0.007 |  | 0.794(0.666,0.945) | 0.012 |  | 0.794(0.664,0.949) | 0.014 |  |
| **Q3** | 0.591(0.488,0.716) | <0.001 |  | 0.565(0.466,0.685) | <0.001 |  | 0.576(0.472,0.702) | <0.001 |  | 0.578(0.472,0.707) | <0.001 |  |
| **Q4** | 0.363(0.292,0.450) | <0.001 |  | 0.334(0.274,0.409) | <0.001 |  | 0.351(0.287,0.429) | <0.001 |  | 0.355(0.288,0.436) | <0.001 |  |

Crude Model: unadjusted for any factor

Model 1: adjusted for demographic characteristics (age, gender, race, education, marital status, poverty-income ratio, and health insurance)

Model 2: adjusted for Model 1+lifestyle factors (smoking status, drinking condition, and physical activity)

Model 3: adjusted for Model 2+health conditions (arteriosclerotic cardiovascular disease, chronic kidney disease, liver condition, thyroid disease, and cancer)

**Table S6 Odds ratio (95% CI) for triglyceride elevating according to dietary and serum antioxidant vitamins in NHANES population**

| **Antioxidant vitamin** | **Crude Model** | |  | **Model 1** | |  | **Model 2** | |  | **Model 3** | | |
| --- | --- | --- | --- | --- | --- | --- | --- | --- | --- | --- | --- | --- |
|  | **OR (95%CI)** | ***P*-value** | **P- trend** | **OR (95%CI)** | ***P*-value** | **P- trend** | **OR (95%CI)** | ***P*-value** | ***P*-trend** | **OR (95%CI)** | ***P*-value** | **P- trend** |
| **Dietary vitamin A (mcg)** |  |  | 0.801 |  |  | 0.413 |  |  | 0.772 |  |  | 0.863 |
| **Q1** | ref | ref |  | ref | ref |  | ref | ref |  | ref | ref |  |
| **Q2** | 1.074(0.915,1.260) | 0.373 |  | 1.029(0.880,1.203) | 0.714 |  | 1.048(0.898,1.224) | 0.535 |  | 1.039(0.890,1.213) | 0.610 |  |
| **Q3** | 0.996(0.851,1.165) | 0.956 |  | 0.920(0.782,1.082) | 0.302 |  | 0.945(0.802,1.113) | 0.479 |  | 0.947(0.804,1.117) | 0.499 |  |
| **Q4** | 1.045(0.903,1.208) | 0.549 |  | 0.960(0.834,1.106) | 0.564 |  | 1.001(0.866,1.157) | 0.989 |  | 1.005(0.865,1.168) | 0.948 |  |
| **Dietary vitamin C (mg)** |  |  | 0.063 |  |  | 0.032 |  |  | 0.133 |  |  | 0.150 |
| **Q1** | ref | ref |  | ref | ref |  | ref | ref |  | ref | ref |  |
| **Q2** | 0.959(0.807,1.141) | 0.630 |  | 0.941(0.777,1.139) | 0.518 |  | 0.969(0.798,1.178) | 0.742 |  | 0.968(0.796,1.177) | 0.731 |  |
| **Q3** | 0.969(0.846,1.111) | 0.647 |  | 0.936(0.816,1.074) | 0.335 |  | 0.974(0.849,1.119) | 0.702 |  | 0.975(0.849,1.119) | 0.7 |  |
| **Q4** | 0.864(0.735,1.014) | 0.072 |  | 0.839(0.710,0.991) | 0.04 |  | 0.886(0.745,1.055) | 0.164 |  | 0.888(0.742,1.062) | 0.179 |  |
| **Dietary vitamin E (mg)** |  |  | 0.32 |  |  | 0.027 |  |  | 0.079 |  |  | 0.121 |
| **Q1** | ref | ref |  | ref | ref |  | ref | ref |  | ref | ref |  |
| **Q2** | 0.867(0.740,1.017) | 0.078 |  | 0.829(0.703,0.977) | 0.027 |  | 0.841(0.711,0.995) | 0.044 |  | 0.850(0.717,1.007) | 0.059 |  |
| **Q3** | 0.952(0.809,1.121) | 0.546 |  | 0.903(0.763,1.069) | 0.225 |  | 0.929(0.783,1.104) | 0.387 |  | 0.947(0.795,1.127) | 0.517 |  |
| **Q4** | 0.889(0.753,1.050) | 0.162 |  | 0.799(0.674,0.946) | 0.011 |  | 0.828(0.695,0.987) | 0.036 |  | 0.843(0.706,1.008) | 0.06 |  |
| **Dietary carotene (mcg)** |  |  | 0.193 |  |  | 0.138 |  |  | 0.286 |  |  | 0.324 |
| **Q1** | ref | ref |  | ref | ref |  | ref | ref |  | ref | ref |  |
| **Q2** | 1.038(0.880,1.223) | 0.653 |  | 1.001(0.850,1.178) | 0.994 |  | 1.022(0.865,1.206) | 0.794 |  | 1.023(0.866,1.209) | 0.774 |  |
| **Q3** | 1.042(0.899,1.208) | 0.575 |  | 0.982(0.841,1.147) | 0.813 |  | 1.014(0.869,1.184) | 0.849 |  | 1.013(0.866,1.185) | 0.863 |  |
| **Q4** | 0.930(0.786,1.102) | 0.394 |  | 0.901(0.761,1.066) | 0.214 |  | 0.939(0.792,1.114) | 0.454 |  | 0.943(0.791,1.125) | 0.497 |  |
| **Dietary α-carotene (mcg)** |  |  | 0.513 |  |  | 0.22 |  |  | 0.38 |  |  | 0.434 |
| **Q1** | ref | ref |  | ref | ref |  | ref | ref |  | ref | ref |  |
| **Q2** | 1.034(0.895,1.194) | 0.645 |  | 1.010(0.873,1.170) | 0.885 |  | 1.038(0.900,1.198) | 0.592 |  | 1.044(0.904,1.206) | 0.536 |  |
| **Q3** | 1.039(0.883,1.224) | 0.635 |  | 0.987(0.836,1.165) | 0.875 |  | 1.023(0.868,1.207) | 0.773 |  | 1.026(0.870,1.211) | 0.747 |  |
| **Q4** | 0.985(0.849,1.143) | 0.841 |  | 0.933(0.798,1.090) | 0.367 |  | 0.970(0.833,1.129) | 0.681 |  | 0.977(0.837,1.141) | 0.760 |  |
| **Dietary β-carotene (mcg)** |  |  | 0.277 |  |  | 0.248 |  |  | 0.477 |  |  | 0.513 |
| **Q1** | ref | ref |  | ref | ref |  | ref | ref |  | ref | ref |  |
| **Q2** | 1.046(0.887,1.233) | 0.585 |  | 1.012(0.864,1.185) | 0.882 |  | 1.027(0.875,1.206) | 0.733 |  | 1.029(0.876,1.209) | 0.714 |  |
| **Q3** | 1.007(0.872,1.162) | 0.926 |  | 0.942(0.816,1.087) | 0.398 |  | 0.971(0.841,1.123) | 0.683 |  | 0.971(0.839,1.125) | 0.681 |  |
| **Q4** | 0.945(0.801,1.116) | 0.497 |  | 0.920(0.776,1.091) | 0.325 |  | 0.959(0.808,1.139) | 0.621 |  | 0.963(0.806,1.149) | 0.658 |  |
| **Serum vitamin A (μmol/L)** |  |  | <0.001 |  |  | <0.001 |  |  | <0.001 |  |  | <0.001 |
| **Q1** | ref | ref |  | ref | ref |  | ref | ref |  | ref | ref |  |
| **Q2** | 1.890(1.588,2.249) | <0.001 |  | 1.747(1.471,2.073) | <0.001 |  | 1.787(1.501,2.127) | <0.001 |  | 1.788(1.498,2.134) | <0.001 |  |
| **Q3** | 2.623(2.248,3.061) | <0.001 |  | 2.339(2.013,2.719) | <0.001 |  | 2.427(2.084,2.828) | <0.001 |  | 2.418(2.071,2.824) | <0.001 |  |
| **Q4** | 5.048(4.281,5.951) | <0.001 |  | 4.463(3.740,5.325) | <0.001 |  | 4.735(3.950,5.676) | <0.001 |  | 4.643(3.874,5.564) | <0.001 |  |
| **Serum vitamin C (μmol/L)** |  |  | <0.001 |  |  | <0.001 |  |  | <0.001 |  |  | 0.004 |
| **Q1** | ref | ref |  | ref | ref |  | ref | ref |  | ref | ref |  |
| **Q2** | 0.857(0.689,1.067) | 0.160 |  | 0.922(0.731,1.164) | 0.465 |  | 0.945(0.741,1.206) | 0.610 |  | 0.949(0.676,1.331) | 0.655 |  |
| **Q3** | 0.725(0.595,0.884) | 0.002 |  | 0.748(0.607,0.920) | 0.01 |  | 0.782(0.623,0.982) | 0.037 |  | 0.796(0.576,1.099) | 0.110 |  |
| **Q4** | 0.572(0.458,0.713) | <0.001 |  | 0.591(0.490,0.713) | <0.001 |  | 0.626(0.510,0.769) | <0.001 |  | 0.633(0.473,0.846) | 0.015 |  |
| **Serum vitamin E (μmol/L)** |  |  | <0.001 |  |  | <0.001 |  |  | <0.001 |  |  | <0.001 |
| **Q1** | ref | ref |  | ref | ref |  | ref | ref |  | ref | ref |  |
| **Q2** | 2.658(2.125,3.325) | <0.001 |  | 2.981(2.353,3.779) | <0.001 |  | 3.004(2.389,3.778) | <0.001 |  | 3.011(2.387,3.797) | <0.001 |  |
| **Q3** | 4.804(3.944,5.850) | <0.001 |  | 5.644(4.517,7.052) | <0.001 |  | 5.942(4.781,7.383) | <0.001 |  | 6.006(4.811,7.497) | <0.001 |  |
| **Q4** | 9.550(7.746,11.774) | <0.001 |  | 12.298(9.564,15.815) | <0.001 |  | 13.374(10.419,17.169) | <0.001 |  | 13.480(10.418,17.444) | <0.001 |  |
| **Serum carotene (μmol/L)** |  |  | <0.001 |  |  | <0.001 |  |  | <0.001 |  |  | <0.001 |
| **Q1** | ref | ref |  | ref | ref |  | ref | ref |  | ref | ref |  |
| **Q2** | 0.777(0.683,0.884) | <0.001 |  | 0.758(0.659,0.872) | <0.001 |  | 0.768(0.663,0.889) | 0.001 |  | 0.767(0.659,0.893) | 0.002 |  |
| **Q3** | 0.692(0.596,0.803) | <0.001 |  | 0.628(0.537,0.734) | <0.001 |  | 0.645(0.549,0.757) | <0.001 |  | 0.641(0.545,0.756) | <0.001 |  |
| **Q4** | 0.452(0.388,0.526) | <0.001 |  | 0.414(0.350,0.488) | <0.001 |  | 0.432(0.363,0.516) | <0.001 |  | 0.434(0.362,0.521) | <0.001 |  |
| **Serum α-carotene (μmol/L)** |  |  | <0.001 |  |  | <0.001 |  |  | <0.001 |  |  | <0.001 |
| **Q1** | ref | ref |  | ref | ref |  | ref | ref |  | ref | ref |  |
| **Q2** | 0.892(0.761,1.046) | 0.155 |  | 0.850(0.717,1.008) | 0.061 |  | 0.872(0.732,1.040) | 0.121 |  | 0.871(0.728,1.042) | 0.122 |  |
| **Q3** | 0.839(0.731,0.963) | 0.014 |  | 0.759(0.658,0.874) | <0.001 |  | 0.795(0.682,0.926) | 0.005 |  | 0.798(0.682,0.934) | 0.008 |  |
| **Q4** | 0.617(0.537,0.710) | <0.001 |  | 0.595(0.509,0.696) | <0.001 |  | 0.637(0.543,0.747) | <0.001 |  | 0.643(0.548,0.755) | <0.001 |  |
| **Serum β-carotene (μmol/L)** |  |  | <0.001 |  |  | <0.001 |  |  | <0.001 |  |  | <0.001 |
| **Q1** | ref | ref |  | ref | ref |  | ref | ref |  | ref | ref |  |
| **Q2** | 0.747(0.657,0.850) | <0.001 |  | 0.733(0.642,0.837) | <0.001 |  | 0.744(0.648,0.854) | <0.001 |  | 0.743(0.644,0.856) | <0.001 |  |
| **Q3** | 0.674(0.590,0.771) | <0.001 |  | 0.617(0.533,0.712) | <0.001 |  | 0.630(0.543,0.731) | <0.001 |  | 0.628(0.539,0.730) | <0.001 |  |
| **Q4** | 0.439(0.379,0.510) | <0.001 |  | 0.400(0.342,0.467) | <0.001 |  | 0.417(0.354,0.492) | <0.001 |  | 0.419(0.354,0.495) | <0.001 |  |

Crude Model: unadjusted for any factor

Model 1: adjusted for demographic characteristics (age, gender, race, education, marital status, poverty-income ratio, and health insurance)

Model 2: adjusted for Model 1+lifestyle factors (smoking status, drinking condition, and physical activity)

Model 3: adjusted for Model 2+health conditions (arteriosclerotic cardiovascular disease, chronic kidney disease, liver condition, thyroid disease, and cancer)

**Table S7 Odds ratio (95% CI) for blood pressure elevating according to dietary and serum antioxidant vitamins in NHANES population**

| **Antioxidant vitamin** | **Crude Model** | |  | **Model 1** | |  | **Model 2** | |  | **Model 3** | | |
| --- | --- | --- | --- | --- | --- | --- | --- | --- | --- | --- | --- | --- |
|  | **OR (95%CI)** | ***P*-value** | **P- trend** | **OR (95%CI)** | ***P*-value** | **P- trend** | **OR (95%CI)** | ***P*-value** | ***P*-trend** | **OR (95%CI)** | ***P*-value** | **P- trend** |
| **Dietary vitamin A (mcg)** |  |  | 0.412 |  |  | 0.039 |  |  | 0.04 |  |  | 0.078 |
| **Q1** | ref | ref |  | ref | ref |  | ref | ref |  | ref | ref |  |
| **Q2** | 0.985(0.825,1.176) | 0.864 |  | 0.893(0.754,1.057) | 0.181 |  | 0.890(0.749,1.056) | 0.172 |  | 0.873(0.735,1.038) | 0.117 |  |
| **Q3** | 1.057(0.904,1.236) | 0.478 |  | 0.887(0.756,1.040) | 0.134 |  | 0.879(0.745,1.037) | 0.121 |  | 0.882(0.747,1.043) | 0.133 |  |
| **Q4** | 0.936(0.802,1.092) | 0.39 |  | 0.817(0.693,0.963) | 0.018 |  | 0.811(0.683,0.963) | 0.019 |  | 0.820(0.686,0.981) | 0.032 |  |
| **Dietary vitamin C (mg)** |  |  | 0.473 |  |  | 0.326 |  |  | 0.343 |  |  | 0.39 |
| **Q1** | ref | ref |  | ref | ref |  | ref | ref |  | ref | ref |  |
| **Q2** | 1.066(0.937,1.213) | 0.322 |  | 1.010(0.872,1.171) | 0.886 |  | 1.005(0.864,1.170) | 0.944 |  | 1.007(0.869,1.166) | 0.926 |  |
| **Q3** | 1.080(0.901,1.294) | 0.399 |  | 0.962(0.793,1.165) | 0.679 |  | 0.952(0.782,1.158) | 0.607 |  | 0.952(0.777,1.166) | 0.618 |  |
| **Q4** | 0.955(0.800,1.140) | 0.602 |  | 0.915(0.748,1.120) | 0.376 |  | 0.916(0.745,1.126) | 0.388 |  | 0.924(0.750,1.140) | 0.44 |  |
| **Dietary vitamin E (mg)** |  |  | 0.075 |  |  | 0.42 |  |  | 0.409 |  |  | 0.808 |
| **Q1** | ref | ref |  | ref | ref |  | ref | ref |  | ref | ref |  |
| **Q2** | 0.863(0.731,1.019) | 0.081 |  | 0.859(0.715,1.032) | 0.101 |  | 0.863(0.714,1.043) | 0.121 |  | 0.887(0.729,1.078) | 0.213 |  |
| **Q3** | 0.807(0.702,0.927) | 0.003 |  | 0.822(0.714,0.946) | 0.008 |  | 0.827(0.718,0.953) | 0.011 |  | 0.866(0.745,1.005) | 0.058 |  |
| **Q4** | 0.848(0.735,0.979) | 0.025 |  | 0.897(0.762,1.056) | 0.184 |  | 0.896(0.757,1.060) | 0.191 |  | 0.945(0.796,1.121) | 0.495 |  |
| **Dietary carotene (mcg)** |  |  | 0.902 |  |  | 0.046 |  |  | 0.042 |  |  | 0.078 |
| **Q1** | ref | ref |  | ref | ref |  | ref | ref |  | ref | ref |  |
| **Q2** | 1.109(0.949,1.295) | 0.188 |  | 1.097(0.921,1.305) | 0.287 |  | 1.089(0.909,1.304) | 0.339 |  | 1.105(0.925,1.319) | 0.254 |  |
| **Q3** | 1.161(0.982,1.371) | 0.078 |  | 1.047(0.873,1.255) | 0.612 |  | 1.034(0.855,1.252) | 0.717 |  | 1.038(0.861,1.252) | 0.678 |  |
| **Q4** | 1.066(0.899,1.264) | 0.454 |  | 0.902(0.747,1.089) | 0.271 |  | 0.896(0.739,1.086) | 0.25 |  | 0.916(0.753,1.114) | 0.357 |  |
| **Dietary α-carotene (mcg)** |  |  | 0.641 |  |  | 0.064 |  |  | 0.063 |  |  | 0.11 |
| **Q1** | ref | ref |  | ref | ref |  | ref | ref |  | ref | ref |  |
| **Q2** | 1.037(0.909,1.184) | 0.579 |  | 0.995(0.863,1.148) | 0.947 |  | 0.989(0.857,1.140) | 0.871 |  | 1.002(0.860,1.168) | 0.978 |  |
| **Q3** | 1.106(0.958,1.277) | 0.166 |  | 0.939(0.798,1.105) | 0.437 |  | 0.931(0.788,1.100) | 0.386 |  | 0.942(0.800,1.110) | 0.455 |  |
| **Q4** | 1.008(0.865,1.173) | 0.921 |  | 0.869(0.731,1.034) | 0.109 |  | 0.864(0.724,1.031) | 0.1 |  | 0.885(0.740,1.059) | 0.171 |  |
| **Dietary β-carotene (mcg)** |  |  | 0.79 |  |  | 0.083 |  |  | 0.076 |  |  | 0.123 |
| **Q1** | ref | ref |  | ref | ref |  | ref | ref |  | ref | ref |  |
| **Q2** | 1.148(0.992,1.329) | 0.063 |  | 1.139(0.965,1.345) | 0.12 |  | 1.130(0.953,1.341) | 0.152 |  | 1.147(0.967,1.360) | 0.108 |  |
| **Q3** | 1.138(0.951,1.361) | 0.154 |  | 1.027(0.843,1.251) | 0.784 |  | 1.017(0.828,1.249) | 0.865 |  | 1.024(0.833,1.259) | 0.811 |  |
| **Q4** | 1.089(0.922,1.286) | 0.306 |  | 0.929(0.773,1.115) | 0.415 |  | 0.921(0.764,1.111) | 0.373 |  | 0.940(0.777,1.138) | 0.508 |  |
| **Serum vitamin A (μmol/L)** |  |  | <0.001 |  |  | <0.001 |  |  | <0.001 |  |  | <0.001 |
| **Q1** | ref | ref |  | ref | ref |  | ref | ref |  | ref | ref |  |
| **Q2** | 1.408(1.132,1.753) | 0.003 |  | 1.319(1.037,1.677) | 0.025 |  | 1.327(1.042,1.689) | 0.024 |  | 1.325(1.040,1.689) | 0.025 |  |
| **Q3** | 1.593(1.285,1.973) | <0.001 |  | 1.405(1.125,1.754) | 0.004 |  | 1.418(1.133,1.775) | 0.004 |  | 1.406(1.120,1.765) | 0.006 |  |
| **Q4** | 2.503(2.043,3.067) | <0.001 |  | 1.988(1.598,2.473) | <0.001 |  | 2.002(1.605,2.496) | <0.001 |  | 1.858(1.488,2.320) | <0.001 |  |
| **Serum vitamin C (μmol/L)** |  |  | 0.003 |  |  | 0.002 |  |  | 0.002 |  |  | 0.013 |
| **Q1** | ref | ref |  | ref | ref |  | ref | ref |  | ref | ref |  |
| **Q2** | 0.872(0.713,1.067) | 0.175 |  | 0.910(0.718,1.154) | 0.407 |  | 0.876(0.680,1.128) | 0.262 |  | 0.881(0.617,1.259) | 0.342 |  |
| **Q3** | 0.666(0.537,0.826) | <0.001 |  | 0.661(0.539,0.812) | <0.001 |  | 0.637(0.515,0.789) | 0.001 |  | 0.669(0.496,0.901) | 0.023 |  |
| **Q4** | 0.702(0.541,0.911) | 0.01 |  | 0.657(0.497,0.868) | 0.006 |  | 0.635(0.477,0.844) | 0.006 |  | 0.661(0.440,0.991) | 0.047 |  |
| **Serum vitamin E (μmol/L)** |  |  | <0.001 |  |  | <0.001 |  |  | <0.001 |  |  | <0.001 |
| **Q1** | ref | ref |  | ref | ref |  | ref | ref |  | ref | ref |  |
| **Q2** | 1.583(1.302,1.924) | <0.001 |  | 1.336(1.104,1.615) | 0.004 |  | 1.330(1.103,1.604) | 0.004 |  | 1.333(1.104,1.609) | 0.005 |  |
| **Q3** | 1.950(1.624,2.342) | <0.001 |  | 1.423(1.171,1.729) | <0.001 |  | 1.434(1.184,1.736) | <0.001 |  | 1.467(1.206,1.784) | <0.001 |  |
| **Q4** | 2.878(2.431,3.408) | <0.001 |  | 1.687(1.456,1.955) | <0.001 |  | 1.687(1.459,1.950) | <0.001 |  | 1.683(1.443,1.963) | <0.001 |  |
| **Serum carotene (μmol/L)** |  |  | 0.017 |  |  | <0.001 |  |  | <0.001 |  |  | <0.001 |
| **Q1** | ref | ref |  | ref | ref |  | ref | ref |  | ref | ref |  |
| **Q2** | 0.839(0.719,0.978) | 0.026 |  | 0.761(0.646,0.897) | 0.002 |  | 0.742(0.628,0.877) | 0.001 |  | 0.736(0.619,0.875) | 0.002 |  |
| **Q3** | 0.936(0.814,1.077) | 0.349 |  | 0.752(0.647,0.873) | <0.001 |  | 0.722(0.621,0.839) | <0.001 |  | 0.712(0.615,0.825) | <0.001 |  |
| **Q4** | 0.779(0.658,0.922) | 0.005 |  | 0.573(0.475,0.692) | <0.001 |  | 0.549(0.452,0.668) | <0.001 |  | 0.557(0.456,0.680) | <0.001 |  |
| **Serum α-carotene (μmol/L)** |  |  | 0.004 |  |  | <0.001 |  |  | <0.001 |  |  | 0.002 |
| **Q1** | ref | ref |  | ref | ref |  | ref | ref |  | ref | ref |  |
| **Q2** | 0.937(0.777,1.131) | 0.491 |  | 0.871(0.716,1.061) | 0.164 |  | 0.847(0.697,1.030) | 0.092 |  | 0.846(0.700,1.022) | 0.079 |  |
| **Q3** | 1.002(0.854,1.176) | 0.981 |  | 0.860(0.715,1.035) | 0.107 |  | 0.838(0.691,1.017) | 0.072 |  | 0.850(0.697,1.037) | 0.103 |  |
| **Q4** | 0.777(0.651,0.927) | 0.006 |  | 0.677(0.552,0.830) | <0.001 |  | 0.656(0.531,0.811) | <0.001 |  | 0.673(0.540,0.839) | 0.001 |  |
| **Serum β-carotene (μmol/L)** |  |  | 0.026 |  |  | <0.001 |  |  | <0.001 |  |  | <0.001 |
| **Q1** | ref | ref |  | ref | ref |  | ref | ref |  | ref | ref |  |
| **Q2** | 0.873(0.754,1.011) | 0.068 |  | 0.802(0.682,0.942) | 0.009 |  | 0.787(0.670,0.926) | 0.006 |  | 0.779(0.659,0.920) | 0.006 |  |
| **Q3** | 0.929(0.800,1.078) | 0.322 |  | 0.742(0.633,0.868) | <0.001 |  | 0.713(0.608,0.837) | <0.001 |  | 0.706(0.606,0.823) | <0.001 |  |
| **Q4** | 0.793(0.661,0.951) | 0.013 |  | 0.568(0.464,0.695) | <0.001 |  | 0.546(0.445,0.671) | <0.001 |  | 0.550(0.447,0.677) | <0.001 |  |

Crude Model: unadjusted for any factor

Model 1: adjusted for demographic characteristics (age, gender, race, education, marital status, poverty-income ratio, and health insurance)

Model 2: adjusted for Model 1+lifestyle factors (smoking status, drinking condition, and physical activity)

Model 3: adjusted for Model 2+health conditions (arteriosclerotic cardiovascular disease, chronic kidney disease, liver condition, thyroid disease, and cancer)

**Table S8 Odds ratio (95% CI) for obesity according to dietary and serum antioxidant vitamins in NHANES population**

| **Antioxidant vitamin** | **Crude Model** | |  | **Model 1** | |  | **Model 2** | |  | **Model 3** | | |
| --- | --- | --- | --- | --- | --- | --- | --- | --- | --- | --- | --- | --- |
|  | **OR (95%CI)** | ***P*-value** | **P- trend** | **OR (95%CI)** | ***P*-value** | **P- trend** | **OR (95%CI)** | ***P*-value** | ***P*-trend** | **OR (95%CI)** | ***P*-value** | **P- trend** |
| **Dietary vitamin A (mcg)** |  |  | 0.003 |  |  | 0.02 |  |  | 0.029 |  |  | 0.045 |
| **Q1** | ref | ref |  | ref | ref |  | ref | ref |  | ref | ref |  |
| **Q2** | 0.979(0.873,1.097) | 0.707 |  | 0.930(0.821,1.053) | 0.240 |  | 0.936(0.828,1.057) | 0.273 |  | 0.933(0.822,1.058) | 0.260 |  |
| **Q3** | 0.963(0.822,1.128) | 0.633 |  | 0.887(0.763,1.032) | 0.115 |  | 0.887(0.765,1.028) | 0.107 |  | 0.893(0.768,1.039) | 0.134 |  |
| **Q4** | 0.824(0.728,0.933) | 0.003 |  | 0.832(0.727,0.952) | 0.009 |  | 0.839(0.730,0.965) | 0.016 |  | 0.847(0.735,0.976) | 0.024 |  |
| **Dietary vitamin C (mg)** |  |  | <0.001 |  |  | 0.002 |  |  | 0.005 |  |  | 0.007 |
| **Q1** | ref | ref |  | ref | ref |  | ref | ref |  | ref | ref |  |
| **Q2** | 0.957(0.859,1.065) | 0.411 |  | 0.929(0.827,1.045) | 0.210 |  | 0.935(0.832,1.050) | 0.241 |  | 0.938(0.832,1.057) | 0.272 |  |
| **Q3** | 0.891(0.777,1.021) | 0.095 |  | 0.823(0.708,0.958) | 0.014 |  | 0.823(0.704,0.963) | 0.017 |  | 0.820(0.699,0.962) | 0.018 |  |
| **Q4** | 0.728(0.621,0.854) | <0.001 |  | 0.752(0.635,0.890) | 0.002 |  | 0.769(0.647,0.913) | 0.004 |  | 0.777(0.653,0.925) | 0.007 |  |
| **Dietary vitamin E (mg)** |  |  | <0.001 |  |  | 0.092 |  |  | 0.127 |  |  | 0.181 |
| **Q1** | ref | ref |  | ref | ref |  | ref | ref |  | ref | ref |  |
| **Q2** | 0.907(0.792,1.039) | 0.155 |  | 0.953(0.818,1.110) | 0.520 |  | 0.967(0.831,1.125) | 0.650 |  | 0.977(0.835,1.143) | 0.759 |  |
| **Q3** | 0.807(0.714,0.913) | 0.001 |  | 0.921(0.797,1.064) | 0.253 |  | 0.939(0.809,1.089) | 0.389 |  | 0.956(0.821,1.114) | 0.546 |  |
| **Q4** | 0.680(0.585,0.791) | <0.001 |  | 0.869(0.733,1.029) | 0.100 |  | 0.881(0.743,1.045) | 0.139 |  | 0.898(0.756,1.068) | 0.209 |  |
| **Dietary carotene (mcg)** |  |  | 0.049 |  |  | <0.001 |  |  | <0.001 |  |  | 0.002 |
| **Q1** | ref | ref |  | ref | ref |  | ref | ref |  | ref | ref |  |
| **Q2** | 0.863(0.761,0.979) | 0.023 |  | 0.883(0.772,1.011) | 0.069 |  | 0.883(0.770,1.013) | 0.074 |  | 0.890(0.774,1.022) | 0.094 |  |
| **Q3** | 0.947(0.840,1.068) | 0.363 |  | 0.884(0.775,1.009) | 0.066 |  | 0.879(0.768,1.006) | 0.060 |  | 0.878(0.763,1.010) | 0.067 |  |
| **Q4** | 0.813(0.697,0.949) | 0.01 |  | 0.710(0.604,0.836) | <0.001 |  | 0.711(0.601,0.842) | <0.001 |  | 0.719(0.606,0.853) | <0.001 |  |
| **Dietary α-carotene (mcg)** |  |  | 0.103 |  |  | 0.001 |  |  | 0.002 |  |  | 0.003 |
| **Q1** | ref | ref |  | ref | ref |  | ref | ref |  | ref | ref |  |
| **Q2** | 1.066(0.939,1.209) | 0.314 |  | 1.064(0.937,1.208) | 0.329 |  | 1.075(0.934,1.238) | 0.296 |  | 1.081(0.937,1.246) | 0.266 |  |
| **Q3** | 1.118(0.966,1.294) | 0.132 |  | 1.005(0.860,1.174) | 0.952 |  | 1.013(0.859,1.194) | 0.873 |  | 1.016(0.860,1.201) | 0.842 |  |
| **Q4** | 0.958(0.839,1.093) | 0.514 |  | 0.842(0.734,0.966) | 0.016 |  | 0.848(0.735,0.978) | 0.025 |  | 0.858(0.743,0.991) | 0.039 |  |
| **Dietary β-carotene (mcg)** |  |  | 0.107 |  |  | 0.003 |  |  | 0.004 |  |  | 0.006 |
| **Q1** | ref | ref |  | ref | ref |  | ref | ref |  | ref | ref |  |
| **Q2** | 0.852(0.752,0.964) | 0.013 |  | 0.864(0.760,0.983) | 0.028 |  | 0.861(0.757,0.980) | 0.026 |  | 0.867(0.760,0.989) | 0.035 |  |
| **Q3** | 0.912(0.807,1.030) | 0.134 |  | 0.853(0.744,0.978) | 0.025 |  | 0.850(0.739,0.978) | 0.025 |  | 0.852(0.736,0.988) | 0.035 |  |
| **Q4** | 0.830(0.710,0.970) | 0.02 |  | 0.730(0.619,0.861) | <0.001 |  | 0.731(0.616,0.868) | <0.001 |  | 0.741(0.622,0.882) | 0.002 |  |
| **Serum vitamin A (μmol/L)** |  |  | 0.985 |  |  | 0.992 |  |  | 0.738 |  |  | 0.994 |
| **Q1** | ref | ref |  | ref | ref |  | ref | ref |  | ref | ref |  |
| **Q2** | 0.977(0.851,1.121) | 0.731 |  | 1.029(0.885,1.196) | 0.704 |  | 1.047(0.898,1.221) | 0.544 |  | 1.055(0.905,1.230) | 0.473 |  |
| **Q3** | 0.931(0.814,1.065) | 0.289 |  | 0.989(0.864,1.133) | 0.873 |  | 1.018(0.889,1.165) | 0.793 |  | 1.017(0.885,1.169) | 0.802 |  |
| **Q4** | 1.006(0.863,1.173) | 0.937 |  | 1.011(0.848,1.205) | 0.898 |  | 1.041(0.875,1.239) | 0.633 |  | 1.016(0.853,1.210) | 0.853 |  |
| **Serum vitamin C (μmol/L)** |  |  | <0.001 |  |  | <0.001 |  |  | <0.001 |  |  | <0.001 |
| **Q1** | ref | ref |  | ref | ref |  | ref | ref |  | ref | ref |  |
| **Q2** | 0.840(0.701,1.007) | 0.059 |  | 0.853(0.702,1.036) | 0.101 |  | 0.811(0.660,0.997) | 0.047 |  | 0.806(0.611,1.064) | 0.090 |  |
| **Q3** | 0.588(0.482,0.718) | <0.001 |  | 0.523(0.429,0.637) | <0.001 |  | 0.496(0.404,0.609) | <0.001 |  | 0.501(0.378,0.665) | 0.004 |  |
| **Q4** | 0.574(0.474,0.695) | <0.001 |  | 0.397(0.327,0.482) | <0.001 |  | 0.383(0.309,0.474) | <0.001 |  | 0.384(0.285,0.516) | 0.002 |  |
| **Serum vitamin E (μmol/L)** |  |  | <0.001 |  |  | 0.006 |  |  | 0.003 |  |  | 0.006 |
| **Q1** | ref | ref |  | ref | ref |  | ref | ref |  | ref | ref |  |
| **Q2** | 1.196(1.058,1.352) | 0.005 |  | 1.068(0.933,1.224) | 0.325 |  | 1.064(0.925,1.223) | 0.367 |  | 1.065(0.925,1.226) | 0.36 |  |
| **Q3** | 1.470(1.268,1.704) | <0.001 |  | 1.207(1.019,1.430) | 0.031 |  | 1.236(1.040,1.468) | 0.018 |  | 1.245(1.048,1.479) | 0.016 |  |
| **Q4** | 1.931(1.675,2.225) | <0.001 |  | 1.267(1.075,1.493) | 0.006 |  | 1.285(1.091,1.514) | 0.004 |  | 1.268(1.077,1.493) | 0.007 |  |
| **Serum carotene (μmol/L)** |  |  | <0.001 |  |  | <0.001 |  |  | <0.001 |  |  | <0.001 |
| **Q1** | ref | ref |  | ref | ref |  | ref | ref |  | ref | ref |  |
| **Q2** | 0.788(0.659,0.941) | 0.01 |  | 0.633(0.519,0.772) | <0.001 |  | 0.607(0.492,0.748) | <0.001 |  | 0.609(0.492,0.755) | <0.001 |  |
| **Q3** | 0.665(0.566,0.782) | <0.001 |  | 0.451(0.374,0.543) | <0.001 |  | 0.422(0.349,0.511) | <0.001 |  | 0.421(0.347,0.512) | <0.001 |  |
| **Q4** | 0.458(0.397,0.528) | <0.001 |  | 0.248(0.204,0.301) | <0.001 |  | 0.231(0.191,0.281) | <0.001 |  | 0.235(0.192,0.286) | <0.001 |  |
| **Serum α-carotene (μmol/L)** |  |  | <0.001 |  |  | <0.001 |  |  | <0.001 |  |  | <0.001 |
| **Q1** | ref | ref |  | ref | ref |  | ref | ref |  | ref | ref |  |
| **Q2** | 0.892(0.793,1.002) | 0.053 |  | 0.752(0.661,0.856) | <0.001 |  | 0.733(0.637,0.844) | <0.001 |  | 0.735(0.638,0.848) | <0.001 |  |
| **Q3** | 0.756(0.654,0.875) | <0.001 |  | 0.565(0.477,0.668) | <0.001 |  | 0.550(0.463,0.653) | <0.001 |  | 0.552(0.463,0.659) | <0.001 |  |
| **Q4** | 0.511(0.453,0.576) | <0.001 |  | 0.345(0.298,0.401) | <0.001 |  | 0.332(0.285,0.388) | <0.001 |  | 0.338(0.289,0.396) | <0.001 |  |
| **Serum β-carotene (μmol/L)** |  |  | <0.001 |  |  | <0.001 |  |  | <0.001 |  |  | <0.001 |
| **Q1** | ref | ref |  | ref | ref |  | ref | ref |  | ref | ref |  |
| **Q2** | 0.798(0.671,0.950) | 0.012 |  | 0.654(0.539,0.794) | <0.001 |  | 0.635(0.517,0.781) | <0.001 |  | 0.634(0.513,0.784) | <0.001 |  |
| **Q3** | 0.651(0.547,0.774) | <0.001 |  | 0.441(0.361,0.538) | <0.001 |  | 0.411(0.336,0.503) | <0.001 |  | 0.411(0.335,0.505) | <0.001 |  |
| **Q4** | 0.473(0.409,0.546) | <0.001 |  | 0.250(0.206,0.303) | <0.001 |  | 0.235(0.194,0.284) | <0.001 |  | 0.237(0.195,0.287) | <0.001 |  |

Crude Model: unadjusted for any factor

Model 1: adjusted for demographic characteristics (age, gender, race, education, marital status, poverty-income ratio, and health insurance)

Model 2: adjusted for Model 1+lifestyle factors (smoking status, drinking condition, and physical activity)

Model 3: adjusted for Model 2+health conditions (arteriosclerotic cardiovascular disease, chronic kidney disease, liver condition, thyroid disease, and cancer)

**Table S9 Causal effect, heterogeneity, pleiotropy of dietary and serum antioxidant vitamins on metabolic syndrome in MR analysis**

| **Antioxidative vitamin** | **Method** | **No. SNP** | **OR (95% CI)** | ***P-*value** | **Heterogeneity** | | **Pleiotropy** | | |
| --- | --- | --- | --- | --- | --- | --- | --- | --- | --- |
|  |  |  |  |  | Q | *P-*value | Intercept | SE | *P-*value |
| **Dietary antioxidative vitamin** | |  |  |  |  |  |  |  |  |
| **Vitamin A** | MR Egger | 89 | 0.883(0.752,1.037) | 0.133 | 120.272 | 0.0105 | 0.001 | 0.003 | 0.580 |
|  | Weighted median | 89 | 0.893(0.820,0.972) | 0.009 |  |  |  |  |  |
|  | Inverse variance weighted | 89 | 0.920(0.861,0.984) | 0.014 | 120.699 | 0.012 |  |  |  |
|  | Simple mode | 89 | 0.803(0.630,1.023) | 0.079 |  |  |  |  |  |
|  | Weighted mode | 89 | 0.806(0.634,1.025) | 0.082 |  |  |  |  |  |
| **Vitamin C** | MR Egger | 80 | 0.978(0.804,1.189) | 0.826 | 136.294 | <0.001 | -0.003 | 0.0038 | 0.396 |
|  | Weighted median | 80 | 0.962(0.875,1.058) | 0.426 |  |  |  |  |  |
|  | Inverse variance weighted | 80 | 0.905(0.836,0.979) | 0.013 | 137.566 | <0.001 |  |  |  |
|  | Simple mode | 80 | 0.995(0.776,1.276) | 0.971 |  |  |  |  |  |
|  | Weighted mode | 80 | 1.001(0.793,1.262) | 0.997 |  |  |  |  |  |
| **Vitamin E** | MR Egger | 77 | 0.913(0.764,1.090) | 0.316 | 120.876 | 0.001 | 0.003 | 0.003 | 0.400 |
|  | Weighted median | 77 | 0.939(0.858,1.028) | 0.173 |  |  |  |  |  |
|  | Inverse variance weighted | 77 | 0.978(0.906,1.056) | 0.570 | 122.030 | 0.001 |  |  |  |
|  | Simple mode | 77 | 0.904(0.726,1.127) | 0.372 |  |  |  |  |  |
|  | Weighted mode | 77 | 0.908(0.751,1.099) | 0.326 |  |  |  |  |  |
| **Carotene** | MR Egger | 81 | 0.940(0.812,1.088) | 0.408 | 79.798 | 0.454 | -0.001 | 0.003 | 0.729 |
|  | Weighted median | 81 | 0.922(0.848,1.003) | 0.059 |  |  |  |  |  |
|  | Inverse variance weighted | 81 | 0.918(0.865,0.974) | 0.005 | 79.920 | 0.481 |  |  |  |
|  | Simple mode | 81 | 0.921(0.753,1.125) | 0.420 |  |  |  |  |  |
|  | Weighted mode | 81 | 0.924(0.762,1.120) | 0.425 |  |  |  |  |  |
| **Absolute circulating antioxidants** | |  |  |  |  |  |  |  |  |
| **Vitamin A (Retinol)** | Inverse variance weighted | 2 | 0.959(0.704,1.308) | 0.793 | 0.290 | 0.590 |  |  |  |
| **Vitamin C (Ascorbate）** | MR Egger | 14 | 1.030(0.937,1.133) | 0.551 | 21.572 | 0.043 | -0.005 | 0.005 | 0.392 |
|  | Weighted median | 14 | 0.984(0.925,1.046) | 0.603 |  |  |  |  |  |
|  | Inverse variance weighted | 14 | 0.994(0.943,1.048) | 0.829 | 22.990 | 0.042 |  |  |  |
|  | Simple mode | 14 | 0.982(0.874,1.103) | 0.766 |  |  |  |  |  |
|  | Weighted mode | 14 | 0.986(0.933,1.041) | 0.612 |  |  |  |  |  |
| **α-Tocopherol** | Inverse variance weighted | 2 | 1.100(0.395,3.061) | 0.855 | 7.618 | 0.006 |  |  |  |
| **α-Carotene** | MR Egger | 3 | 1.113(0.496,2.497) | 0.838 | 5.352 | 0.021 | -0.021 | 0.039 | 0.684 |
|  | Weighted median | 3 | 0.905(0.802,1.022) | 0.108 |  |  |  |  |  |
|  | Inverse variance weighted | 3 | 0.897(0.764,1.052) | 0.181 | 6.922 | 0.031 |  |  |  |
|  | Simple mode | 3 | 0.851(0.709,1.020) | 0.223 |  |  |  |  |  |
|  | Weighted mode | 3 | 0.889(0.777,1.017) | 0.229 |  |  |  |  |  |
| **β-Carotene** | MR Egger | 4 | 0.804(0.693,0.933) | 0.103 | 0.778 | 0.678 | 0.018 | 0.010 | 0.225 |
|  | Weighted median | 4 | 0.897(0.841,0.957) | 0.001 |  |  |  |  |  |
|  | Inverse variance weighted | 4 | 0.909(0.857,0.965) | 0.002 | 3.790 | 0.285 |  |  |  |
|  | Simple mode | 4 | 0.910(0.831,0.996) | 0.132 |  |  |  |  |  |
|  | Weighted mode | 4 | 0.897(0.842,0.955) | 0.043 |  |  |  |  |  |
| **Circulating metabolite's concentation** | |  |  |  |  |  |  |  |  |
| **Vitamin A (Retinol)** | MR Egger | 75 | 0.975(0.944,1.007) | 0.127 | 147.424 | <0.001 | 0.007 | 0.004 | 0.082 |
|  | Weighted median | 75 | 0.979(0.966,0.992) | 0.002 |  |  |  |  |  |
|  | Inverse variance weighted | 75 | 1.001(0.989,1.014) | 0.817 | 153.720 | <0.001 |  |  |  |
|  | Simple mode | 75 | 0.972(0.942,1.002) | 0.073 |  |  |  |  |  |
|  | Weighted mode | 75 | 0.973(0.950,0.997) | 0.032 |  |  |  |  |  |
| **Vitamin C (Ascorbate）** | MR Egger | 34 | 1.001(0.899,1.114) | 0.988 | 85.213 | <0.001 | -0.002 | 0.005 | 0.607 |
|  | Weighted median | 34 | 0.983(0.927,1.042) | 0.560 |  |  |  |  |  |
|  | Inverse variance weighted | 34 | 0.977(0.924,1.033) | 0.415 | 85.933 | <0.001 |  |  |  |
|  | Simple mode | 34 | 0.990(0.897,1.092) | 0.838 |  |  |  |  |  |
|  | Weighted mode | 34 | 0.985(0.934,1.038) | 0.572 |  |  |  |  |  |
| **α-tocopherol** | MR Egger | 40 | 1.249(0.708,2.201) | 0.447 | 116.800 | <0.001 | -0.008 | 0.006 | 0.206 |
|  | Weighted median | 40 | 0.928(0.756,1.139) | 0.474 |  |  |  |  |  |
|  | Inverse variance weighted | 40 | 0.891(0.699,1.135) | 0.350 | 121.896 | <0.001 |  |  |  |
|  | Simple mode | 40 | 0.894(0.604,1.322) | 0.577 |  |  |  |  |  |
|  | Weighted mode | 40 | 0.894(0.628,1.272) | 0.536 |  |  |  |  |  |

**Table S10 Causal effect, heterogeneity, pleiotropy of dietary and serum antioxidant vitamins on blood glucose in MR analysis**

| **Antioxidative vitamin** | **Method** | **No. SNP** | **OR (95% CI)** | ***P-*value** | **Heterogeneity** | | **Pleiotropy** | | |
| --- | --- | --- | --- | --- | --- | --- | --- | --- | --- |
|  |  |  |  |  | Q | *P-*value | Intercept | SE | *P-*value |
| **Dietary antioxidative vitamin** | |  |  |  |  |  |  |  |  |
| **Vitamin A** | MR Egger | 93 | 1.019(0.978,1.062) | 0.374 | 94.200 | 0.388 | <0.001 | 0.001 | 0.702 |
|  | Weighted median | 93 | 1.009(0.984,1.035) | 0.468 |  |  |  |  |  |
|  | Inverse variance weighted | 93 | 1.012(0.994,1.029) | 0.199 | 94.353 | 0.413 |  |  |  |
|  | Simple mode | 93 | 1.040(0.967,1.119) | 0.289 |  |  |  |  |  |
|  | Weighted mode | 93 | 1.042(0.972,1.116) | 0.250 |  |  |  |  |  |
| **Vitamin C** | MR Egger | 85 | 0.951(0.909,0.995) | 0.033 | 122.101 | 0.005 | 0.001 | 0.001 | 0.187 |
|  | Weighted median | 85 | 0.976(0.949,1.003) | 0.078 |  |  |  |  |  |
|  | Inverse variance weighted | 85 | 0.979(0.960,0.999) | 0.042 | 124.648 | 0.004 |  |  |  |
|  | Simple mode | 85 | 0.971(0.903,1.045) | 0.441 |  |  |  |  |  |
|  | Weighted mode | 85 | 0.968(0.902,1.038) | 0.361 |  |  |  |  |  |
| **Vitamin E** | MR Egger | 82 | 1.008(0.966,1.052) | 0.710 | 73.335 | 0.688 | <0.001 | 0.001 | 0.730 |
|  | Weighted median | 82 | 0.999(0.973,1.025) | 0.923 |  |  |  |  |  |
|  | Inverse variance weighted | 82 | 1.015(0.997,1.034) | 0.109 | 73.455 | 0.712 |  |  |  |
|  | Simple mode | 82 | 0.982(0.921,1.046) | 0.570 |  |  |  |  |  |
|  | Weighted mode | 82 | 0.980(0.922,1.042) | 0.524 |  |  |  |  |  |
| **Carotene** | MR Egger | 83 | 0.997(0.953,1.043) | 0.899 | 65.168 | 0.900 | <0.001 | 0.001 | 0.967 |
|  | Weighted median | 83 | 0.994(0.968,1.020) | 0.654 |  |  |  |  |  |
|  | Inverse variance weighted | 83 | 0.996(0.978,1.014) | 0.679 | 65.170 | 0.914 |  |  |  |
|  | Simple mode | 83 | 0.980(0.922,1.042) | 0.527 |  |  |  |  |  |
|  | Weighted mode | 83 | 0.978(0.918,1.043) | 0.502 |  |  |  |  |  |
| **Absolute circulating antioxidants** | |  |  |  |  |  |  |  |  |
| **Vitamin A (Retinol)** | Inverse variance weighted | 2 | 1.092(0.994,1.201) | 0.068 | 1.165 | 0.280 |  |  |  |
| **Vitamin C (Ascorbate）** | MR Egger | 14 | 0.986(0.955,1.018) | 0.400 | 21.729 | 0.041 | <0.001 | 0.002 | 0.813 |
|  | Weighted median | 14 | 0.983(0.964,1.002) | 0.072 |  |  |  |  |  |
|  | Inverse variance weighted | 14 | 0.989(0.973,1.006) | 0.201 | 21.835 | 0.058 |  |  |  |
|  | Simple mode | 14 | 1.002(0.967,1.039) | 0.906 |  |  |  |  |  |
|  | Weighted mode | 14 | 0.985(0.968,1.002) | 0.113 |  |  |  |  |  |
| **α-Tocopherol** | MR Egger | 3 | 1.308(0.752,2.275) | 0.516 | 0.010 | 0.922 | 0.002 | 0.008 | 0.820 |
|  | Weighted median | 3 | 1.020(0.926,1.124) | 0.682 |  |  |  |  |  |
|  | Inverse variance weighted | 3 | 1.041(0.965,1.122) | 0.299 | 0.677 | 0.713 |  |  |  |
|  | Simple mode | 3 | 1.015(0.913,1.129) | 0.808 |  |  |  |  |  |
|  | Weighted mode | 3 | 1.014(0.904,1.136) | 0.836 |  |  |  |  |  |
| **α-Carotene** | MR Egger | 3 | 1.112(1.006,1.228) | 0.286 | 0.973 | 0.324 | -0.008 | 0.005 | 0.333 |
|  | Weighted median | 3 | 1.028(0.997,1.061) | 0.075 |  |  |  |  |  |
|  | Inverse variance weighted | 3 | 1.020(0.986,1.056) | 0.249 | 3.972 | 0.137 |  |  |  |
|  | Simple mode | 3 | 1.036(0.995,1.078) | 0.230 |  |  |  |  |  |
|  | Weighted mode | 3 | 1.031(0.998,1.065) | 0.203 |  |  |  |  |  |
| **β-Carotene** | MR Egger | 4 | 0.985(0.926,1.048) | 0.685 | 4.377 | 0.112 | 0.001 | 0.004 | 0.830 |
|  | Weighted median | 4 | 0.992(0.975,1.008) | 0.320 |  |  |  |  |  |
|  | Inverse variance weighted | 4 | 0.992(0.975,1.010) | 0.407 | 4.508 | 0.212 |  |  |  |
|  | Simple mode | 4 | 0.988(0.967,1.008) | 0.320 |  |  |  |  |  |
|  | Weighted mode | 4 | 0.991(0.976,1.007) | 0.358 |  |  |  |  |  |
| **Circulating metabolite's concentation** | |  |  |  |  |  |  |  |  |
| **Vitamin A (Retinol)** | MR Egger | 98 | 1.006(1.001,1.012) | 0.033 | 91.873 | 0.600 | -0.001 | 0.001 | 0.343 |
|  | Weighted median | 98 | 1.007(1.004,1.010) | <0.001 |  |  |  |  |  |
|  | Inverse variance weighted | 98 | 1.004(1.001,1.006) | 0.002 | 92.781 | 0.602 |  |  |  |
|  | Simple mode | 98 | 1.011(1.003,1.019) | 0.009 |  |  |  |  |  |
|  | Weighted mode | 98 | 1.010(1.003,1.017) | 0.006 |  |  |  |  |  |
| **Vitamin C (Ascorbate）** | MR Egger | 34 | 0.979(0.954,1.004) | 0.113 | 45.053 | 0.063 | 0.001 | 0.001 | 0.215 |
|  | Weighted median | 34 | 0.984(0.967,1.002) | 0.074 |  |  |  |  |  |
|  | Inverse variance weighted | 34 | 0.993(0.981,1.006) | 0.286 | 47.304 | 0.051 |  |  |  |
|  | Simple mode | 34 | 1.020(0.980,1.061) | 0.341 |  |  |  |  |  |
|  | Weighted mode | 34 | 0.986(0.966,1.007) | 0.210 |  |  |  |  |  |
| **α-tocopherol** | MR Egger | 41 | 1.016(0.933,1.106) | 0.725 | 22.671 | 0.983 | -0.001 | 0.001 | 0.133 |
|  | Weighted median | 41 | 0.956(0.906,1.008) | 0.096 |  |  |  |  |  |
|  | Inverse variance weighted | 41 | 0.957(0.921,0.993) | 0.021 | 25.023 | 0.969 |  |  |  |
|  | Simple mode | 41 | 0.954(0.867,1.050) | 0.340 |  |  |  |  |  |
|  | Weighted mode | 41 | 0.958(0.876,1.047) | 0.345 |  |  |  |  |  |

**Table S11 Causal effect, heterogeneity, pleiotropy of dietary and serum antioxidant vitamins on HDL in MR analysis**

| **Antioxidative vitamin** | **Method** | **No. SNP** | **OR (95% CI)** | ***P-*value** | **Heterogeneity** | | **Pleiotropy** | | |
| --- | --- | --- | --- | --- | --- | --- | --- | --- | --- |
|  |  |  |  |  | Q | *P-*value | Intercept | SE | *P-*value |
| **Dietary antioxidative vitamin** | |  |  |  |  |  |  |  |  |
| **Vitamin A** | MR Egger | 88 | 1.057(1.006,1.110) | 0.032 | 168.549 | <0.001 | -0.001 | 0.001 | 0.318 |
|  | Weighted median | 88 | 1.026(1.001,1.052) | 0.045 |  |  |  |  |  |
|  | Inverse variance weighted | 88 | 1.027(1.006,1.050) | 0.013 | 170.506 | <0.001 |  |  |  |
|  | Simple mode | 88 | 1.042(0.971,1.119) | 0.255 |  |  |  |  |  |
|  | Weighted mode | 88 | 1.038(0.971,1.108) | 0.275 |  |  |  |  |  |
| **Vitamin C** | MR Egger | 82 | 0.987(0.937,1.04) | 0.620 | 170.331 | <0.001 | 0.001 | 0.001 | 0.371 |
|  | Weighted median | 82 | 1.007(0.982,1.034) | 0.571 |  |  |  |  |  |
|  | Inverse variance weighted | 82 | 1.027(1.003,1.051) | 0.027 | 171.995 | <0.001 |  |  |  |
|  | Simple mode | 82 | 1.002(0.935,1.073) | 0.965 |  |  |  |  |  |
|  | Weighted mode | 82 | 1.002(0.941,1.066) | 0.962 |  |  |  |  |  |
| **Vitamin E** | MR Egger | 77 | 0.984(0.927,1.045) | 0.596 | 178.719 | <0.001 | <0.001 | 0.001 | 0.708 |
|  | Weighted median | 77 | 0.994(0.965,1.024) | 0.693 |  |  |  |  |  |
|  | Inverse variance weighted | 77 | 0.994(0.967,1.021) | 0.663 | 179.056 | <0.001 |  |  |  |
|  | Simple mode | 77 | 1.021(0.941,1.107) | 0.621 |  |  |  |  |  |
|  | Weighted mode | 77 | 1.021(0.950,1.097) | 0.577 |  |  |  |  |  |
| **Carotene** | MR Egger | 80 | 1.039(0.979,1.103) | 0.210 | 165.548 | <0.001 | <0.001 | 0.001 | 0.675 |
|  | Weighted median | 80 | 1.030(1.004,1.058) | 0.024 |  |  |  |  |  |
|  | Inverse variance weighted | 80 | 1.027(1.002,1.054) | 0.038 | 165.923 | <0.001 |  |  |  |
|  | Simple mode | 80 | 1.056(0.986,1.131) | 0.122 |  |  |  |  |  |
|  | Weighted mode | 80 | 1.056(0.989,1.128) | 0.109 |  |  |  |  |  |
| **Absolute circulating antioxidants** | |  |  |  |  |  |  |  |  |
| **Vitamin A (Retinol)** | Wald ratio | 1 | 0.906(0.797,1.029) | 0.129 |  |  |  |  |  |
| **Vitamin C (Ascorbate）** | MR Egger | 14 | 1.014(0.980,1.050) | 0.441 | 33.238 | 0.001 | -0.001 | 0.002 | 0.587 |
|  | Weighted median | 14 | 1.004(0.987,1.021) | 0.656 |  |  |  |  |  |
|  | Inverse variance weighted | 14 | 1.006(0.987,1.025) | 0.540 | 34.101 | 0.001 |  |  |  |
|  | Simple mode | 14 | 0.983(0.953,1.013) | 0.289 |  |  |  |  |  |
|  | Weighted mode | 14 | 1.011(0.996,1.026) | 0.179 |  |  |  |  |  |
| **α-Tocopherol** | Inverse variance weighted | 2 | 0.154(0.022,1.100) | 0.062 | 326.097 | <0.001 |  |  |  |
| **α-Carotene** | MR Egger | 3 | 1.059(0.677,1.658) | 0.843 | 19.798 | <0.001 | -0.004 | 0.022 | 0.886 |
|  | Weighted median | 3 | 1.043(1.005,1.083) | 0.027 |  |  |  |  |  |
|  | Inverse variance weighted | 3 | 1.017(0.940,1.101) | 0.669 | 20.452 | <0.001 |  |  |  |
|  | Simple mode | 3 | 1.042(0.998,1.087) | 0.200 |  |  |  |  |  |
|  | Weighted mode | 3 | 1.048(1.016,1.081) | 0.099 |  |  |  |  |  |
| **β-Carotene** | MR Egger | 4 | 1.023(0.904,1.159) | 0.751 | 16.717 | <0.001 | -0.002 | 0.009 | 0.836 |
|  | Weighted median | 4 | 1.011(0.994,1.028) | 0.210 |  |  |  |  |  |
|  | Inverse variance weighted | 4 | 1.009(0.973,1.047) | 0.625 | 17.181 | 0.001 |  |  |  |
|  | Simple mode | 4 | 1.017(0.996,1.039) | 0.217 |  |  |  |  |  |
|  | Weighted mode | 4 | 1.011(0.995,1.028) | 0.282 |  |  |  |  |  |
| **Circulating metabolite's concentation** | |  |  |  |  |  |  |  |  |
| **Vitamin A (Retinol)** | MR Egger | 88 | 1.003(0.987,1.018) | 0.743 | 765.609 | <0.001 | -0.003 | 0.002 | 0.098 |
|  | Weighted median | 88 | 1.000(0.995,1.004) | 0.838 |  |  |  |  |  |
|  | Inverse variance weighted | 88 | 0.991(0.984,0.998) | 0.011 | 790.556 | <0.001 |  |  |  |
|  | Simple mode | 88 | 1.002(0.992,1.012) | 0.662 |  |  |  |  |  |
|  | Weighted mode | 88 | 1.003(0.996,1.011) | 0.374 |  |  |  |  |  |
| **Vitamin C (Ascorbate）** | MR Egger | 33 | 1.007(0.962,1.055) | 0.763 | 178.931 | <0.001 | 0.001 | 0.002 | 0.738 |
|  | Weighted median | 33 | 1.016(0.998,1.034) | 0.084 |  |  |  |  |  |
|  | Inverse variance weighted | 33 | 1.014(0.990,1.039) | 0.259 | 179.589 | <0.001 |  |  |  |
|  | Simple mode | 33 | 0.999(0.970,1.029) | 0.967 |  |  |  |  |  |
|  | Weighted mode | 33 | 1.012(0.996,1.028) | 0.163 |  |  |  |  |  |
| **α-tocopherol** | MR Egger | 39 | 0.923(0.436,1.953) | 0.836 | 2451.297 | <0.001 | 0.006 | 0.008 | 0.490 |
|  | Weighted median | 39 | 1.017(0.954,1.084) | 0.602 |  |  |  |  |  |
|  | Inverse variance weighted | 39 | 1.174(0.848,1.624) | 0.334 | 2483.526 | <0.001 |  |  |  |
|  | Simple mode | 39 | 0.995(0.873,1.135) | 0.946 |  |  |  |  |  |
|  | Weighted mode | 39 | 0.980(0.859,1.118) | 0.764 |  |  |  |  |  |

**Table S12 Causal effect, heterogeneity, pleiotropy of dietary and serum antioxidant vitamins on triglyceride in MR analysis**

| **Antioxidative vitamin** | **Method** | **No. SNP** | **OR (95% CI)** | ***P-*value** | **Heterogeneity** | | **Pleiotropy** | | |
| --- | --- | --- | --- | --- | --- | --- | --- | --- | --- |
|  |  |  |  |  | Q | *P-*value | Intercept | SE | *P-*value |
| **Dietary antioxidative vitamin** | |  |  |  |  |  |  |  |  |
| **Vitamin A** | MR Egger | 88 | 1.004(0.952,1.060) | 0.877 | 159.942 | <0.001 | -0.001 | 0.001 | 0.389 |
|  | Weighted median | 88 | 0.988(0.961,1.015) | 0.362 |  |  |  |  |  |
|  | Inverse variance weighted | 88 | 0.976(0.954,0.999) | 0.040 | 161.322 | <0.001 |  |  |  |
|  | Simple mode | 88 | 1.018(0.947,1.095) | 0.628 |  |  |  |  |  |
|  | Weighted mode | 88 | 1.015(0.945,1.089) | 0.686 |  |  |  |  |  |
| **Vitamin C** | MR Egger | 85 | 1.006(0.942,1.075) | 0.856 | 224.962 | <0.001 | -0.002 | 0.001 | 0.216 |
|  | Weighted median | 85 | 0.978(0.951,1.007) | 0.133 |  |  |  |  |  |
|  | Inverse variance weighted | 85 | 0.969(0.941,0.998) | 0.034 | 229.172 | <0.001 |  |  |  |
|  | Simple mode | 85 | 0.961(0.896,1.031) | 0.274 |  |  |  |  |  |
|  | Weighted mode | 85 | 1.003(0.937,1.073) | 0.93 |  |  |  |  |  |
| **Vitamin E** | MR Egger | 77 | 0.983(0.921,1.050) | 0.618 | 195.159 | <0.001 | <0.001 | 0.001 | 0.861 |
|  | Weighted median | 77 | 0.989(0.960,1.018) | 0.434 |  |  |  |  |  |
|  | Inverse variance weighted | 77 | 0.989(0.960,1.018) | 0.45 | 195.239 | <0.001 |  |  |  |
|  | Simple mode | 77 | 0.971(0.906,1.040) | 0.403 |  |  |  |  |  |
|  | Weighted mode | 77 | 0.975(0.913,1.041) | 0.455 |  |  |  |  |  |
| **Carotene** | MR Egger | 80 | 1.002(0.940,1.069) | 0.942 | 179.986 | <0.001 | -0.001 | 0.001 | 0.528 |
|  | Weighted median | 80 | 0.982(0.955,1.010) | 0.213 |  |  |  |  |  |
|  | Inverse variance weighted | 80 | 0.984(0.957,1.011) | 0.24 | 180.915 | <0.001 |  |  |  |
|  | Simple mode | 80 | 0.947(0.879,1.020) | 0.154 |  |  |  |  |  |
|  | Weighted mode | 80 | 0.970(0.902,1.043) | 0.418 |  |  |  |  |  |
| **Absolute circulating antioxidants** | |  |  |  |  |  |  |  |  |
| **Vitamin A (Retinol)** | Wald ratio | 1 | 1.118(0.978,1.277) | 0.102 |  |  |  |  |  |
| **Vitamin C (Ascorbate）** | MR Egger | 14 | 0.984(0.950,1.019) | 0.374 | 31.635 | 0.002 | 0.001 | 0.002 | 0.562 |
|  | Weighted median | 14 | 0.988(0.970,1.006) | 0.192 |  |  |  |  |  |
|  | Inverse variance weighted | 14 | 0.992(0.973,1.012) | 0.432 | 32.572 | 0.002 |  |  |  |
|  | Simple mode | 14 | 0.992(0.964,1.021) | 0.596 |  |  |  |  |  |
|  | Weighted mode | 14 | 0.988(0.973,1.004) | 0.166 |  |  |  |  |  |
| **α-Tocopherol** | Inverse variance weighted | 2 | 69.639(0.288,16842.275) | 0.130 | 2337.148 | <0.001 |  |  |  |
| **α-Carotene** | MR Egger | 3 | 1.001(0.783,1.279) | 0.997 | 5.342 | 0.021 | -0.003 | 0.012 | 0.83 |
|  | Weighted median | 3 | 0.964(0.917,1.014) | 0.155 |  |  |  |  |  |
|  | Inverse variance weighted | 3 | 0.968(0.926,1.012) | 0.149 | 5.744 | 0.057 |  |  |  |
|  | Simple mode | 3 | 0.947(0.906,0.990) | 0.137 |  |  |  |  |  |
|  | Weighted mode | 3 | 0.955(0.917,0.994) | 0.154 |  |  |  |  |  |
| **β-Carotene** | MR Egger | 4 | 0.962(0.919,1.007) | 0.236 | 1.798 | 0.407 | 0.002 | 0.003 | 0.558 |
|  | Weighted median | 4 | 0.975(0.957,0.993) | 0.006 |  |  |  |  |  |
|  | Inverse variance weighted | 4 | 0.977(0.961,0.992) | 0.004 | 2.283 | 0.516 |  |  |  |
|  | Simple mode | 4 | 0.973(0.949,0.998) | 0.127 |  |  |  |  |  |
|  | Weighted mode | 4 | 0.975(0.956,0.993) | 0.077 |  |  |  |  |  |
| **Circulating metabolite's concentation** | |  |  |  |  |  |  |  |  |
| **Vitamin A (Retinol)** | MR Egger | 88 | 0.991(0.978,1.004) | 0.157 | 487.774 | <0.001 | 0.006 | 0.002 | 0 |
|  | Weighted median | 88 | 1.002(0.998,1.006) | 0.319 |  |  |  |  |  |
|  | Inverse variance weighted | 88 | 1.012(1.006,1.019) | <0.001 | 565.137 | <0.001 |  |  |  |
|  | Simple mode | 88 | 1.004(0.996,1.011) | 0.383 |  |  |  |  |  |
|  | Weighted mode | 88 | 1.003(0.996,1.010) | 0.443 |  |  |  |  |  |
| **Vitamin C (Ascorbate）** | MR Egger | 33 | 0.993(0.955,1.033) | 0.736 | 118.451 | <0.001 | <0.001 | 0.002 | 0.86 |
|  | Weighted median | 33 | 0.988(0.969,1.007) | 0.202 |  |  |  |  |  |
|  | Inverse variance weighted | 33 | 0.990(0.970,1.011) | 0.347 | 118.573 | <0.001 |  |  |  |
|  | Simple mode | 33 | 0.999(0.968,1.031) | 0.945 |  |  |  |  |  |
|  | Weighted mode | 33 | 0.990(0.974,1.007) | 0.259 |  |  |  |  |  |
| **α-tocopherol** | MR Egger | 39 | 1.003(0.711,1.415) | 0.987 | 475.716 | <0.001 | 0.001 | 0.004 | 0.759 |
|  | Weighted median | 39 | 0.983(0.919,1.051) | 0.610 |  |  |  |  |  |
|  | Inverse variance weighted | 39 | 1.053(0.908,1.221) | 0.496 | 476.941 | <0.001 |  |  |  |
|  | Simple mode | 39 | 0.999(0.885,1.128) | 0.989 |  |  |  |  |  |
|  | Weighted mode | 39 | 0.977(0.875,1.091) | 0.680 |  |  |  |  |  |

**Table S13 Causal effect, heterogeneity, pleiotropy of dietary and serum antioxidant vitamins on hypertension in MR analysis**

| **Antioxidative vitamin** | **Method** | **No. SNP** | **OR (95% CI)** | ***P-*value** | **Heterogeneity** | | **Pleiotropy** | | |
| --- | --- | --- | --- | --- | --- | --- | --- | --- | --- |
|  |  |  |  |  | Q | *P-*value | Intercept | SE | *P-*value |
| **Dietary antioxidative vitamin** | |  |  |  |  |  |  |  |  |
| **Vitamin A** | MR Egger | 93 | 0.987(0.972,1.003) | 0.110 | 128.661 | 0.006 | <0.001 | <0.001 | 0.731 |
|  | Weighted median | 93 | 0.990(0.981,0.998) | 0.012 |  |  |  |  |  |
|  | Inverse variance weighted | 93 | 0.990(0.983,0.996) | 0.002 | 128.828 | 0.007 |  |  |  |
|  | Simple mode | 93 | 0.997(0.975,1.020) | 0.826 |  |  |  |  |  |
|  | Weighted mode | 93 | 0.997(0.978,1.017) | 0.802 |  |  |  |  |  |
| **Vitamin C** | MR Egger | 86 | 0.992(0.977,1.007) | 0.312 | 109.794 | 0.031 | <0.001 | <0.001 | 0.753 |
|  | Weighted median | 86 | 0.993(0.985,1.002) | 0.135 |  |  |  |  |  |
|  | Inverse variance weighted | 86 | 0.990(0.983,0.996) | 0.003 | 109.924 | 0.036 |  |  |  |
|  | Simple mode | 86 | 1.004(0.980,1.028) | 0.750 |  |  |  |  |  |
|  | Weighted mode | 86 | 1.004(0.981,1.027) | 0.741 |  |  |  |  |  |
| **Vitamin E** | MR Egger | 83 | 1.001(0.987,1.015) | 0.925 | 90.913 | 0.212 | <0.001 | <0.001 | 0.117 |
|  | Weighted median | 83 | 0.994(0.985,1.002) | 0.132 |  |  |  |  |  |
|  | Inverse variance weighted | 83 | 0.991(0.985,0.997) | 0.005 | 93.723 | 0.177 |  |  |  |
|  | Simple mode | 83 | 1.006(0.984,1.028) | 0.624 |  |  |  |  |  |
|  | Weighted mode | 83 | 1.006(0.984,1.028) | 0.591 |  |  |  |  |  |
| **Carotene** | MR Egger | 83 | 1.007(0.991,1.024) | 0.377 | 106.434 | 0.031 | <0.001 | <0.001 | 0.216 |
|  | Weighted median | 83 | 1.001(0.992,1.010) | 0.805 |  |  |  |  |  |
|  | Inverse variance weighted | 83 | 0.998(0.991,1.005) | 0.567 | 108.474 | 0.027 |  |  |  |
|  | Simple mode | 83 | 1.008(0.985,1.031) | 0.509 |  |  |  |  |  |
|  | Weighted mode | 83 | 1.006(0.983,1.029) | 0.602 |  |  |  |  |  |
| **Absolute circulating antioxidants** | |  |  |  |  |  |  |  |  |
| **Vitamin A (Retinol)** | Inverse variance weighted | 2 | 0.991(0.96,1.022) | 0.557 | 0.162 | 0.688 |  |  |  |
| **Vitamin C (Ascorbate）** | MR Egger | 14 | 1.000(0.993,1.007) | 0.990 | 10.551 | 0.568 | <0.001 | <0.001 | 0.876 |
|  | Weighted median | 14 | 1.002(0.996,1.008) | 0.555 |  |  |  |  |  |
|  | Inverse variance weighted | 14 | 1.000(0.995,1.004) | 0.833 | 10.576 | 0.646 |  |  |  |
|  | Simple mode | 14 | 0.998(0.987,1.010) | 0.761 |  |  |  |  |  |
|  | Weighted mode | 14 | 1.002(0.996,1.007) | 0.580 |  |  |  |  |  |
| **α-Tocopherol** | MR Egger | 3 | 1.167(0.934,1.457) | 0.405 | 0.391 | 0.532 | -0.003 | 0.004 | 0.547 |
|  | Weighted median | 3 | 1.061(0.989,1.137) | 0.096 |  |  |  |  |  |
|  | Inverse variance weighted | 3 | 1.059(1.028,1.090) | <0.001 | 1.134 | 0.567 |  |  |  |
|  | Simple mode | 3 | 1.070(1.013,1.129) | 0.135 |  |  |  |  |  |
|  | Weighted mode | 3 | 1.071(1.016,1.129) | 0.124 |  |  |  |  |  |
| **α-Carotene** | MR Egger | 3 | 0.995(0.960,1.031) | 0.837 | 0.287 | 0.592 | 0.001 | 0.002 | 0.789 |
|  | Weighted median | 3 | 1.001(0.992,1.011) | 0.785 |  |  |  |  |  |
|  | Inverse variance weighted | 3 | 1.001(0.993,1.010) | 0.772 | 0.405 | 0.817 |  |  |  |
|  | Simple mode | 3 | 1.002(0.990,1.015) | 0.782 |  |  |  |  |  |
|  | Weighted mode | 3 | 1.001(0.991,1.012) | 0.816 |  |  |  |  |  |
| **β-Carotene** | MR Egger | 4 | 1.007(0.992,1.022) | 0.469 | 0.079 | 0.961 | -0.001 | 0.001 | 0.559 |
|  | Weighted median | 4 | 1.002(0.997,1.008) | 0.421 |  |  |  |  |  |
|  | Inverse variance weighted | 4 | 1.002(0.996,1.007) | 0.507 | 0.563 | 0.905 |  |  |  |
|  | Simple mode | 4 | 0.996(0.988,1.004) | 0.393 |  |  |  |  |  |
|  | Weighted mode | 4 | 1.002(0.996,1.009) | 0.494 |  |  |  |  |  |
| **Circulating metabolite's concentation** | |  |  |  |  |  |  |  |  |
| **Vitamin A (Retinol)** | MR Egger | 83 | 1.001(0.999,1.003) | 0.229 | 78.848 | 0.547 | <0.001 | <0.001 | 0.057 |
|  | Weighted median | 83 | 0.999(0.998,1.000) | 0.099 |  |  |  |  |  |
|  | Inverse variance weighted | 83 | 1.000(0.999,1.000) | 0.247 | 82.57 | 0.462 |  |  |  |
|  | Simple mode | 83 | 0.999(0.996,1.002) | 0.349 |  |  |  |  |  |
|  | Weighted mode | 83 | 0.999(0.996,1.001) | 0.278 |  |  |  |  |  |
| **Vitamin C (Ascorbate）** | MR Egger | 34 | 1.006(0.997,1.014) | 0.191 | 46.763 | 0.045 | <0.001 | <0.001 | 0.496 |
|  | Weighted median | 34 | 1.002(0.997,1.008) | 0.406 |  |  |  |  |  |
|  | Inverse variance weighted | 34 | 1.003(0.999,1.007) | 0.153 | 47.457 | 0.049 |  |  |  |
|  | Simple mode | 34 | 1.005(0.993,1.018) | 0.420 |  |  |  |  |  |
|  | Weighted mode | 34 | 1.002(0.997,1.008) | 0.392 |  |  |  |  |  |
| **α-tocopherol** | MR Egger | 41 | 0.996(0.959,1.034) | 0.830 | 56.155 | 0.037 | <0.001 | <0.001 | 0.678 |
|  | Weighted median | 41 | 0.986(0.965,1.007) | 0.184 |  |  |  |  |  |
|  | Inverse variance weighted | 41 | 0.989(0.973,1.005) | 0.170 | 56.407 | 0.044 |  |  |  |
|  | Simple mode | 41 | 0.967(0.919,1.017) | 0.197 |  |  |  |  |  |
|  | Weighted mode | 41 | 0.976(0.932,1.022) | 0.300 |  |  |  |  |  |

**Table S14 Causal effect, heterogeneity, pleiotropy of dietary and serum antioxidant vitamins on obesity in MR analysis**

| **Antioxidative vitamin** | **Method** | **No. SNP** | **OR (95% CI)** | ***P-*value** | **Heterogeneity** | | **Pleiotropy** | | |
| --- | --- | --- | --- | --- | --- | --- | --- | --- | --- |
|  |  |  |  |  | Q | *P-*value | Intercept | SE | *P-*value |
| **Dietary antioxidative vitamin** | |  |  |  |  |  |  |  |  |
| **Vitamin A** | MR Egger | 93 | 0.983(0.932,1.037) | 0.529 | 201.817 | <0.001 | <0.001 | 0.001 | 0.779 |
|  | Weighted median | 93 | 0.979(0.956,1.003) | 0.089 |  |  |  |  |  |
|  | Inverse variance weighted | 93 | 0.976(0.955,0.999) | 0.037 | 201.994 | <0.001 |  |  |  |
|  | Simple mode | 93 | 0.990(0.923,1.062) | 0.780 |  |  |  |  |  |
|  | Weighted mode | 93 | 0.989(0.928,1.053) | 0.720 |  |  |  |  |  |
| **Vitamin C** | MR Egger | 86 | 1.004(0.946,1.065) | 0.900 | 247.442 | <0.001 | -0.001 | 0.001 | 0.477 |
|  | Weighted median | 86 | 0.988(0.964,1.013) | 0.359 |  |  |  |  |  |
|  | Inverse variance weighted | 86 | 0.973(0.947,0.998) | 0.037 | 248.927 | <0.001 |  |  |  |
|  | Simple mode | 86 | 0.979(0.913,1.05) | 0.554 |  |  |  |  |  |
|  | Weighted mode | 86 | 0.981(0.922,1.044) | 0.550 |  |  |  |  |  |
| **Vitamin E** | MR Egger | 82 | 0.989(0.927,1.055) | 0.732 | 311.595 | <0.001 | -0.001 | 0.001 | 0.598 |
|  | Weighted median | 82 | 0.997(0.973,1.022) | 0.813 |  |  |  |  |  |
|  | Inverse variance weighted | 82 | 0.964(0.935,0.993) | 0.016 | 312.676 | <0.001 |  |  |  |
|  | Simple mode | 82 | 1.011(0.957,1.069) | 0.695 |  |  |  |  |  |
|  | Weighted mode | 82 | 1.009(0.956,1.065) | 0.747 |  |  |  |  |  |
| **Carotene** | MR Egger | 83 | 0.978(0.929,1.031) | 0.414 | 149.694 | <0.001 | <0.001 | 0.001 | 0.848 |
|  | Weighted median | 83 | 0.982(0.959,1.006) | 0.146 |  |  |  |  |  |
|  | Inverse variance weighted | 83 | 0.974(0.953,0.995) | 0.017 | 149.763 | <0.001 |  |  |  |
|  | Simple mode | 83 | 0.983(0.924,1.045) | 0.582 |  |  |  |  |  |
|  | Weighted mode | 83 | 0.984(0.928,1.044) | 0.602 |  |  |  |  |  |
| **Absolute circulating antioxidants** | |  |  |  |  |  |  |  |  |
| **Vitamin A (Retinol)** | Inverse variance weighted | 2 | 1.013(0.912,1.126) | 0.803 | 1.527 | 0.217 |  |  |  |
| **Vitamin C (Ascorbate）** | MR Egger | 14 | 0.984(0.963,1.005) | 0.151 | 13.968 | 0.303 | 0.001 | 0.001 | 0.494 |
|  | Weighted median | 14 | 0.989(0.971,1.007) | 0.235 |  |  |  |  |  |
|  | Inverse variance weighted | 14 | 0.990(0.978,1.001) | 0.084 | 14.547 | 0.336 |  |  |  |
|  | Simple mode | 14 | 0.993(0.967,1.021) | 0.642 |  |  |  |  |  |
|  | Weighted mode | 14 | 0.987(0.972,1.001) | 0.097 |  |  |  |  |  |
| **α-Tocopherol** | MR Egger | 3 | 0.892(0.490,1.622) | 0.771 | 0.007 | 0.936 | 0.004 | 0.010 | 0.736 |
|  | Weighted median | 3 | 1.023(0.925,1.131) | 0.661 |  |  |  |  |  |
|  | Inverse variance weighted | 3 | 1.019(0.941,1.104) | 0.644 | 0.201 | 0.904 |  |  |  |
|  | Simple mode | 3 | 1.035(0.930,1.151) | 0.595 |  |  |  |  |  |
|  | Weighted mode | 3 | 1.033(0.933,1.144) | 0.594 |  |  |  |  |  |
| **α-Carotene** | MR Egger | 3 | 0.991(0.900,1.090) | 0.879 | 0.010 | 0.922 | <0.001 | 0.005 | 0.934 |
|  | Weighted median | 3 | 0.996(0.968,1.024) | 0.768 |  |  |  |  |  |
|  | Inverse variance weighted | 3 | 0.996(0.972,1.019) | 0.711 | 0.020 | 0.990 |  |  |  |
|  | Simple mode | 3 | 0.998(0.965,1.031) | 0.899 |  |  |  |  |  |
|  | Weighted mode | 3 | 0.994(0.965,1.024) | 0.736 |  |  |  |  |  |
| **β-Carotene** | MR Egger | 4 | 1.011(0.971,1.053) | 0.644 | 0.458 | 0.795 | -0.002 | 0.003 | 0.504 |
|  | Weighted median | 4 | 0.997(0.981,1.013) | 0.734 |  |  |  |  |  |
|  | Inverse variance weighted | 4 | 0.996(0.981,1.010) | 0.543 | 1.109 | 0.775 |  |  |  |
|  | Simple mode | 4 | 0.997(0.977,1.017) | 0.774 |  |  |  |  |  |
|  | Weighted mode | 4 | 0.998(0.982,1.015) | 0.843 |  |  |  |  |  |
| **Circulating metabolite's concentation** | |  |  |  |  |  |  |  |  |
| **Vitamin A (Retinol)** | MR Egger | 87 | 0.993(0.986,1.000) | 0.051 | 164.573 | <0.001 | 0.002 | 0.001 | 0.025 |
|  | Weighted median | 87 | 0.998(0.995,1.001) | 0.183 |  |  |  |  |  |
|  | Inverse variance weighted | 87 | 1.000(0.997,1.003) | 0.903 | 174.616 | <0.001 |  |  |  |
|  | Simple mode | 87 | 0.995(0.988,1.002) | 0.193 |  |  |  |  |  |
|  | Weighted mode | 87 | 0.995(0.988,1.001) | 0.107 |  |  |  |  |  |
| **Vitamin C (Ascorbate）** | MR Egger | 34 | 0.986(0.949,1.024) | 0.458 | 143.162 | <0.001 | 0.001 | 0.002 | 0.562 |
|  | Weighted median | 34 | 0.988(0.972,1.004) | 0.130 |  |  |  |  |  |
|  | Inverse variance weighted | 34 | 0.995(0.975,1.015) | 0.628 | 144.698 | <0.001 |  |  |  |
|  | Simple mode | 34 | 1.000(0.964,1.038) | 0.991 |  |  |  |  |  |
|  | Weighted mode | 34 | 0.988(0.973,1.003) | 0.135 |  |  |  |  |  |
| **α-tocopherol** | MR Egger | 41 | 0.939(0.845,1.043) | 0.245 | 60.303 | 0.016 | <0.001 | 0.001 | 0.953 |
|  | Weighted median | 41 | 0.945(0.892,1.002) | 0.057 |  |  |  |  |  |
|  | Inverse variance weighted | 41 | 0.936(0.894,0.979) | 0.004 | 60.308 | 0.021 |  |  |  |
|  | Simple mode | 41 | 0.92(0.813,1.042) | 0.195 |  |  |  |  |  |
|  | Weighted mode | 41 | 0.949(0.861,1.047) | 0.303 |  |  |  |  |  |

**Table S15 Multivariate MR for causality association of dietary antioxidant vitamins on metabolic syndrome and its components**

| **Outcome** | **Exposure** |  | **β** | **SE** | **OR (95% CI)** | **P** |
| --- | --- | --- | --- | --- | --- | --- |
| **Metabolic syndrome** |  |  |  |  |  |  |
|  | **Vitamin A** | **Dietary vitamin A** | -0.092 | 0.039 | 0.912(0.845,0.984) | 0.018 |
|  |  | **Supplement vitamin A** | 0.121 | 0.743 | 1.128(0.263,4.837) | 0.871 |
|  | **Vitamin C** | **Dietary vitamin C** | -0.099 | 0.045 | 0.906(0.829,0.989) | 0.028 |
|  |  | **Supplement vitamin C** | -0.735 | 0.339 | 0.479(0.247,0.932) | 0.03 |
|  | **Vitamin E** | **Dietary vitamin E** | 0.006 | 0.047 | 1.006(0.917,1.104) | 0.898 |
|  |  | **Supplement vitamin E** | 0.845 | 0.706 | 2.327(0.583,9.286) | 0.232 |
| **Blood glucose elevating** |  |  |  |  |  |  |
|  | **Vitamin A** | **Dietary vitamin A** | 0.016 | 0.010 | 1.017(0.996,1.037) | 0.111 |
|  |  | **Supplement vitamin A** | -0.093 | 0.190 | 0.912(0.629,1.322) | 0.626 |
|  | **Vitamin C** | **Dietary vitamin C** | -0.009 | 0.011 | 0.991(0.969,1.013) | 0.432 |
|  |  | **Supplement vitamin C** | -0.018 | 0.087 | 0.982(0.827,1.165) | 0.833 |
|  | **Vitamin E** | **Dietary vitamin E** | <0.001 | 0.012 | 1.000(0.978,1.023) | 0.984 |
|  |  | **Supplement vitamin E** | -0.345 | 0.175 | 0.708(0.502,0.998) | 0.048 |
| **HDL decreasing** |  |  |  |  |  |  |
|  | **Vitamin A** | **Dietary vitamin A** | 0.022 | 0.015 | 1.023(0.994,1.052) | 0.128 |
|  |  | **Supplement vitamin A** | -0.099 | 0.277 | 0.906(0.527,1.558) | 0.721 |
|  | **Vitamin C** | **Dietary vitamin C** | 0.024 | 0.017 | 1.024(0.990,1.060) | 0.161 |
|  |  | **Supplement vitamin C** | 0.220 | 0.133 | 1.246(0.960,1.616) | 0.098 |
|  | **Vitamin E** | **Dietary vitamin E** | -0.009 | 0.016 | 0.991(0.961,1.022) | 0.564 |
|  |  | **Supplement vitamin E** | -0.183 | 0.236 | 0.833(0.525,1.322) | 0.438 |
| **Triglyceride elevating** |  |  |  |  |  |  |
|  | **Vitamin A** | **Dietary vitamin A** | -0.050 | 0.015 | 0.951(0.923,0.980) | 0.001 |
|  |  | **Supplement vitamin A** | -0.054 | 0.293 | 0.947(0.534,1.681) | 0.854 |
|  | **Vitamin C** | **Dietary vitamin C** | -0.024 | 0.017 | 0.976(0.944,1.009) | 0.16 |
|  |  | **Supplement vitamin C** | -0.198 | 0.131 | 0.820(0.634,1.060) | 0.13 |
|  | **Vitamin E** | **Dietary vitamin E** | -0.021 | 0.022 | 0.980(0.939,1.022) | 0.339 |
|  |  | **Supplement vitamin E** | 0.398 | 0.325 | 1.488(0.787,2.814) | 0.221 |
| **Blood pressure elevating** |  |  |  |  |  |  |
|  | **Vitamin A** | **Dietary vitamin A** | -0.010 | 0.004 | 0.990(0.982,0.998) | 0.014 |
|  |  | **Supplement vitamin A** | -0.011 | 0.077 | 0.989(0.851,1.149) | 0.884 |
|  | **Vitamin C** | **Dietary vitamin C** | -0.009 | 0.004 | 0.991(0.983,0.998) | 0.015 |
|  |  | **Supplement vitamin C** | -0.004 | 0.029 | 0.996(0.940,1.055) | 0.883 |
|  | **Vitamin E** | **Dietary vitamin E** | -0.007 | 0.004 | 0.993(0.986,1.001) | 0.097 |
|  |  | **Supplement vitamin E** | -0.034 | 0.06 | 0.967(0.859,1.088) | 0.578 |
| **Obesity** |  |  |  |  |  |  |
|  | **Vitamin A** | **Dietary vitamin A** | -0.017 | 0.014 | 0.983(0.956,1.011) | 0.23 |
|  |  | **Supplement vitamin A** | -0.240 | 0.273 | 0.786(0.461,1.342) | 0.378 |
|  | **Vitamin C** | **Dietary vitamin C** | -0.038 | 0.015 | 0.963(0.935,0.992) | 0.011 |
|  |  | **Supplement vitamin C** | -0.152 | 0.114 | 0.859(0.687,1.075) | 0.183 |
|  | **Vitamin E** | **Dietary vitamin E** | -0.014 | 0.019 | 0.986(0.950,1.023) | 0.448 |
|  |  | **Supplement vitamin E** | 0.168 | 0.285 | 1.183(0.677,2.068) | 0.555 |


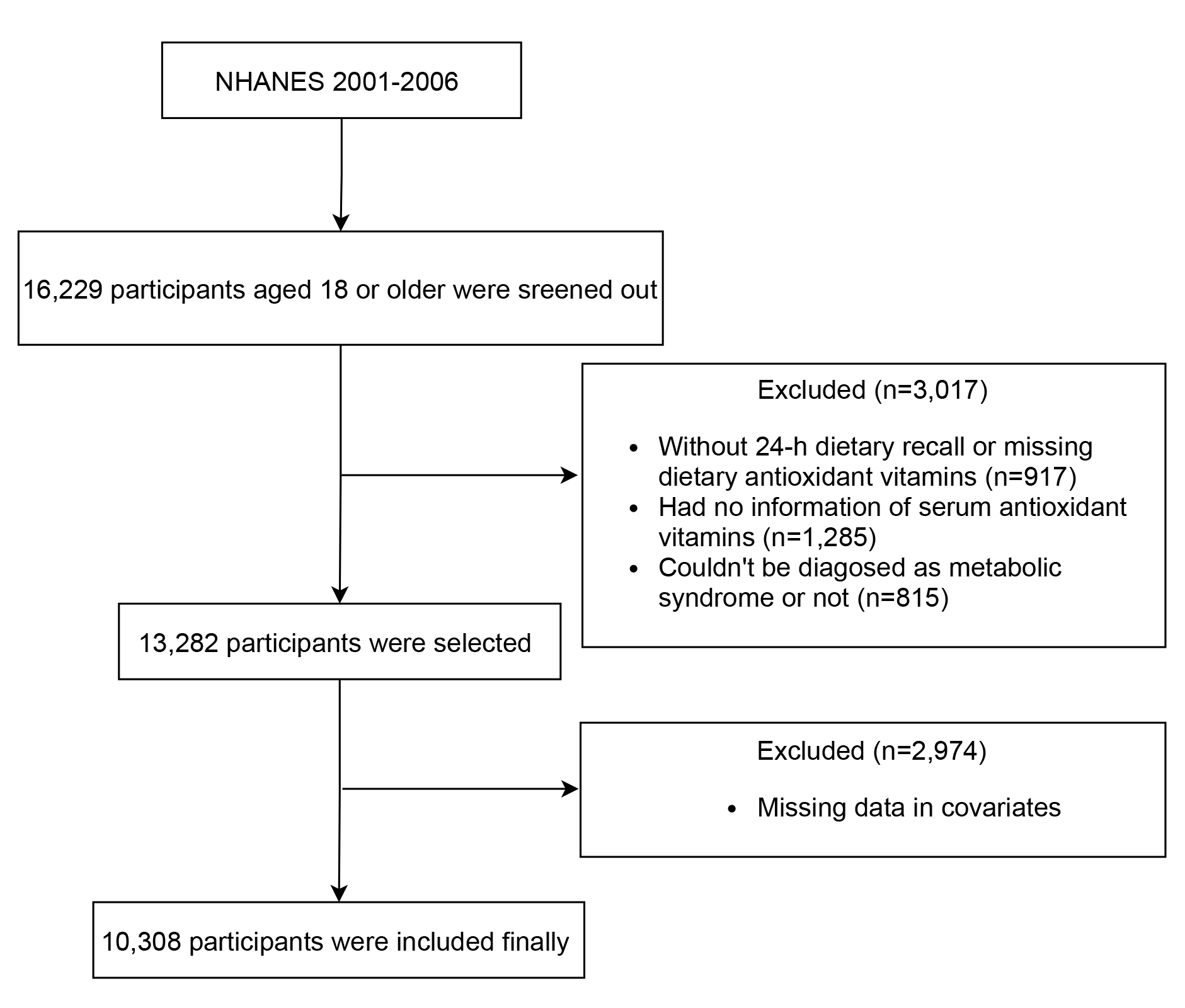


**Figure S1 Flow chart for the selection of participants in NHANES.**


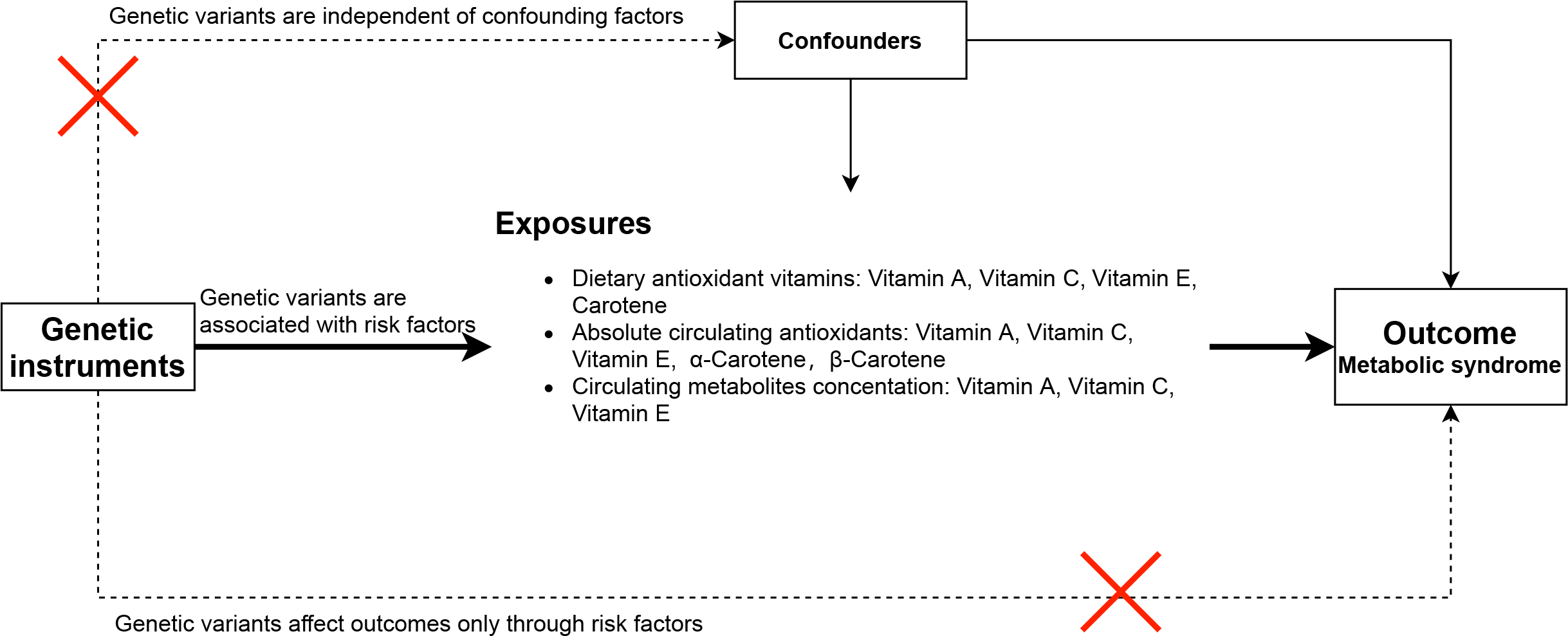


**Figure S2 MR assumptions of this study**


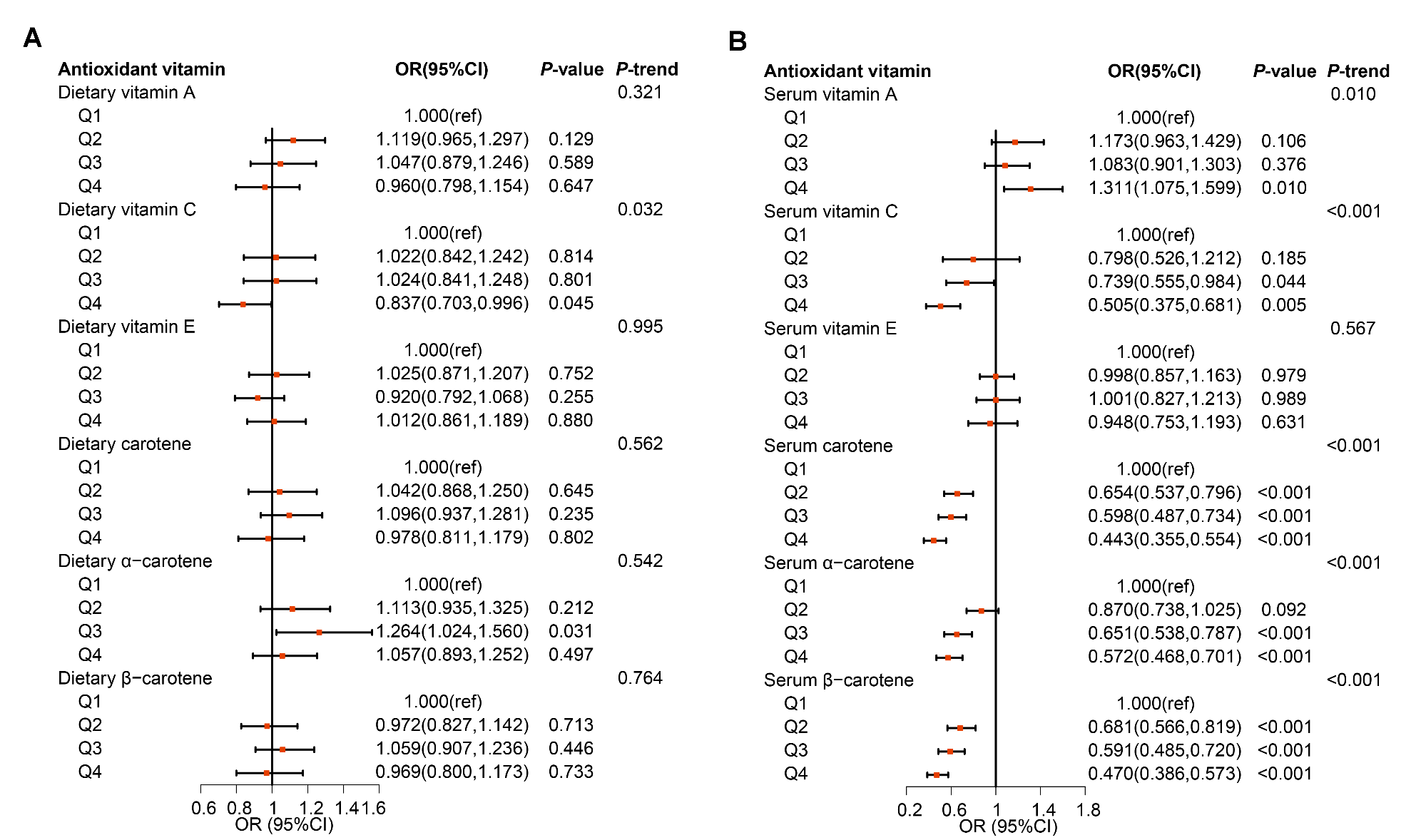


**Figure S3 Observational associations of dietary (A) and serum (B) antioxidant vitamins on blood glucose elevating in NHANES population**


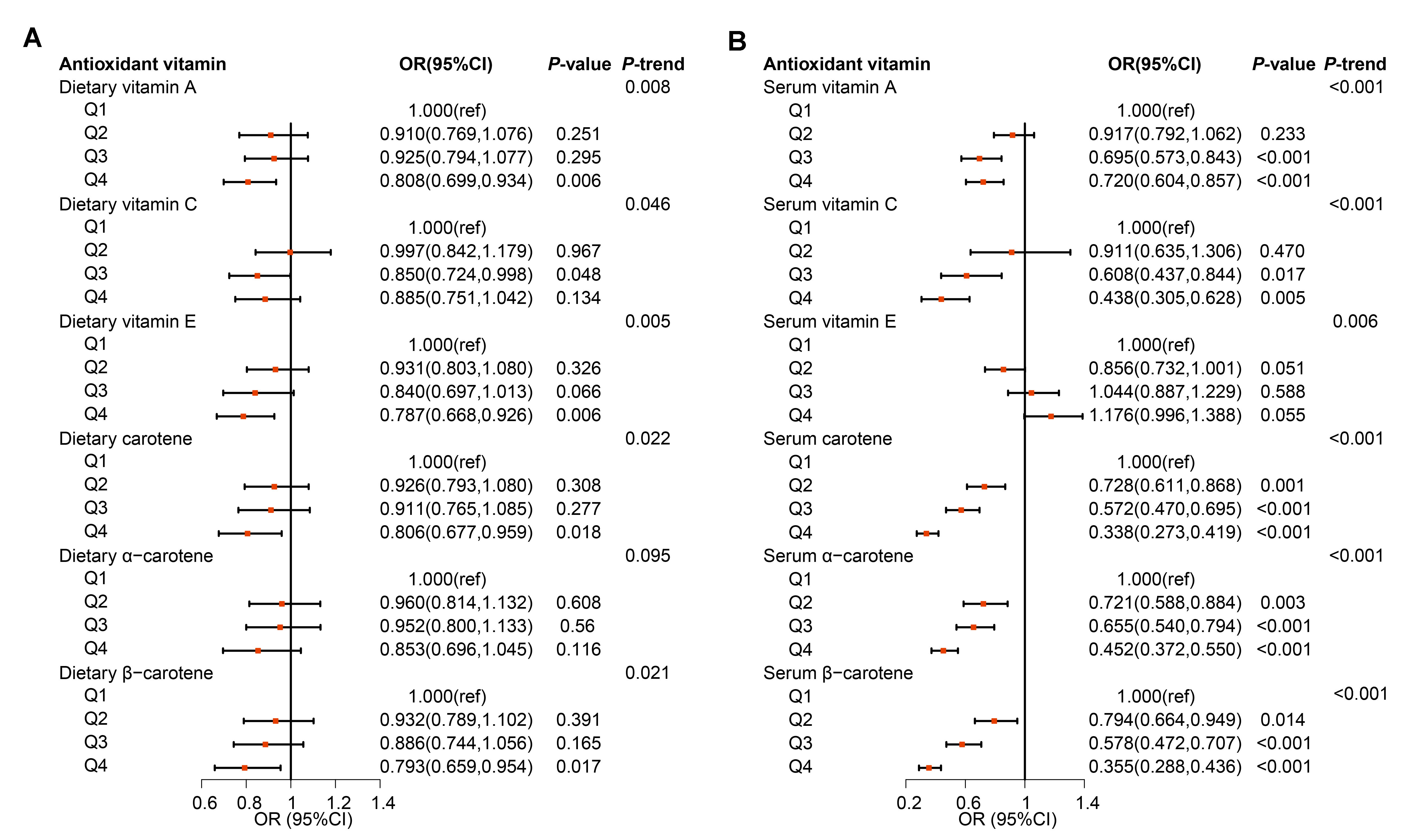


**Figure S4 Observational associations of dietary (A) and serum (B) antioxidant vitamins on HDL decreasing in NHANES population**
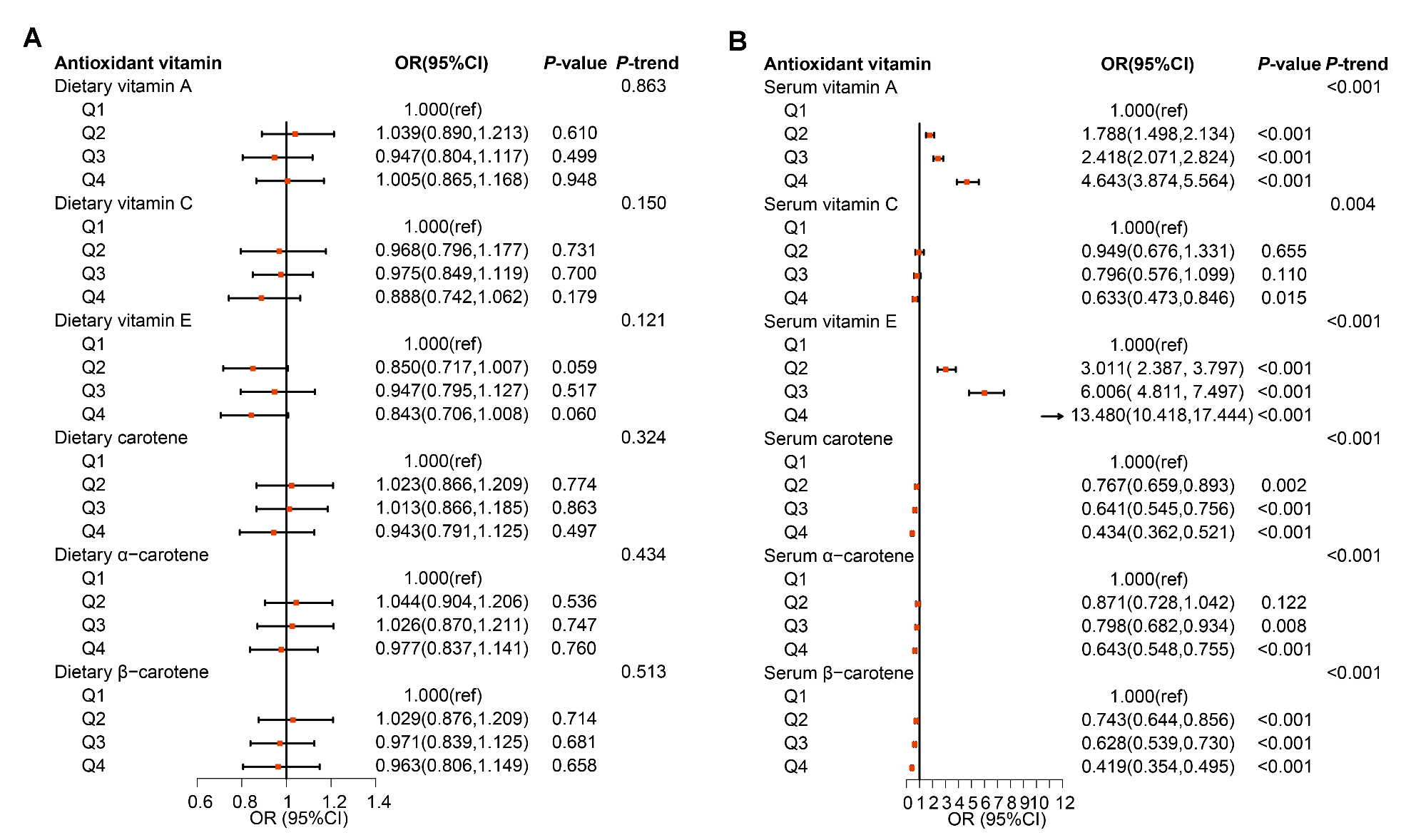


**Figure S5 Observational associations of dietary (A) and serum (B) antioxidant vitamins on triglyceride elevating in NHANES population**


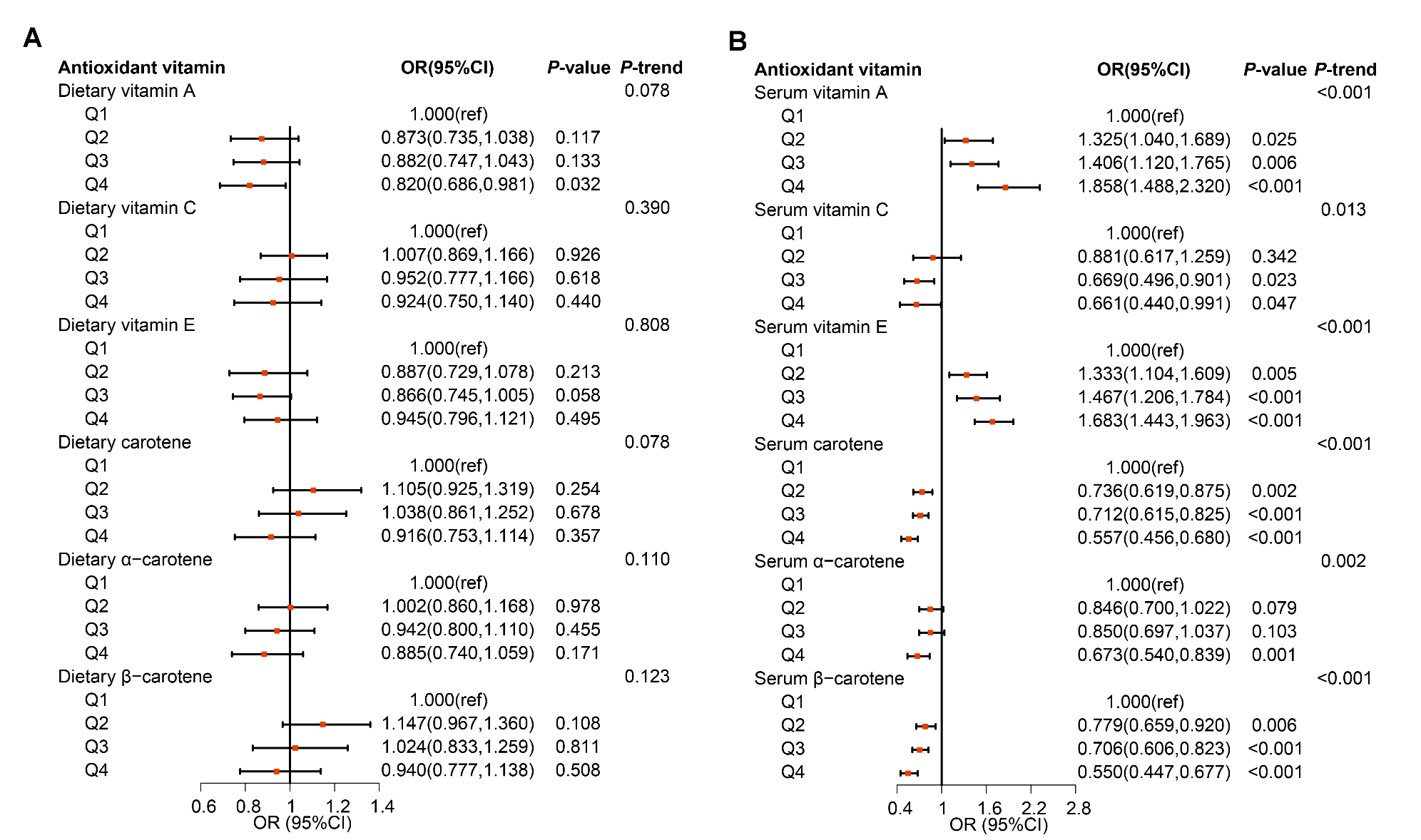


**Figure S6 Observational associations of dietary (A) and serum (B) antioxidant vitamins on blood pressure elevating in NHANES population**


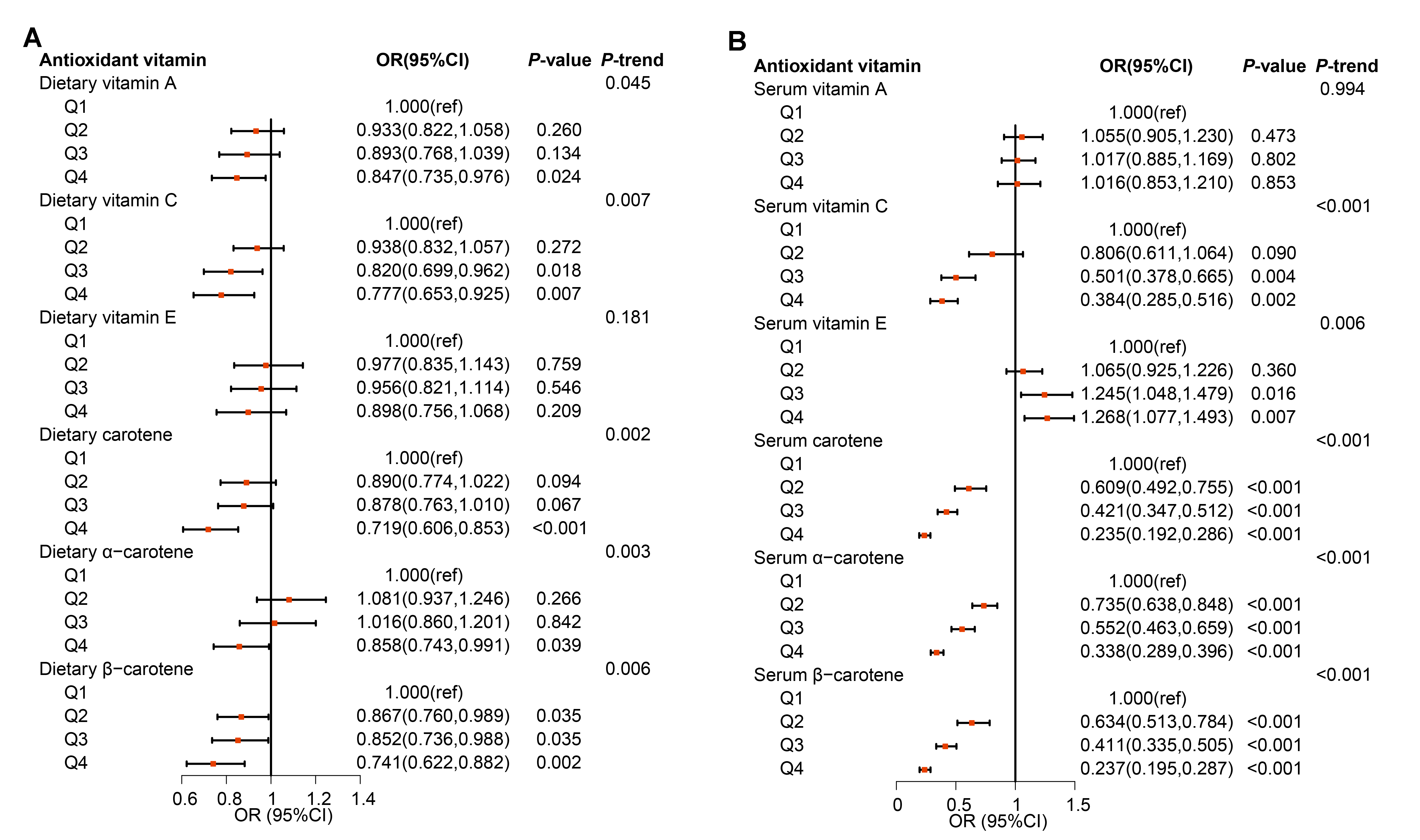


**Figure S7 Observational associations of dietary (A) and serum (B) antioxidant vitamins on obesity in NHANES population**


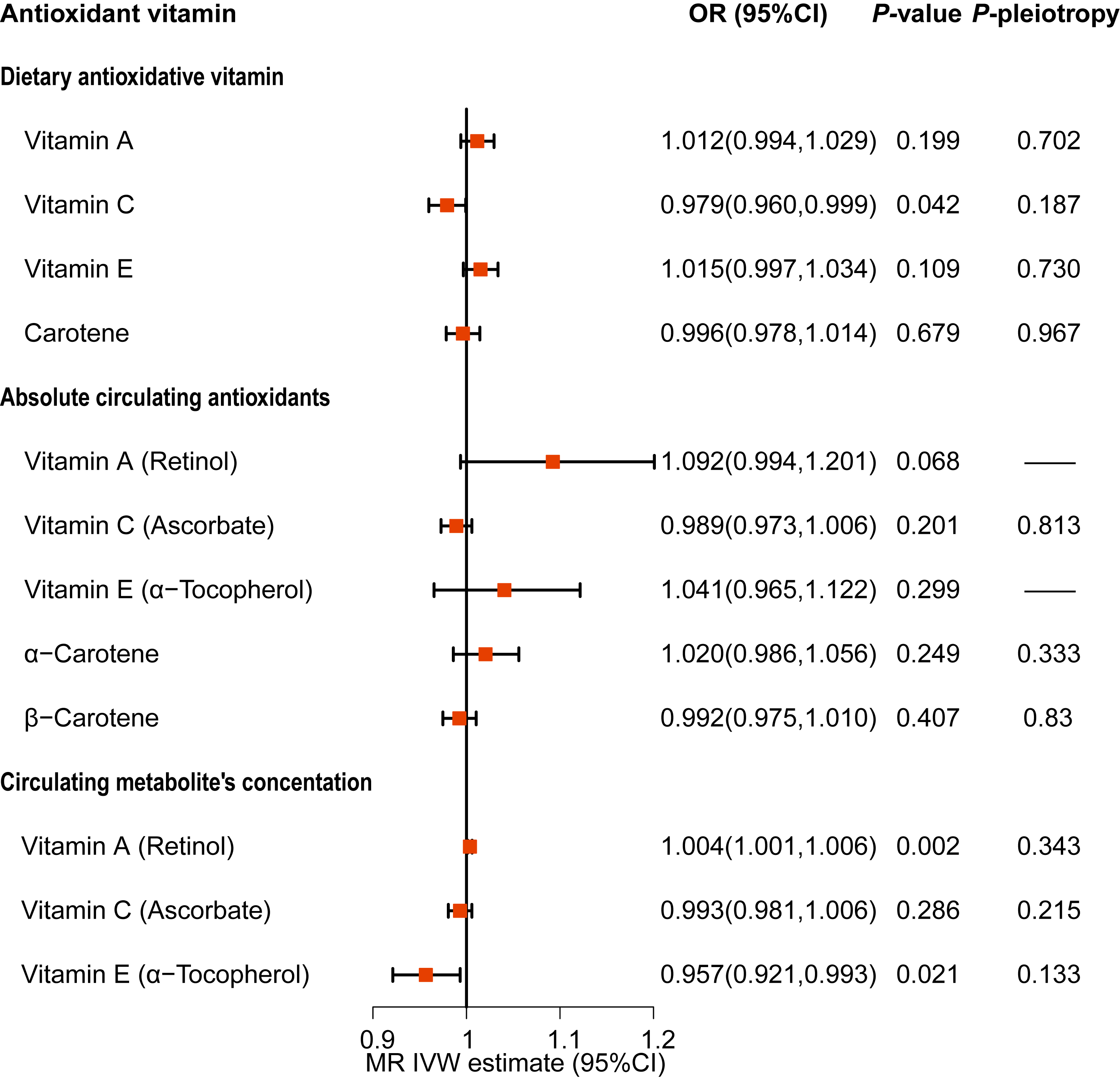


**Figure S8 Causality association of dietary and serum antioxidant vitamins on blood glucose in MR analysis**

**
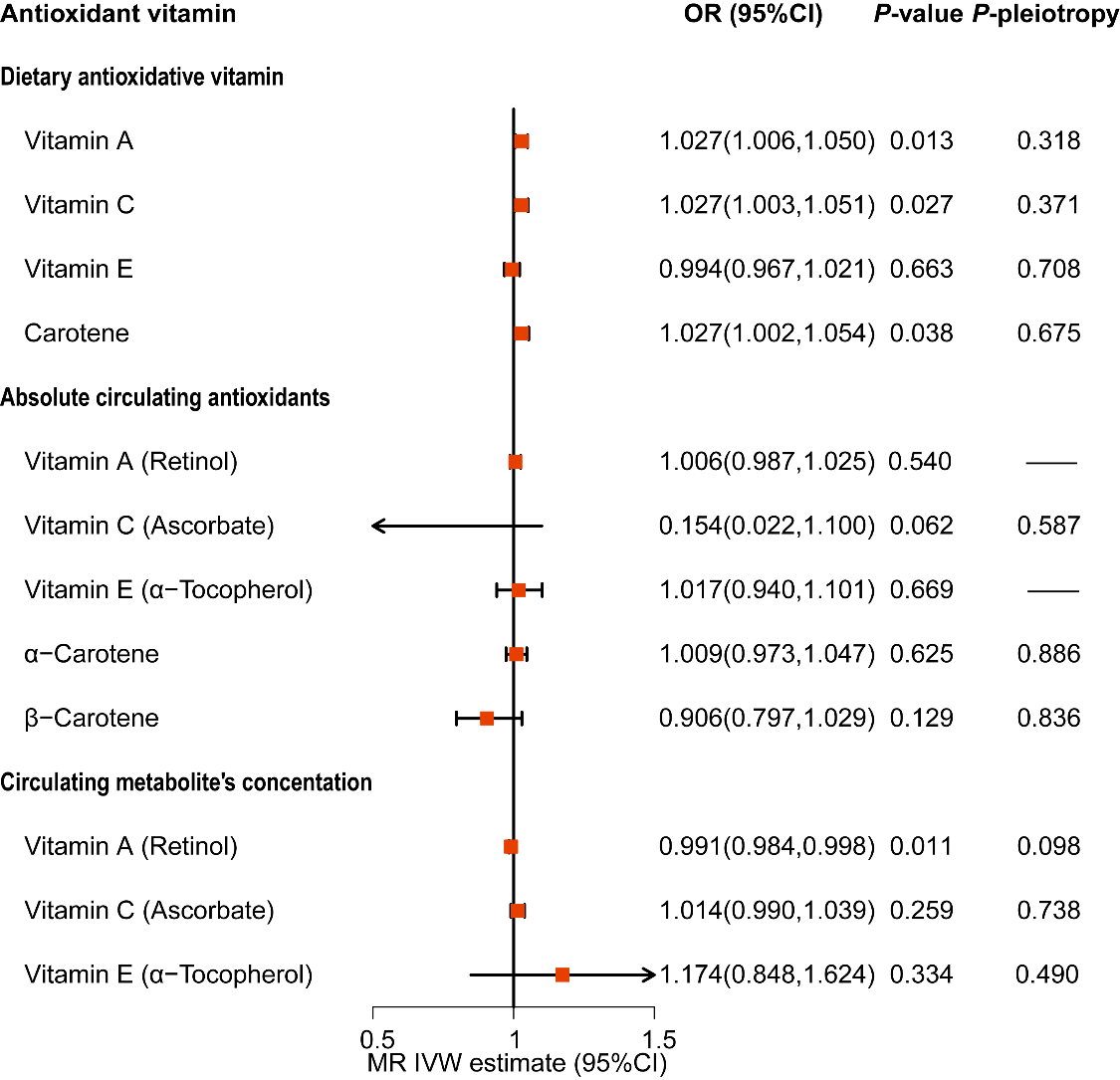
**

**Figure S9 Causality association of dietary and serum antioxidant vitamins on HDL in MR analysis**

**
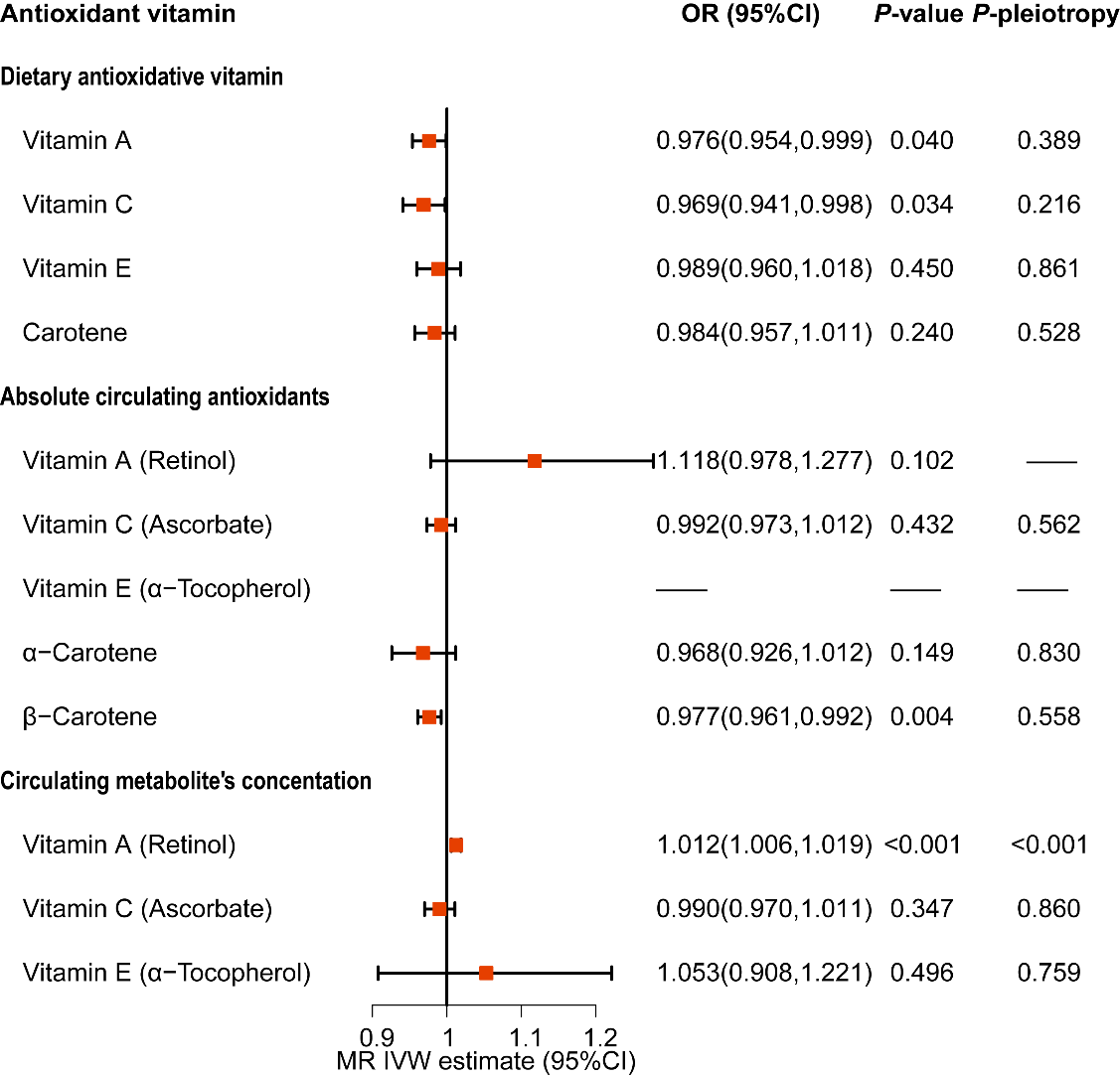
**

**Figure S10 Causality association of dietary and serum antioxidant vitamins on triglyceride in MR analysis**

**
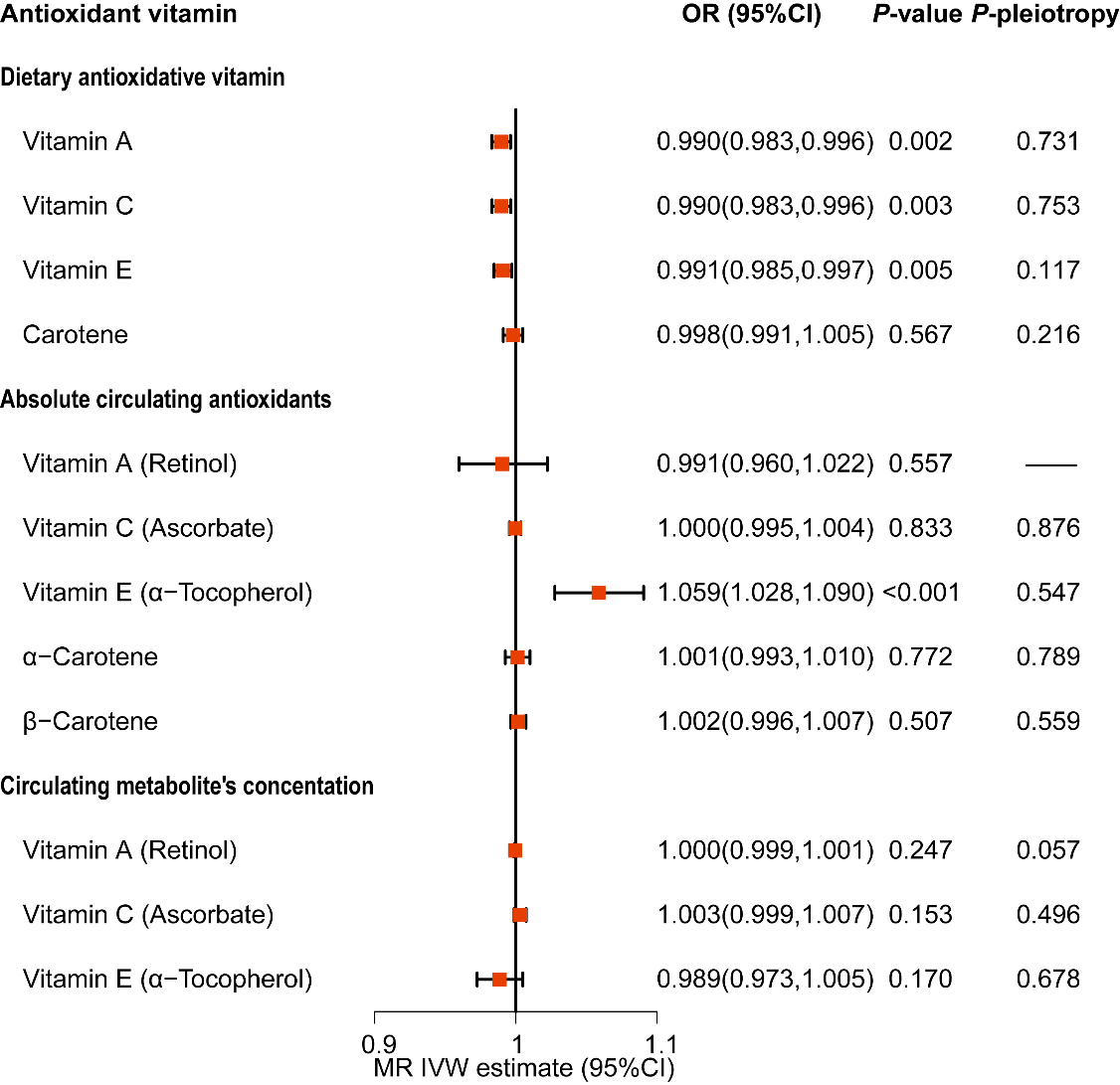
**

**Figure S11 Causality association of dietary and serum antioxidant vitamins on hypertension in MR analysis**


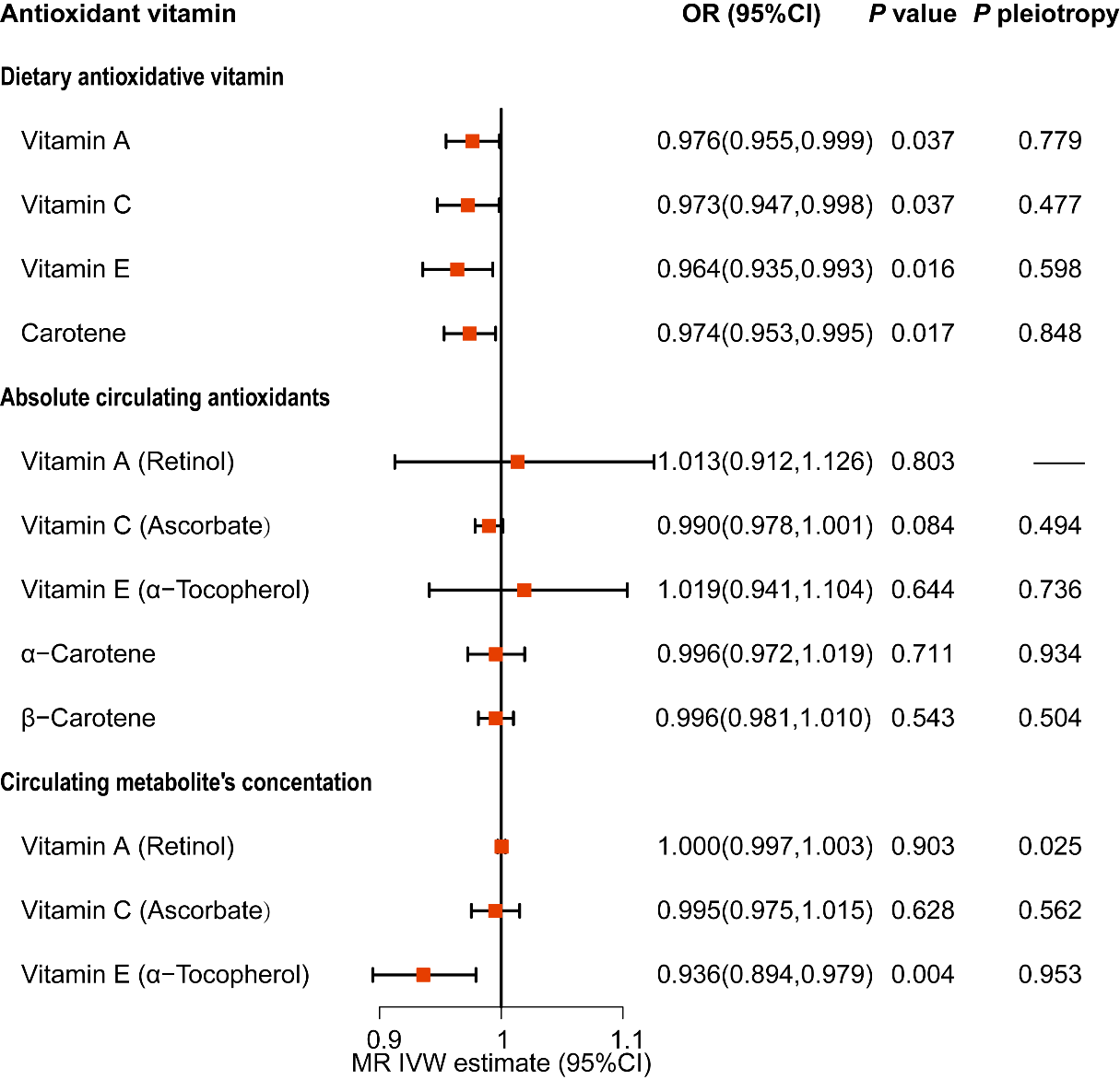


**Figure S12 Causality association of dietary and serum antioxidant vitamins on obesity in MR analysis**
